# Improving life quality for the aged: a comprehensive post-occupancy evaluation of long-term care facilities in China

**DOI:** 10.3389/fpubh.2024.1488653

**Published:** 2024-11-28

**Authors:** Yu Chen, Jiamin Zhang, Zhuohang Yang, Zhi Qiu

**Affiliations:** ^1^Institute of Architectural Design and Theoretical Research, Zhejiang University, Hangzhou, China; ^2^Center for Balance Architecture, Zhejiang University, Hangzhou, China

**Keywords:** long-term care facilities, physical environment, post-occupancy evaluation, assessment tool, satisfaction survey

## Abstract

**Introduction:**

In response to the call for active aging, improving the quality of residential environments for older people is becoming a worldwide concern. Over the past decade, China has witnessed a significant increase in the construction of long-term care facilities (LTCFs) catering to the older adult with physical and mental limitations. However, the fast pace of LTCF development has led to substandard physical environments that compromise the quality of life for older individuals. Consequently, there is an urgent need to conduct comprehensive post-occupancy evaluations (POEs) in order to systematically assess the current state of LTCF physical environments and identify prevailing problems therein.

**Methods:**

This study conducted POEs on 37 existing LTCFs from both objective and subjective perspectives, using self-developed environmental assessment tools and user satisfaction questionnaires.

**Results:**

The results reveal substantial room for improvement within LTCF physical environments, particularly concerning outdoor areas, resident rooms and staff spaces. The psychological needs of residents and working requirements of staff are not adequately addressed or supported.

**Discussion:**

A total of ninety-two typical problems are identified across eight categories, and relevant causes associated with programming, design, construction, and operation are discussed. The findings are expected to serve as warnings for future designs, provide empirical evidence for the revision of relevant standards, and promote the sustainable development of LTCF construction.

## 1 Introduction

The global population is currently experiencing the megatrend of aging (1). According to recent reports, the number of older adult individuals unable to meet their basic needs was estimated to be at least 142 million, with the prevalence of dementia exceeding 55 million (2, 3). These individuals face a series of complex health challenges (4, 5). Providing high-quality long-term care (LTC) for this vulnerable group and fulfilling the WHO’s vision for active aging presents a global challenge (6). Existing evidence suggests that long-term care facilities (LTCFs) can provide professional care for older adult individuals with functional limitations and health risks, rendering them optimal settings for geriatric care (7). Furthermore, it has been shown that the physical environment of LTCFs has a significant impact on the resident’s physical and mental well-being (8–10). Therefore, ensuring a suitable LTCF environment that caters to the specific needs of the older adult is pivotal in guaranteeing their overall quality of life.

Back in the 1970s, as European and American countries were expanding their construction of nursing homes, experts noticed an excessive institutionalization of the physical environment. This created a gloomy, isolated, and discontented atmosphere for older adult residents, leading to detrimental effects on their physical and mental health (11). Consequently, the optimization of the built environment in older adult residential facilities toward ensuring a pleasant living environment for senior citizens has garnered significant attention. Many experts and scholars have started to apply the Post-Occupancy Evaluation (POE) method, which involves collecting actual usage data and feedback from users, to identify existing issues and implement targeted improvements and optimizations, thereby enhancing the living environment for the older adult (12–15). During the 1980s and 1990s, a series of environmental assessment tools were developed based on the Press-Competence Model proposed by Lawton, which emphasizes the significant relationship between the physical and mental states of the older adult and their spatial environment (16). Subsequently, numerous studies have integrated scale assessments with subjective surveys to conduct systematic POE investigations on existing older adult residential buildings (17). For example, Hung (18) conducted individual interviews with staff and employed the Dining Environment Assessment Protocol (DEAP) to evaluate the dining environment in LTCFs, thereby identifying issues pertaining to mealtime experiences. Toit (19) conducted a comprehensive evaluation of residential aged care facilities in accordance with the Residential Environment Impact Scale (REIS), focusing on exploring the impact of the environment on residents’ selectivity and social engagement. Nordin (20) assessed 20 residential care facilities across various regions in Sweden, aiming to explore current deficiencies in terms of environmental design elements and principles through an analysis of the Sheffield Care Environment Assessment Matrix (Swedish version) (S-SCEAM) scores obtained and interviews conducted at two selected facilities. Wahlroos (21) translated the S-SCEAM into a Finnish version (S-SCEAM-Fin) and validated it in 20 LTC units in Finland, exploring domains with lower scores that required improvement. In general, Western countries have established a feedback cycle of “evaluation-design” by incorporating insights from evaluations into subsequent design phases, thereby continuously enhancing the physical environment quality of LTCFs.

Over the past decade, China and other developing countries facing severe aging issues have swiftly established a substantial number of LTCFs to cater to the escalating demand for LTC (22). However, the rapid pace of LTCF construction has led to a noticeable decline in quality of the physical environments. Identified problems include neglecting basic principles such as fall prevention, aimed at reducing residents’ safety (23), having a hospital–like decoration style that makes people feel depressed (24), or lacking a support area for staff, such as a cleaning room, which degrades service quality and efficiency (25). These issues pose difficulties in meeting residential living needs and staff service requirements within the physical environment, thereby compromising residents’ quality of life. Many facilities have become trapped in a “poor quality-low occupancy-financial loss” cycle, which significantly affects the supply of LTC services (26). In the future, renovating existing projects, drawing on the experience gained, and providing guidance for future construction and the revision of standards will be the main tasks in enhancing LTC quality (27). It is urgent to conduct systematic POEs to review the current status, identify prevailing problems, and leverage accumulated knowledge.

However, in contrast to Western practices of identifying issues through POEs, Chinese studies lack effective evaluation methods and practices (28). The specific deficiencies include the following three aspects: (1) Perspective: Previous studies set in China pay more attention to evaluating the layout and distribution of care facilities from the planning perspective (29, 30) or focus on building performance, acoustic environment, lighting environment, thermal environment, and energy consumption (31, 32). However, there are a limited number of studies that assess the level of alignment between the built environment and user needs from the perspective of users. (2) Methods: In contrast to Western research, which places emphasis on subjective feedback from diverse user groups (33), the prevailing research in China predominantly relies on objective methodologies such as photography and instrumental measurements, with limited consideration given to subjective feedback from users, particularly staff members (28). (3) Tools: Despite there being 23 widely used assessment tools globally for evaluating older adult living environments (16), they are culturally specific, reflecting diverse legal, care, and aging contexts, and thus are not one-size-fits-all (21, 34). For instance, because of varying degrees of aging, tools predominantly tailored for cognitive impairment support in Western countries often fall short for China’s older adult care facilities. These facilities need to accommodate a wide spectrum of needs, from self-reliant individuals to those with severe disabilities (35, 36). Current research has demonstrated that existing assessment tools have some other shortcomings, including being overly specialized and complicated (37), biased toward large facilities (35), and not keeping pace with contemporary developments and care theories (16, 38). In China, there are two relevant standards, namely the Guidelines of Classification and Accreditation for Senior Care Organization and the Standard for Design of Care Facilities for the Older Adults (39, 40), that can be used to assess environmental quality of LTCFs, but their evaluation criteria mainly focus on aspects such as barrier-free design and environmental sanitation that meet the basic physiological needs of residents; they do not pay much attention to the older adult’s psychological needs and staff’s needs. Moreover, the development of above standards is not sufficiently scientific and rigorous; the indicators often rely on the subjective judgment of the authors and experts, which may not necessarily align with users’ needs (36). Owing to these deficiencies, China faces an absence of robust POEs, perpetuating recurrent mismatches between environmental design and user needs, which leads to the waste of spatial, temporal, and financial resources (41).

**Figure 1:**
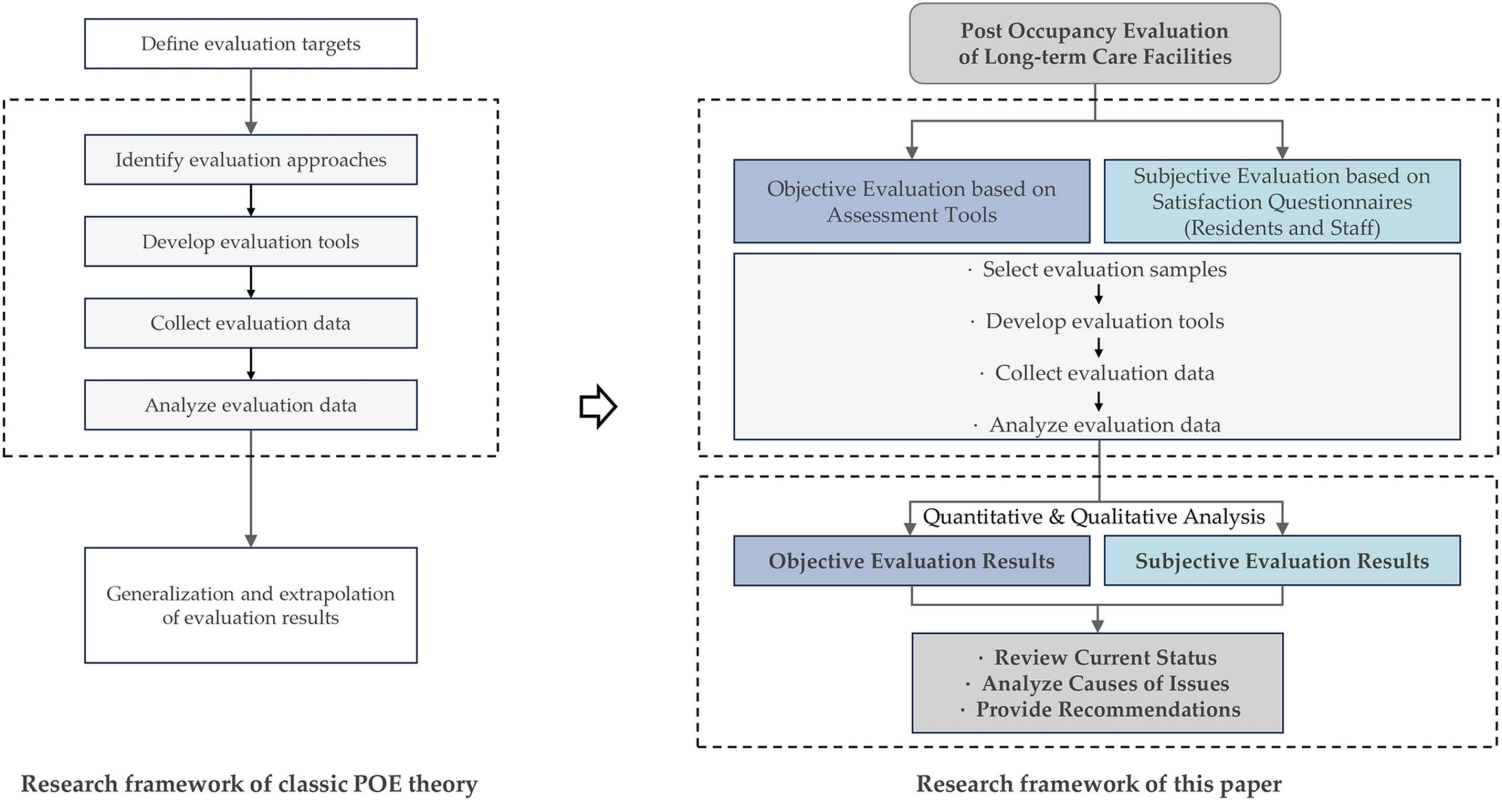
Research framework.

Therefore, based on the demand for POEs and on existing research gaps, this study explored POE practices to discover the extent to which the physical environment caters to user requirements, encompassing two components: (1) conducting nationwide objective evaluations based on a Chinese-version customized assessment tool focused on user demands; (2) administering satisfaction surveys among diverse user groups, including the older adult and caregivers, to gather subjective assessment data. Through the analysis of both objective and subjective evaluation data, this study aimed to achieve the following objectives: (1) obtain an overall understanding of the current state of physical environments in Chinese LTCFs and investigate specific environmental problems within different domains; (2) critically analyze underlying causes; (3) provide recommendations for future design practices and revision of design guidelines. Based on the classic POE theory by Preiser (42), the research framework of this paper is shown in Figure 1. The results are expected to provide valuable insights and directions for future constructions, ultimately fostering sustainable development within LTCFs and thus improving life quality for the aged.

## 2 Materials and methods

### 2.1 Evaluation samples

#### 2.1.1 Objective evaluation samples

Considering the current operational characteristics of LTCFs in China, their service targets include a wide range of older adult individuals, from self-sufficient to severely disabled (43, 44). Therefore, this study focused on such comprehensive facilities. Specialized facilities for dementia care and hospice care were not the main focus of this study. The research team collected hundreds of comprehensive LTCF samples and has constructed a sample database based on field studies conducted across China over the past 5 years. In order to understand the current state of LTCF physical environments through POEs, and to ensure that the objective evaluation results could more comprehensively reflect the current construction and occupancy status of different types of LTCFs in China, this study took into account the differences in factors such as opening years, locations, project scales, occupancy rates, and construction forms and finally selected 37 projects from the sample database for a detailed, objective evaluation. Detailed information about the samples can be found in Supplementary Material A.

#### 2.1.2 Subjective evaluation samples

When selecting samples for further satisfaction survey, in addition to considering the factors mentioned above, differences in the results of objective evaluations were also taken into account. From the aforementioned 37 samples, 12 were selected for their varying levels of physical environmental quality, while also taking into consideration the convenience of the research. Residents and staff members were two main types of users from whom to collect opinions, the later including caregivers, administrators, medical staff, catering staff, etc. Personal factors such as gender, age, job, and duration of residence (employment) were considered when selecting specific user samples. Residents were selected with the premise of having basic cognitive and expressive abilities, while also considering varying physical conditions to obtain more reliable and comprehensive data. Following the convenience sampling strategy, a total of 195 samples were obtained with the permission and assistance of the LTCF operators, residents and staff. Tables 1, 2 present the basic personal information of the survey respondents.

**Table 1:** Basic personal information of the surveyed residents (*n* = 92).

Gender	Number	Age	Number
Male	40	60 ~ 69 years old	2
		70 ~ 79 years old	12
Female	52	80 ~ 89 years old	58
		90 years old and above	20

Education level	Number	Physical health status	Number	Duration of residence	Number
Junior high school or below	20	Independent	37	Within half a year	18
High school	19	Mild Functional Loss	24	Half a year to 1 year	12
College	46	Moderate Functional Loss	19	1 to 2 years	20
Postgraduate or above	0	Severe Functional Loss	9	2 to 3 years	19
No answer	7	Dementia (mild)	3	More than 3 years	23

**Table 2:** Basic personal information of the surveyed staff (*n* = 103).

Gender	Number	Education level	Number
Male	21	Junior high school or below	45
		High school	20
Female	82	College	32
		Postgraduate or above	6

Age	Number	Duration of employment	Number	Position	Number	Position	Number
18–30 years old	26	Within half a year	9	Administrator	4	Medical staff	7
31–40 years old	13	Half a year to 1 year	11	Nursing supervisor	13	Caregiver	60
41–50 years old	34	1 to 3 years	34	Social worker	7	Administrative staff	5
51–60 years old	27	3 to 5 years	24	Marketing staff	3	Catering staff	2
60 years old and above	3	More than 5 years	25	Support work staff (such as security staff, janitorial staff, and laundry staff)			2

### 2.2 Evaluation tools

#### 2.2.1 Objective evaluation tool

Environmental assessment scales or checklists are typically used for the objective evaluation of the physical environment in older adult residential buildings (45–48). Given the strong regional characteristics and adaptation issues of existing foreign instruments, coupled with the absence of a systematic environmental assessment tool based on user needs in China, the authors of this study have developed a localized assessment tool. This tool is founded on two theoretical bases: the Press-Competence Model previously discussed and Person-Centered Care (PCC), which is considered the optimal approach for improving the quality of life for older adults (49). Both theories highlight the importance of user needs in the design and evaluation of the physical environment. The instrument integrates common methods from international scales and has been successfully published in a top-tier Chinese academic journal (50). The main methods and steps involved in the development of the tool are outlined below.

1. Literature Review: Existing assessment tools, scales, empirical studies, reviews, guidelines and other relevant literature were referenced. Key points of environment design for user needs were extracted and revised as evaluation indicators through international references (translated and adapted from existing environment assessment tools), local references (derived from Chinese policy documents, design standards and codes), as well as integration and composition (comprehensive analysis of different categories of references). The specific references and methods can be found in Supplementary Material B3.
2. Field Research: Preliminary assessments were conducted in 10 LTCFs to validate the applicability and interrater reliability of evaluation indicators and ensure their adaptation to the Chinese context.
3. Expert Survey: Fifteen experts from the fields of gerontological architecture research, older adult care facility design, and older adult care project management and operations were consulted to obtain their opinions on the content validity of evaluation indicators.

The final assessment tool contains 160 indicators, covering 24 environmental design elements and addressing 14 environmental design principles (see Supplementary Material B4). For example, the indicator, *“In double rooms and multiple rooms, each bed is equipped with curtains or is partitioned by furniture for privacy,”* assesses whether the resident room (environmental design element) meets the privacy needs of residents (environmental design principle). Each indicator is required to be scored “0 or 1,” with certain indicators allowing for a “0.5” score to differentiate the degree. The scoring rate of indicators (total score /160*100%) is calculated as the project final score, which serves as a reflection of how effectively the physical environment fulfills user needs.

#### 2.2.2 Subjective evaluation tool

The subjective satisfaction survey was conducted based on a questionnaire, including the following three domains (Supplementary Materials B1, B2).

1. Respondent’s personal information: Age, gender, duration of stay, etc.
2. Satisfaction survey: Respondents were asked to rate their comfort level with given items using a 5-point Likert scale, ranging from 1 (very unsatisfied) to 5 (very satisfied). Items assessed in staff’s survey were 24 environmental design elements mentioned above, while some less relevant items like the central kitchen, staff living space, etc., were removed in the resident’s survey, leaving 17 items. Additionally, the satisfaction scale included a *“reasons for scoring”* section to collect respondent’s feedback for further analysis.
3. Overall Satisfaction: Respondents were asked to assess the overall satisfaction level using a 5-point Likert scale.

### 2.3 Evaluation process

**Figure 2:**
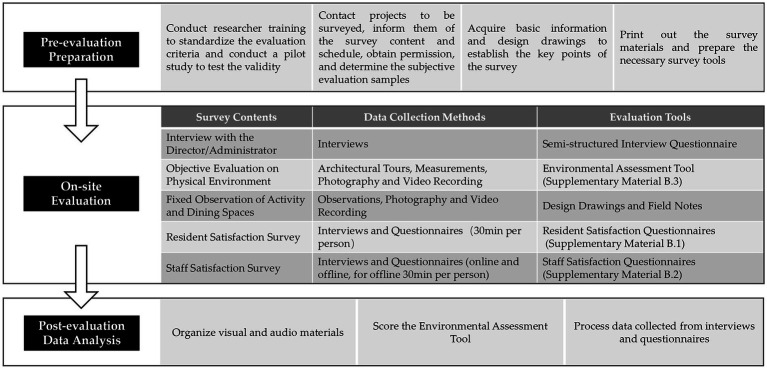
Evaluation process and content.

The collection of evaluation data was carried out by 4 authors from January to June 2021. The specific evaluation process is depicted in Figure 2. During the evaluation preparation phase, the evaluators underwent a brief training program to understand the meaning of the evaluation indicators, highlight the key points for data collection and to standardize the evaluation criteria. A pilot study at 10 projects was conducted to test the content validity and interrater reliability of the assessment tool, as well as the effectiveness of the satisfaction questionnaire. Before the formal survey, we contacted the project to obtain research permission and the necessary basic information. The on-site evaluation, depending on the scale of the project, typically lasted for 1 to 2 days. Observations and interviews with different users were comprehensively utilized to ensure more extensive and unbiased data, while also balancing and complementing opinions from researchers, residents, and staff. Based on data collected from field research, the environmental assessment tool was scored, and satisfaction survey questionnaires were analyzed.

It should be noted that, based on existing studies, the average age of residents in LTCFs is close to 85 years, and the caregivers are often between 50 and 59 years old (36, 51). Considering the limitations of respondents’ vision, comprehension, and endurance, the study mainly conducted interviews in a face-to-face way to collect more comprehensive and in-depth data. Some residents’ surveys were conducted with the assistance of their intimate staff. Additionally, an online survey with the same questionnaire was also conducted among younger staff to improve the research efficiency and increase the sample size. The study was approved by the Ethics Committee of Zhejiang University.

### 2.4 Evaluation data analysis

The data collected from both subjective and objective evaluations were divided into two types: quantitative data and qualitative data. Quantitative data comprised physical environment evaluation scores of 37 LTCFs and satisfaction scores from 195 users, while qualitative data mainly consisted of feedback provided by users through satisfaction surveys. A statistical analysis of quantitative data, including means and standard deviations, was conducted by SPSSAU and EXCEL to assess the overall environmental quality of the studied samples. Coding analysis of qualitative data was conducted using ATLAS.ti 9 and EXCEL to identify and summarize current issues and causes.

## 3 Results

### 3.1 Objective evaluation results

#### 3.1.1 Overall scores of different projects

**Figure 3:**
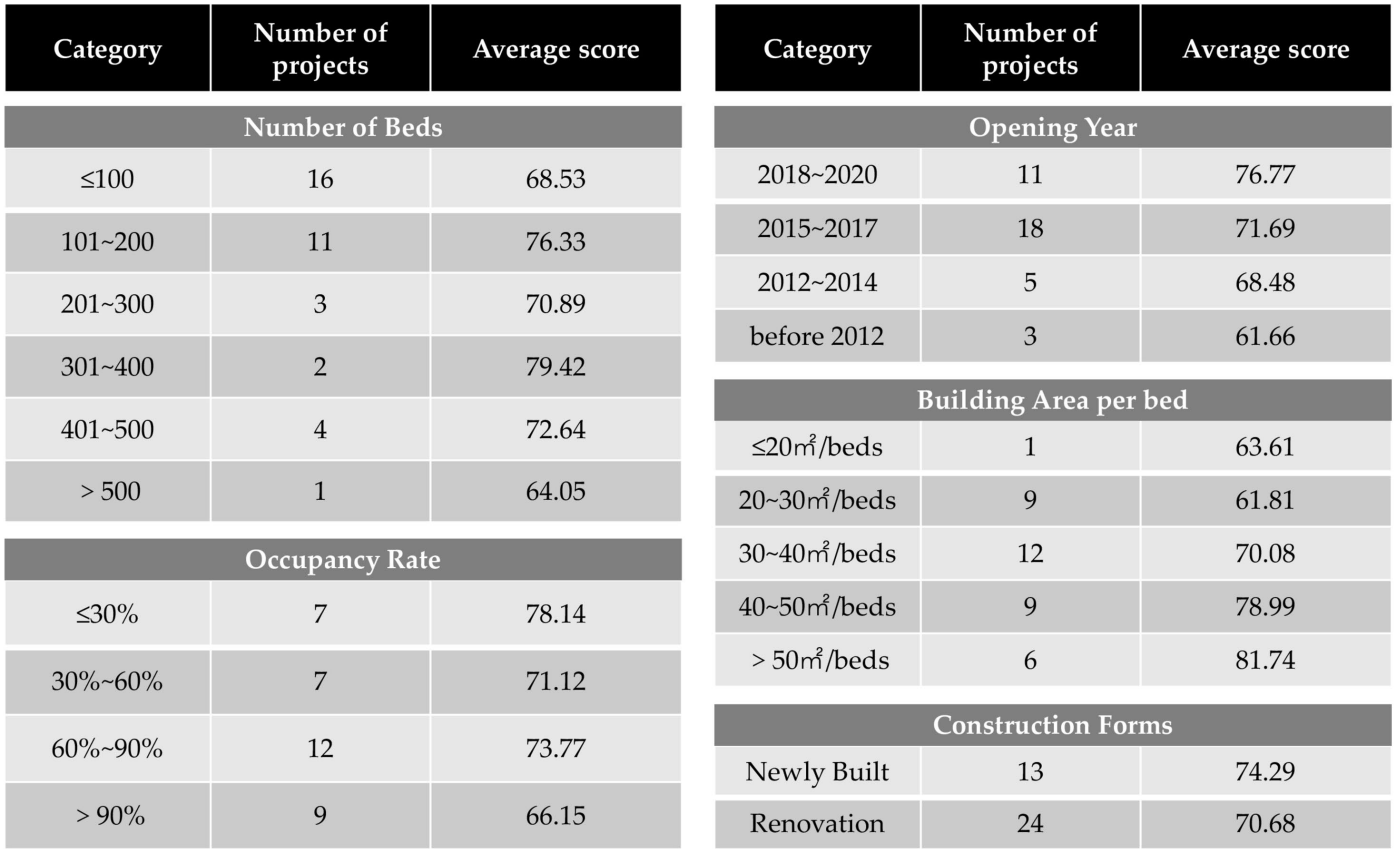
Objective evaluation scores of different types of projects.

The average score of the environmental assessment tool for 37 projects is 72.0. Among them, there are 10 projects with scores above 80.0, accounting for 27.0% of the total. 21 projects fall within the range of 60.0 to 80.0, accounting for 56.8%, and 6 projects with scores less than 60.0, accounting for 16.2%. The quality of environments varies across projects, with a minority demonstrating exceptional standards while the majority fall short in meeting user demands. An analysis of scores for different types of projects showed the following: (1) Newer projects scored higher, suggesting an improvement in construction quality over time. (2) Projects that were too large or too small scored lower. Large projects may suffer from overcrowding, while small ones might lack full functionality due to space constraints. Generally, there is a positive correlation between the building area per bed and the scores. (3) Occupancy rates also affected scores, with higher occupancy rates correlating to lower scores. When fewer residents are present, the space feels more accommodating. However, as the number of residents increases, the demands become more complex, and the contradictions between space and needs gradually emerge (Figure 3).

#### 3.1.2 Scores of different environmental design elements

**Figure 4:**
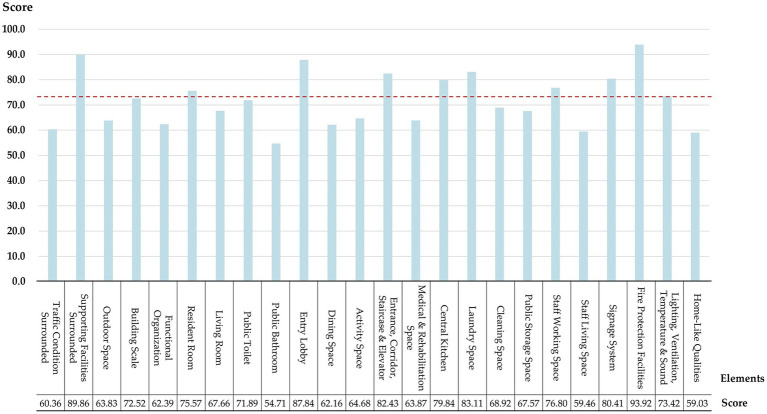
Objective evaluation scores of 24 environmental design elements.

To explore the deficiencies in specific environmental design elements of LTCFs, the average scores of 37 projects on 24 environmental design elements were calculated (Figure 4). The results indicate that Fire Protection Facilities (FPF) scored the highest at 93.9, while Public Bathroom (PB) scored the lowest at 54.7. Elements with scores above 80.0, show good quality, including Supporting Facilities Surrounded (SFS), Entry Lobby (EL), Entrance, Corridor, Staircase & Elevator (ECSE), etc. Elements with scores below 70.0, which account for half of total elements, show low quality and fail to adequately meet the diverse needs of both residents and staff, including Outdoor Space (OS), Functional Organization (FO), Public Bathroom (PB), Medical & Rehabilitation Space (MRS), Staff Living Space (SLS), Home-Like Qualities (HLQ), etc.

#### 3.1.3 Scores of different assessment indicators

To gain a more intuitive understanding of which assessment indicators are commonly not met by care facilities, a statistical analysis of the average scoring for 160 indicators was conducted. Tables 3, 4 present the distribution of scores as well as the content of the 10 lowest-scoring items. Nearly 30% of the indicators scored below 60.0. These items pertain to aspects such as the proportion of single rooms, various types of dining environments, hairdressing spaces, etc. This indicates that there are still deficiencies in LTCFs regarding the functional spaces in terms of spatial diversity and privacy.

**Table 3:** Distribution of objective evaluation scores of 160 assessment indicators.

Average score	0.00 ~ 20.00	20.00 ~ 40.00	40.00 ~ 60.00	60.00 ~ 80.00	80.00 ~ 100.00
Number and share of indicators	3 (1.87%)	9 (5.63%)	32 (20.00%)	48 (30.00%)	68 (42.50%)

**Table 4:** The 10 lowest-scoring assessment indicators.

No	Assessment indicators	Score	Design elements
20	The outdoor activity space is equipped with public toilet for residents nearby.	8.11	Outdoor Space, OS
4	The care facility has barrier-free motorized parking spaces, meeting the following conditions: The parking space is located near the main entrance of the building. Width of passageway along one side of the parking space ≥1.2 m, providing direct access to the sidewalk and main entrance. The parking space is clearly signed with parking lines, wheelchair access lines, and barrier-free signs painted on the ground.	11.43	Traffic Condition Surrounded, TCS
115	The care facility is equipped with a separate area for hospice services (e.g., hospice room, hospice area, etc.) near the pathway for human remains. The hospice area is strategically designed to avoid interference with surrounding environment.	18.92	Medical & Rehabilitation Space, MRS
30	Single rooms account for over 50% of all rooms.	21.62	Resident Room, RR
78	The dining space can meet the different dining needs of residents, meeting at least one of the following conditions: Equipped with private rooms or other separate dining areas for family gatherings. Equipped with various types of dining spaces such as cafeterias and food bars. Meets the needs of different dining preferences, including self-service, meal delivery assistance, and ordering options.	21.62	Dining Space, DS
31	The rooms for residents with dementia are single rooms.	25.00	Resident Room, RR
6	The care facility offers shaded, non-motorized parking.	25.71	Traffic Condition Surrounded, TCS
111	The care facility equipped with separated pathway for emergency medical evacuation and removal of mortal remains that do not go through resident areas.	29.73	Medical & Rehabilitation Space, MRS
77	The dining space offers different types of tables, such as two-person and four-person tables, catering to individual or group dining preferences for the residents.	30.56	Dining Space, DS
65	The care facility is equipped with a hairdressing and barbering room or space to meet the needs of residents.	33.33	Public Bathroom, PB

#### 3.1.4 Scores of different environmental design principles

To evaluate the degree of alignment between physical environments and environmental design principles in LTCFs, the average scores of 37 programs on 14 environmental design principles were calculated (Table 5). The results indicate that the physiological needs of residents have been met to a greater extent. *“Safety and Health”* and *“Physical Frailty Support”* have the highest scores above 80.0. However, the psychological needs of the older adult, such as *“Choice and Control”* and *“Cognitive Support,”* received scores below 60.0. Generally, enhancements could be made to the support of these resident’s psychological needs, as well as the staff’s needs, such as *“Service Support”* and *“Staff Facilities.”*

**Table 5:** Objective evaluation scores of 14 environmental design principles.

Environmental design principles	Number of related indicators	Average score	Environmental design overall principles	Average score
Safety and Health	26	84.7	Physiological Needs of Older Adult Residents	76.0
Physical Frailty Support	29	81.2		
Functional Support	34	72.2		
Accessibility	25	74.1		
Comfort	15	65.6		
Privacy	8	61.0	Psychological Needs of Older Adult Residents	65.2
Choice and Control	11	59.5		
Home likeness	20	62.2		
Social-Recreational Support	15	70.3		
Outdoor Freedom	6	68.5		
Cognitive Support	4	53.9		
Service Support	6	64.5	Staff Needs	70.6
Work Support	31	70.3		
Staff Facilities	4	64.2		

### 3.2 Subjective evaluation results

#### 3.2.1 Overall scores of different projects

Statistical analysis was conducted on the user satisfaction survey of 12 projects. The respondents’ average scores for “overall satisfaction” were calculated. The satisfaction levels of users in the 12 care facilities mostly range from “satisfied” to “very satisfied,” with the highest score recorded as 5.00 and the lowest as 3.98. This indicates a moderate level of user recognition toward the existing physical environment.

#### 3.2.2 Scores of different environmental design elements

**Figure 5:**
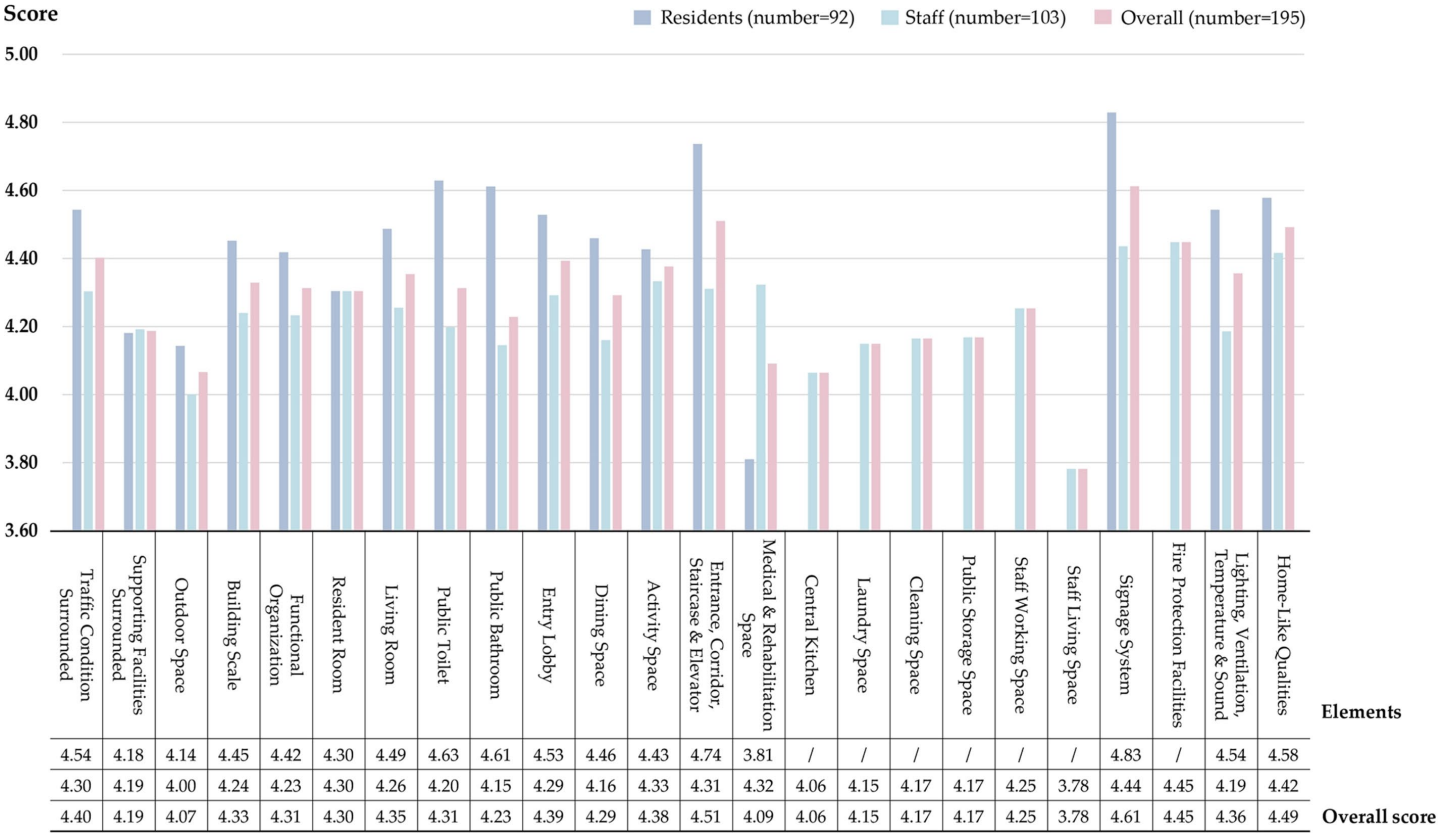
Subjective evaluation scores of 24 environmental design elements.

Since the given evaluation items in the satisfaction questionnaire for residents and staff were different, the average satisfaction scores of these two groups were separately analyzed (Figure 5). Overall, both groups exhibit higher satisfaction levels with Signage Systems (SS) and Traffic Spaces (TS), but lower satisfaction levels with Outdoor Spaces (OS) and Medical & Rehabilitation Spaces (MRS). The Medical & Rehabilitation Spaces (MRS) is the least satisfactory for residents, while the Staff Living Space (SLS) is the least satisfactory for staff. Significant differences in satisfaction levels are found between the two user groups regarding elements such as Public Toilet (PT), Public Bathroom (PB), Medical & Rehabilitation Spaces (MRS), and Lighting, Ventilation, Temperature & Sound (LVTS), indicating that different users have different perceptions of the same space. Additionally, the satisfaction level of residents generally exceeds that of the staff, implying that the staff holds higher expectations for physical environments.

#### 3.2.3 Analysis of qualitative data from subjective evaluations

To summarize the problems based on users’ perspectives, this study employed a data analysis method based on the Grounded Theory. Following Saldaña’s methods, two rounds of coding were conducted on textual data from satisfaction surveys provided by residents and staff (52). In the first round of coding, information pertaining to “problems” was identified and extracted based on different environmental design elements. These issues were marked with the prefix “#P” (Problem) and encoded in the format of “prefix + core content.” For example, one resident commented on his room as follows: *“The room has floor-to-ceiling glass windows, making it too sunny in the summer, while the operable window is not large enough, resulting in poor ventilation.”* Based on a basic analysis of this feedback, primary codes such as *“#P1_Excessive sun exposure in summer in the resident room”* and *“#P1_Poor ventilation in the resident room”* were obtained. Additionally, the frequency of each code’s appearance was calculated to reflect its universality. In the second round, all primary codes were summarized to identify common themes and format secondary codes labeled as “#P2,” which aimed to find out the main problems from a more general level. For instance, primary codes like *“#P1_Lack of separation between dry and wet areas in the resident room bathroom”* and *“#P1_Insufficient or inconvenient handrails in the resident room’s bathroom”* could be grouped under the secondary code *“#P2_Lack of safety and applicability in the resident room’s bathroom.”* Finally, 238 primary codes and 81 secondary codes were obtained, and the total frequency of all code’s appearance was 1,177 times.

**Figure 6:**
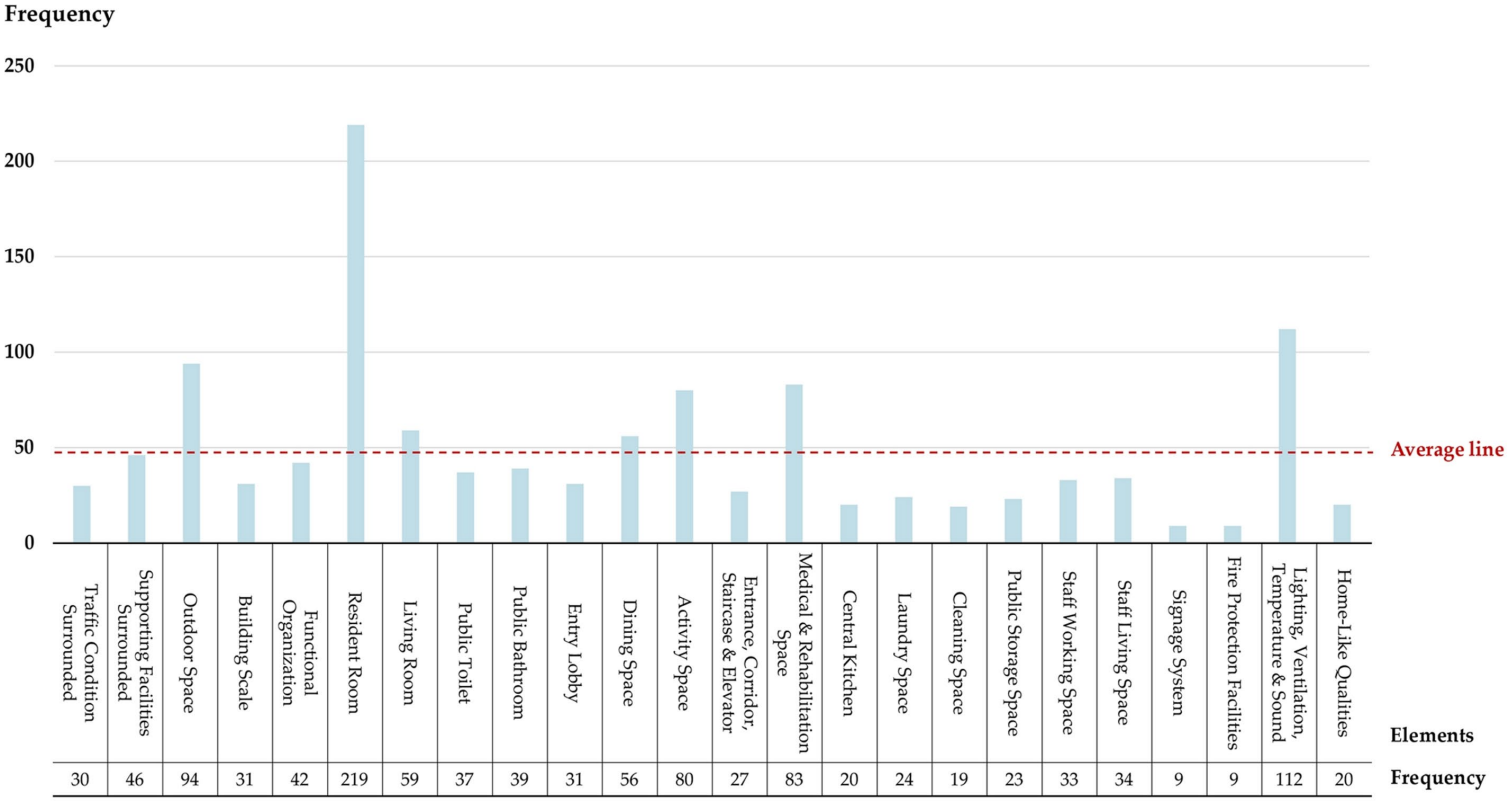
Frequency of #P (Problem) codes derived from users’ opinions regarding 24 environmental design elements.

The study counted the frequency of codes related to different design elements in order to ascertain the elements that exhibited a higher number of issues from the users’ perspectives (Figure 6). The results indicate that Resident Room (RR) has the most feedback problems, followed by Lighting, Ventilation, Temperature & Sound (LVTS), Outdoor Space (OS), and Medical & Rehabilitation Space (MRS). In terms of the specific content of the code, the most frequently mentioned by residents and staff are displayed in Table 6, which are the uppermost problems identified by users regarding the existing physical environment of LTCFs in China.

**Table 6:** The top_10 #P2 (Problem) codes most frequently mentioned by residents and staff (content in bold represents the common issues mentioned by two groups).

#P2 (Problem) codes mentioned by residents	Frequency	#P2 (Problem) codes mentioned by staff	Frequency
#P2_Inadequate functional space in the resident room	44	**#P2_Insufficient area in the activity spaces**	28
**#P2_Poor ventilation in the functional spaces**	37	#P2_Insufficient area and type in the staff working spaces	27
**#P2_Lack of safety and applicability in the resident room bathroom**	30	#P2_Insufficient area and type in the storage spaces	22
**#P2_Lack of essential supporting facilities in surrounding area**	29	**#P2_Insufficient area in the outdoor spaces**	21
**#P2_Inappropriate proportion of resident room types**	28	#P2_Inappropriate functional organization	19
**#P2_Insufficient area in the outdoor spaces**	25	#P2_Insufficient area and quantity of public toilets	19
**#P2_Insufficient area in the activity spaces**	22	#P2_Insufficient area in the cleaning spaces	18
#P2_Poor daylighting in the functional spaces	21	**#P2_Poor ventilation in the functional spaces**	17
#P2_Low utilization of medical rehabilitation spaces	20	**#P2_Lack of essential supporting facilities in surrounding area**	17
#P2_Lack of landscape design in the outdoor spaces	19	**#P2_Insufficient area in the resident rooms**	17

## 4 Discussion

### 4.1 Summary of current problems

Through quantitative and qualitative analysis of the POE data, and based on the #P2 codes with high frequency obtained from subjective evaluations, combined with assessment indicators that scored low in objective evaluations, a Physical Environmental Problem List of Long-term Care Facilities in China was constructed. The list includes 92 typical problems, categorized according to the 24 environmental design elements in the evaluation tool (Supplementary Material C). Disregarding the design elements to which the problems belong, a further cluster analysis of these 92 problems revealed that they can be categorized into eight distinct categories based on their attributes.

**Figure 7:**
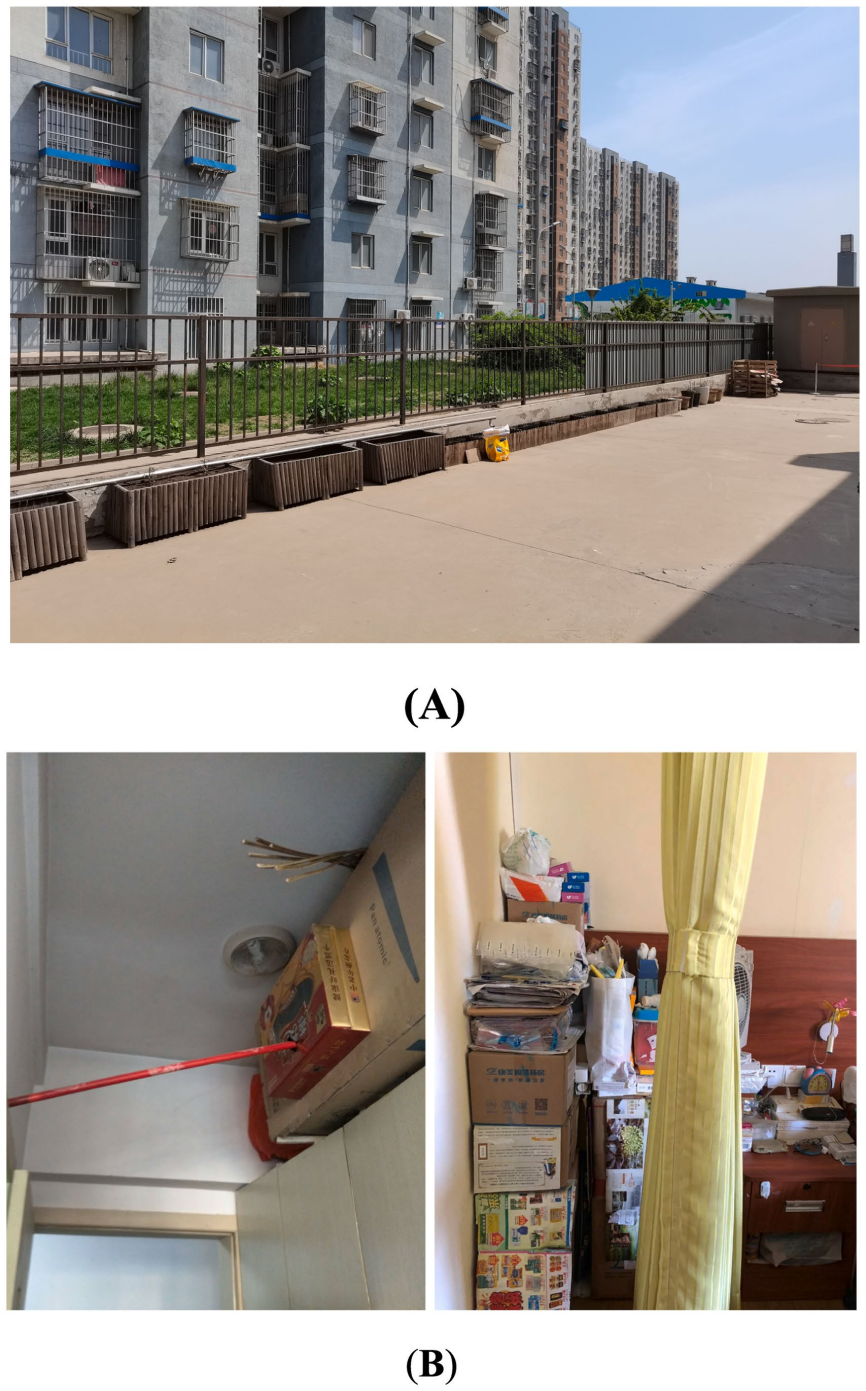
**(A)** The outdoor space is small with lack of necessary shading facilities and sports equipment, resulting in low utilization; **(B)** Insufficient storage space in resident room leaves no place to store residents’ belongings.

**Figure 8:**
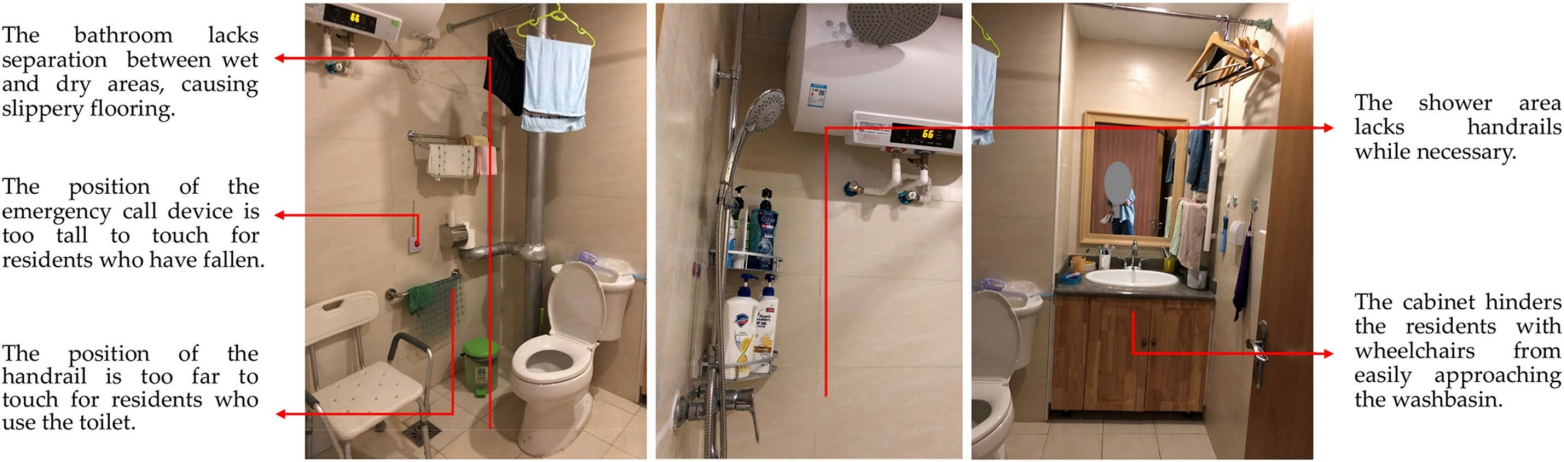
Examples of issues regarding barrier-free design and inconvenient equipment in the resident room bathroom.

**Figure 9:**
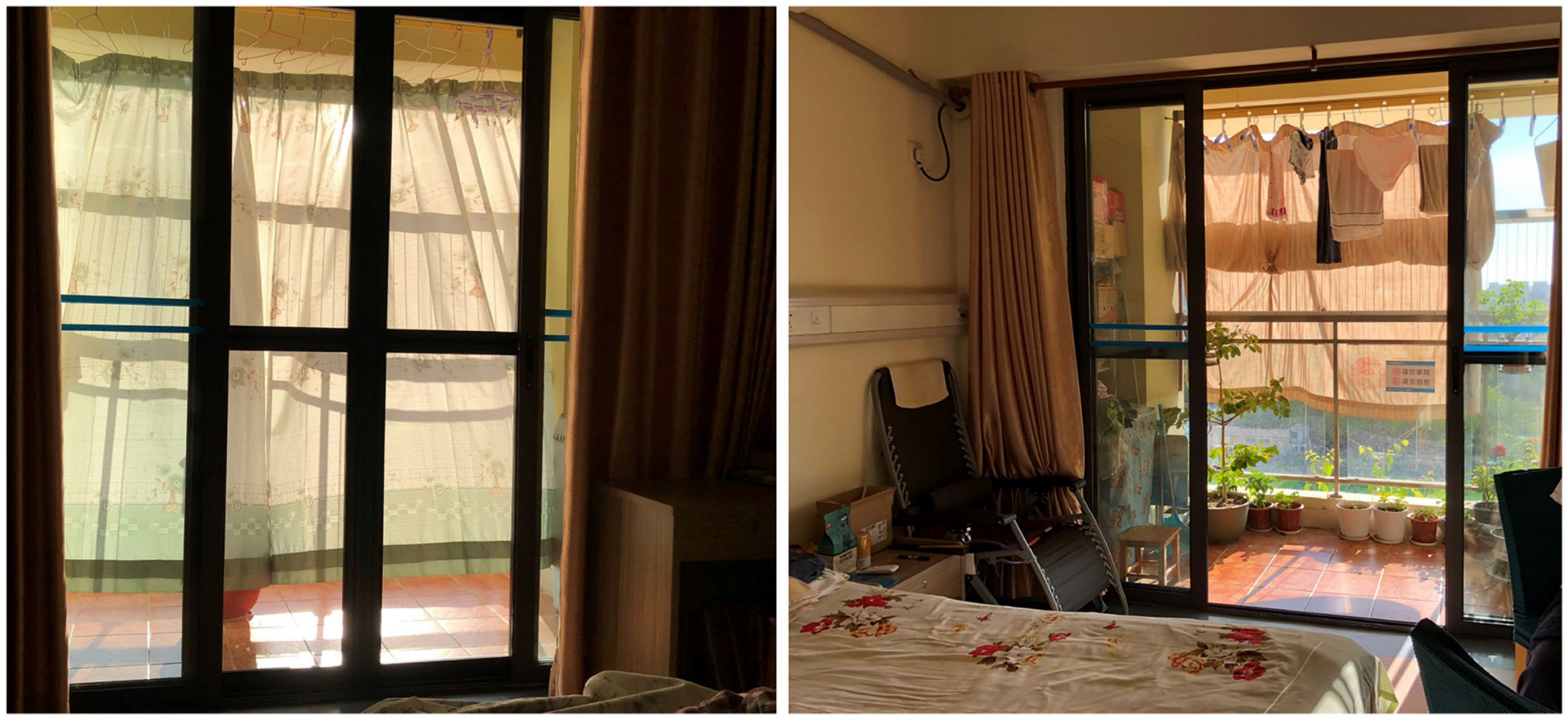
West-facing rooms have excessive sun exposure in summer, forcing older adult residents to buy curtains themselves for shading.

**Figure 10:**
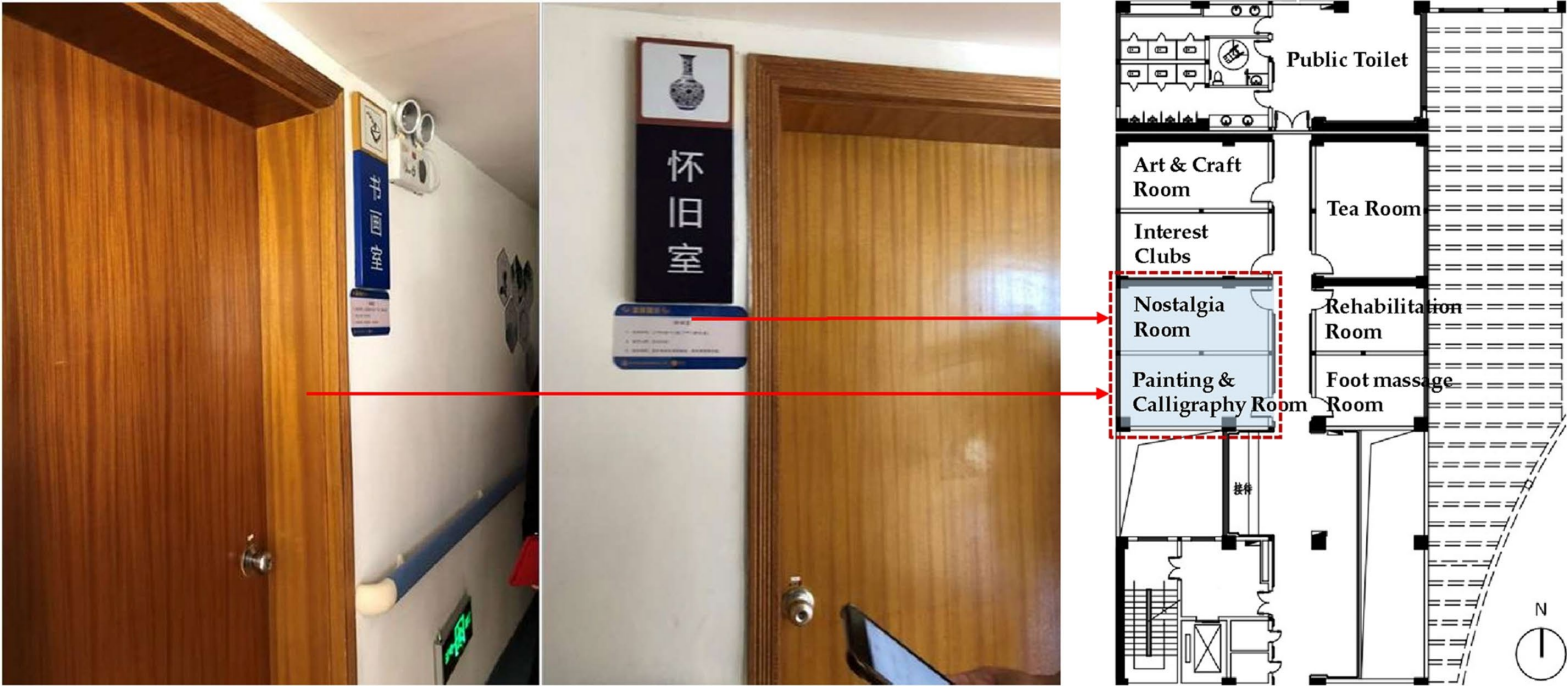
The activity spaces are enclosed rooms, which prevents flexible and multifunctional use.

1. Unreasonable Size, Scale or Quantity: The most fundamental issue identified in this study is the lack of rationality in the area, scale, and quantity of various functional spaces. Some care facilities have insufficient floor area *per capita* and fail to provide essential functional spaces such as outdoor activity areas, medical & rehabilitation spaces, and staff dormitories. As a result, the needs of residents and the demands of staff cannot be adequately supported. On the other hand, some projects have an excessive scale, resulting in inconveniences and negative user experiences. For instance, according to user feedback, the number of beds in a single care unit exceeds 60, which makes the staff feel it is difficult to take care of every resident and makes residents feel crowded and uncomfortable.
2. Insufficient Supporting Facilities: The problem mentioned above mainly concerns the size, scale, and quantity of functional spaces. However, even when certain spaces have adequate size or quantity, there still exists a phenomenon where they fail to meet user needs due to the insufficient supporting facilities. For instance, certain care facilities may possess outdoor areas, yet lack proper landscape design and functional configuration, thereby preventing the older adult from carrying out activities outdoors effectively (Figure 7A). Similarly, other examples include resident rooms lacking sufficient space for drying and storage (Figure 7B), public bathrooms lacking proper facilities for hair drying and clothes changing, and laundry rooms lacking space for folding and storage. The insufficient provision of supporting facilities for these functional spaces can significantly impact their efficacy and convenience in daily usage, potentially resulting in prolonged periods of non-utilization.
3. Poor Location or Layout: The problem of poor location or layout can be divided into three levels. Firstly, the site selection of the care facility is unreasonable as there are no supporting facilities available in the surrounding area, such as hospitals, parks, banks, supermarkets, and public transportation sites, leading to an isolated feeling for residents and inconvenient visits for family members. Secondly, the overall layout is illogical. For example, the living spaces for residents and the working spaces for staff are not separated, resulting in mutual interference between two groups in daily life. Thirdly, some functional spaces have inconvenient locations. A notable instance is the considerable distance between the central kitchen and the dining area, resulting in extended meal delivery and return routes, thereby imposing a greater burden on staff and exerting an influence on the quality of meals.
4. Failure to Meet Barrier-Free Requirements: The above POE results show that the physical environment of care facilities is mostly barrier-free, which is positive. However, it is also evident that there are still some issues that need to be addressed. For instance, outdoor areas, entrances, and other locations with height differences are not well designed, causing inconvenience to residents with wheelchairs or walking aids. Subtle changes in height or small steps can be difficult for the older adult to notice, leading to falls and other safety hazards. In addition, in toilets and bathrooms, where residents are most likely to fall, there are many problems causing hidden dangers, including the lack of handrails, the lack of separation between wet and dry areas, slippery flooring, poor drainage, etc.
5. Inconvenient Furniture, Equipment, or General Details: Inconvenient furniture, equipment, and design details are mainly found in resident rooms, toilets, and public bathrooms. For instance, in the resident rooms, poorly designed closets and shoe cabinets result in insufficient storage space, the unreasonable location and form of switches and sockets create safety hazards for the older adult when using electricity, and owing to the lack of door locks, night lights cannot ensure security and privacy. In terms of toilets, problems include inconvenient handrails, inconvenient location of the toilet roll holder, and an inaccessible washbasin for residents with wheelchairs (Figure 8). Similar problems are also observed in public bathrooms.
6. Unpleasant Physical and Visual Environments: This POE study reveals that users find the physical environment unpleasant due to factors such as sound, light, temperature, and an un-home-like visual environment. Daylighting and ventilation conditions in functional spaces are of particular concern to users. Certain issues exhibit a higher frequency of occurrence, encompassing insufficient sunlight in north-facing rooms, excessive summer sun exposure in west-facing ones (Figure 9), poor lighting and ventilation in double-loaded corridors, unpleasant odors in public bathrooms, damp and hot conditions in laundry rooms, etc. Regarding the visual environment, objective evaluations reveal that the public dining spaces, medical & rehabilitation spaces, and care units are severely lacking in home-like qualities. This sentiment is further confirmed in subjective evaluations, wherein many respondents feel that the current environment is *“like a hotel or a dormitory,”* rather than *“a home-like environment”* they expect.
7. Inappropriate Spatial Type or Form: The illogical of spatial design primarily manifests in three aspects, namely the proportion of types of resident rooms, the type and form of activity spaces, and the type of dining spaces. For room types, the contradiction between the high demand for single rooms (of 195 respondents, 67.3% prefer a single room) and their low proportion (of 37 LTCF samples, the average proportion of single rooms is 22.7%) is currently the main issue. However, staff members also express concerns about the insufficient number of multi-bed rooms for residents with severe disabilities. In terms of activity spaces, certain care facilities face limitations in providing a diverse range of spaces due to spatial constraints, which hinders the support for residents’ varied activity preferences. Additionally, some activity spaces are designed as enclosed rooms, hindering flexible and multifunctional use (Figure 10). Regarding dining spaces, many care facilities lack the provision of private dining rooms, cafes, or self-service restaurants, and instead rely on either a central dining hall or floor-based unit dining areas. This limited variety fails to cater to the diverse dining needs of residents.
8. Low Utilization of Functional Spaces: During the occupancy phase, certain functional spaces are often underutilized, resulting in a waste of space resources. This problem is particularly evident in medical spaces and public bathrooms, where there is a mismatch between the space pattern and caregiving mode. Moreover, the original functions of these spaces are excessively specific, rendering them resistant to adaptation for alternative purposes. As a result, operators often choose to maintain their current status or convert them into spaces with lower functional requirements, such as staff changing rooms, drying areas, or storage rooms. The utilization of these spaces is thus suboptimal.

### 4.2 Analysis of the causes

The aforementioned issues can primarily be attributed to the incongruity between the physical environment and user requirements. Therefore, what are the underlying causes of this incongruity? Based on comprehensive evaluation data from this POE study and other relevant studies, this paper analyzed the causes of these issues by adopting a comprehensive life cycle perspective of the LTCFs. In summary, they involve the four aspects listed below.

#### 4.2.1 Programming and site selection phase

Due to the lack of scientific methods and maturity of experience in LTCF programming in China (28), many projects do not conduct adequate research on target users and their surrounding environment during the initial planning phase. This leads to a lack of basis for design decisions regarding supporting spaces, room types, interior decoration styles, etc. Consequently, the built environment may not align with user needs, causing issues such as *“inappropriate spatial type or form”* and *“low utilization of functional spaces.”* LTCFs located in the core areas of cities are mostly renovation buildings. Problems like *“unreasonable size, scale or quantity”* and *“poor location or layout”* may often occur because of restrictions on size, structure, and layout of the original buildings. However, for newly built projects in suburban areas, the lack of adequate supporting resources such as public transportation and medical facilities may hinder the fulfillment of older adult individuals’ requirements for outdoor activities and access to healthcare services.

#### 4.2.2 Planning and design phase

The design requirements of LTCFs are intricate, posing significant challenges for architects in creating spaces that cater to diverse needs (44). However, in reality, many architects are middle-aged and lack an understanding of the older adult’s characteristics and behavioral patterns, and usually pay less attention to staff needs (53). Consequently, they may fail to accurately comprehend the physical and psychological needs of both older adult residents and staff members, giving rise to issues such as *“insufficient supporting facilities,” “the failure to meet barrier-free requirements,”* and *“inconvenient furniture, equipment, or general details.”* Furthermore, the current design standards are not fully comprehensive and are often short on the necessary detail, leaving architects without clear directives (54). As a result, some may turn to standards for residential, hospital, or hotel projects without fully comprehending the authentic requirements, or mimic international cases without considering cultural contexts. This can lead to disparities between conceived spatial designs and actual demands (55). Moreover, in the current stage of development in China, there is a clear separation between the design work and operation of care facilities (53). Architects often lack the opportunity to obtain correct and sufficient information from clients regarding the needs of their staff, their preferred methods of care delivery, or the anticipated needs of the residents, which ultimately results in a mismatch between the built environment and operational requirements.

#### 4.2.3 Construction phase

Many of the above issues related to the *“failure to meet barrier-free requirements”* and *“inconvenient furniture, equipment, or general details”* are caused by substandard construction quality. Improper drain design, for instance, can result in poor bathroom drainage, leading to the accumulation of water on the floor, thus posing a potential slip hazard for occupants. Additionally, insufficiencies in safety and suitability considerations have led to numerous challenges pertaining to material, equipment, and furniture selection. For example, certain care facilities may opt for marble flooring in their lobbies with the intention of creating an opulent ambiance; however, this choice can potentially jeopardize resident safety if appropriate anti-slip and anti-glare measures are not implemented.

#### 4.2.4 Operation phase

During the operation of LTCFs, changes in caregiving modes and resident demands may arise due to operator replacements, staff organization adjustments, and variations in residents’ physical conditions, consequently necessitating further adaptations in physical environmental requirements (56). A lack of flexibility in the environment may gradually render it inadequate in meeting the evolving needs of both the older adult and the staff. For example, in Project #03 of this study, the facility initially accommodated a small number of severely disabled residents and featured only one public bathroom designed for individuals requiring assistance with bathing beds. However, after 2 years, there was an increase in the number of residents necessitating bathing beds, resulting in the public bathroom being insufficient. Consequently, certain bedridden residents were constrained to taking sponge baths, leading to a decline in service quality.

Some of the causes mentioned above are universal factors occurring in all types of buildings in China, including unscientific programming methods and lower construction quality. These problems need to be addressed by improving the overall level of engineering construction. However, more causes are related to the particularity of LTCFs. The two primary user groups of this particular project exhibit distinct, dynamic needs that pose intricate challenges to address. Architects are expected to devise suitable and adaptable spatial solutions in response to these requirements, necessitating a profound comprehension of diverse needs and their evolving nature. Nevertheless, it is important to acknowledge that improving the quality of the physical environment is a gradual process. Understanding the specific design requirements for a particular building type is a step-by-step process which requires continuous POE studies.

### 4.3 Recommendations for future design practices and revision of design guidelines

The analysis of the issues and their causes presented above can provide some insights for future design practices and the revision of related standards in China (especially the Standard for Design of Care Facilities for the Older Adults) (40). This mainly includes the following aspects.

#### 4.3.1 Emphasis on refined needs of functional space

This study reveals that the Outdoor Spaces, Resident Rooms, Public Bathroom, and Staff Living Space elements scored low in objective evaluations and were identified with relatively more issues in subjective evaluations. Although the current standards have set basic requirements for these spaces, there is insufficient attention to the detailed needs frequently highlighted by respondents, such as the older adult’s need for outdoor activities like sunbathing, cooling off, walking, and exercising; the demand for bedrooms with an en-suite bathroom; the need for recumbent bathing facilities for the older adult with disabilities; and the staff’s need for living and resting spaces. These concerns should be taken seriously in future design practices, and corresponding design guidance perhaps should also be added to the standards.

#### 4.3.2 Attention to the psychological needs of the older adult

Objective evaluations of environmental design principles show that scores for the psychological needs of residents are low, particularly regarding Privacy and Choice and Control, indicating a lack of adequate spatial response. For instance, this POE found that some LTCFs only offer single or double rooms, with little variety in room types. However, based on subjective feedback, the older adult’s preferences for room types vary. Some residents, based on marital or partnership status, prefer not to stay in single rooms, while those with higher privacy demands do not wish to stay in double rooms. They believe that the LTCF should offer a variety of room types to cater to different preferences. Addressing the older adult’s psychological needs in the physical environment should be a priority in future design practices and standard revision.

#### 4.3.3 Attention to the caregiving service needs of the staff

This study has gathered feedback on the current state of the physical environment from the perspective of staff members, highlighting typical issues such as the large capacity of care units, the distant location of dining and kitchen areas, and the lengthy workflow paths for laundry and drying, which contribute to increased workload and reduced service efficiency for caregivers. As the aging population deepens, China is facing a growing caregiving burden coupled with a shortage of care staff. Therefore, paying attention to the needs of staff when providing services, and optimizing spatial layout and design to enhance service quality and efficiency, are also key areas that require focus in the future.

#### 4.3.4 Emphasis on flexibility and target-oriented direction

Many of the guidelines in current standards in China are prescriptive, providing quantitative or qualitative requirements without adequate explanation (57). This POE study found that some spaces that do not conform to standard design requirements can still meet user needs and receive positive feedback. For example, while standards mandate the provision of spaces for reading, internet access, and board games, in practice, multifunctional spaces without predefined uses, accommodating various scales and levels of privacy, have shown greater flexibility. This suggests that, when formulating standards, we should focus more on performance and functional requirements, providing flexible rather than rigid regulations. It is also essential to provide instructions to help users understand the underlying purposes and principles.

#### 4.3.5 Emphasis on evidence-based design and research

Current standards in China rely heavily on expert experience and lack empirical support, which can lead to discrepancies with actual needs (54). For example, standards suggest an upper limit of 60 beds for general care units and 20 beds for dementia care units. However, empirical research based on this POE indicates that the older adult and staff prefer smaller unit sizes, recommending 29 beds and 14 beds, respectively. Additionally, there is a discrepancy between the standard prescribed maximum number of beds in a multiple room (6 beds) and the number preferred by users (4 beds) (58). This highlights the need for more evidence-based research to guide design practices and the development of standards.

## 5 Conclusion

The construction of LTCFs in developing countries is currently undergoing a transition from prioritizing quantity to emphasizing quality (59). Therefore, conducting a systematic POE analysis of LTCFs to identify key issues and potential causes holds significant importance. Taking China as a case study, this research employed a self-developed assessment tool to objectively evaluate the physical environment of typical LTCFs nationwide, while also conducting interviews with diverse users for subjective evaluations. The primary strength of the study is the combination of subjective and objective measures of POE for care facilities and the is first study of its kind in China. The Physical Environmental Problem List constructed in this study serves as an urgent call for immediate attention, while the proposed causes and recommendations can provide valuable insights for optimization.

There are also some limitations in this study, primarily including the following three aspects: (1) The data collection methods used in this study encompassed observation, interviews, and questionnaires. However, the latter two methods were only applicable to residents with communication skills, excluding those without, especially those with cognitive impairments. This limitation prevented this study from comprehensively gathering the perspectives of all types of older adult individuals. In the future, other data collection techniques including indoor positioning technology and video technology will be integrated to obtain more comprehensive and systematic data (60, 61). (2) Considering that LTCFs with different opening times, construction forms, and project scales may have varying spatial qualities and usage conditions, in order to obtain data that can fully reflect the “average construction level” of LTCFs in China and analyze common issues, this study took into account the differences in these factors when collecting samples. However, due to the limited number of samples obtained for each type of project, this study did not specifically focus on the characteristics and issues concerning particular types of LTCFs, such as those that are renovated from old buildings or small facilities with fewer than 100 beds. In-depth research on specific types of projects and comparative studies on the similarities and differences between different types of projects are areas that future research can continue to explore and deepen. (3) In contrast to nursing homes in developed countries that focus on the severely disabled and those with dementia (62, 63), China’s LTCFs currently cater to a more diverse and complex population, including self-sufficient older adult and those with varying levels of disability (43, 44). Therefore, the focus of this study is predominantly on these comprehensive facilities, and does not include specialized institutions that exclusively serve those requiring dementia care or palliative care. Mirroring trends in Europe and America, China’s LTCFs will also increasingly focus their services, particularly on individuals requiring intensive and cognitive care. Research into environmental support for specific types of older adult individuals, such as those with dementia or visual impairments will also be a priority in the future.

Furthermore, environmental requirements for LTCFs may vary over time among older adult individuals. Therefore, evaluation studies should be refined and adapted to establish an interconnected and continuously updated “evaluation-design” feedback loop for promoting the comprehensive and sustainable development of LTCFs, and thus improving life quality for the aged.

## Data Availability

The original contributions presented in the study are included in the article/Supplementary material, further inquiries can be directed to the corresponding author.
