# Supplementary material for: Improving life quality for the aged: a comprehensive post-occupancy evaluation of long-term care facilities in China
Source: Front Public Health. 2024 Nov 28;12:1488653. doi: 10.3389/fpubh.2024.1488653 (PMC11636290; doi:10.3389/fpubh.2024.1488653)
Supplement: Supplementary file 1 [file Data_Sheet_1.pdf]

Supplementary Material A

A.1 Features of POE samples in this study

Project No.[1]	Location	Opening Year	Project Size			Occupancy Rate[3]	Construction Forms
			Building Area[2] m[2]	Number of Beds	Building Area per Bed		
**01**	Beijing	2013	8000	219	36.53	30.1%	Renovation
**02**	Beijing	2015	14200	270	52.59	18.5%	Renovation
**03**	Beijing	2017	20881	480	43.50	41.0%	Renovation
**04**	Beijing	2016	14000	445	31.46	38.2%	Newly Built
**05**	Beijing	2018	5865	201	29.18	26.9%	Newly Built
**06**	Beijing	2012	1650	68	24.26	97.1%	Renovation
**07***	Beijing	2019	4500	75	60.00	76.0%	Renovation
**08**	Beijing	2012	7100	157	45.22	81.5%	Renovation
**09***	Beijing	2017	9246	190	48.66	24.7%	Newly Built
**10**	Beijing	2016	1278	28	45.64	Unknown	Renovation
**11**	Chongqing	2018	8215	155	53.00	80.0%	Newly Built
**12**	Foshan	2000	2366	98	24.14	49.0%	Renovation
**13**	Foshan	1998	5034	174	28.93	95.4%	Newly Built
**14**	Foshan	2014	5400	130	41.54	84.6%	Renovation
**15**	Foshan	2017	2000	94	21.28	100.0%	Renovation
**16***	Shanghai	2012	14000	445	31.46	96.6%	Renovation
**17**	Chengdu	2017	3000	71	42.25	50.7%	Renovation
**18***	Hangzhou	2020	13600	123	110.57	11.4%	Newly Built
**19**	Beijing	2017	2300	50	46.00	40.0%	Renovation
**20***	Beijing	2018	1873	47	39.85	95.7%	Renovation
**21**	Beijing	2018	3518	82	42.90	92.7%	Renovation
**22**	Beijing	2015	759	28	27.10	75.0%	Renovation
**23**	Guangzhou	2017	1250	38	32.89	73.7%	Renovation
**24***	Guangzhou	2015	1253	48	26.10	100.0%	Renovation
**25**	Shenzhen	2015	1010	60	16.83	100.0%	Renovation
**26***	Nanjing	2017	2247	83	27.07	83.1%	Renovation
**27**	Nanjing	2018	6588	164	40.17	82.3%	Renovation
**28**	Nanjing	2016	1650	48	34.38	85.4%	Renovation
**29***	Shanghai	2020	2800	85	32.94	23.5%	Newly Built
**30**	Chengdu	2016	3588	110	32.62	81.8%	Renovation
**31**	Guangzhou	2010	6527	189	34.53	Unknown	Newly Built
**32***	Shenzhen	2015	31469	791	39.78	44.9%	Newly Built
**33**	Nanjing	2019	16316	316	51.63	24.7%	Newly Built
**34***	Nanjing	2019	14000	406	34.48	72.7%	Renovation
**35***	Hangzhou	2017	22000	363	60.61	73.0%	Newly Built
**36***	Hangzhou	2018	3209	140	22.92	57.1%	Newly Built
**37**	Hangzhou	2015	4738	122	38.84	99.2%	Newly Built

[1] The projects marked with an asterisk * underwent both subjective and objective evaluations.
[2] The building area was calculated above-ground area only.

[3] The data was collected during the course of this study.

A.2 Detailed information, drawings, and photos of 37 samples in this study

Project 01

Location	Opening Year	Building Area	Number of Beds	Building Area per bed	Building Stories	Occupancy Rate	Types of Residents	Number of Staff	Construction Forms
Beijing	2013	8000㎡	219	36.53 ㎡/beds	Ground 4	30.1%	Independent; Functional loss; Dementia	27	Renovation

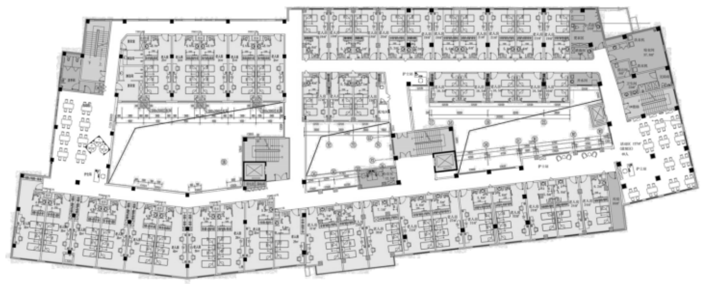

Standard Floor Plan

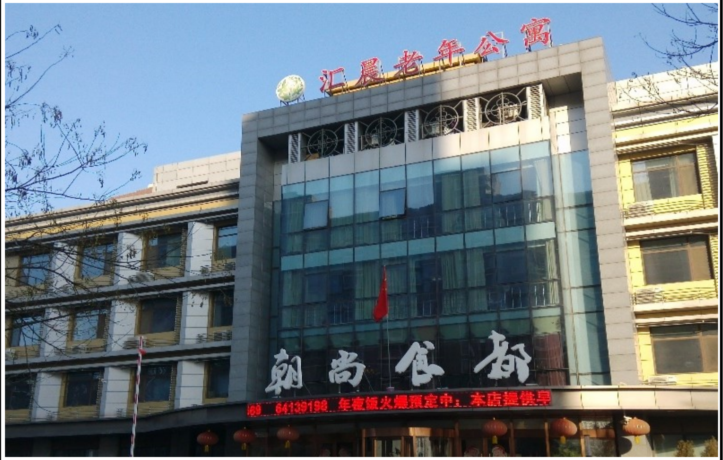

Building Exterior

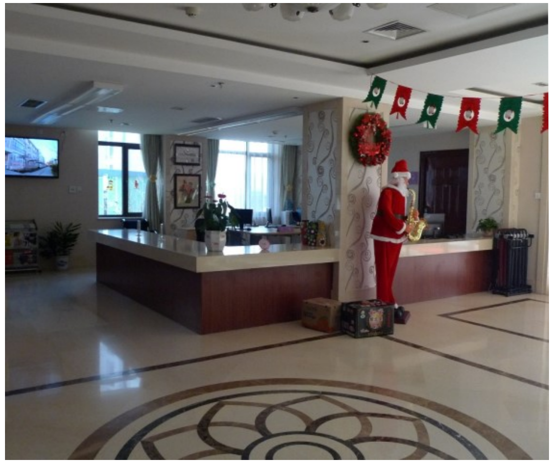

Entry Lobby

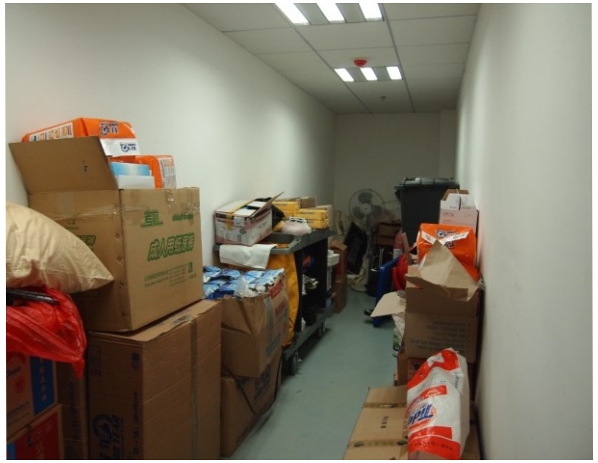

Public Storage Space

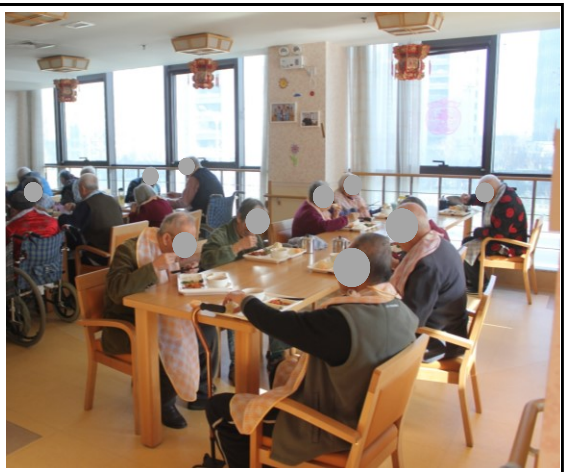

Dining Space

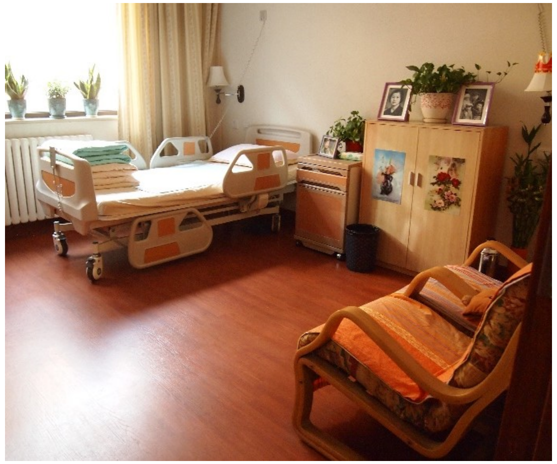

Resident Room

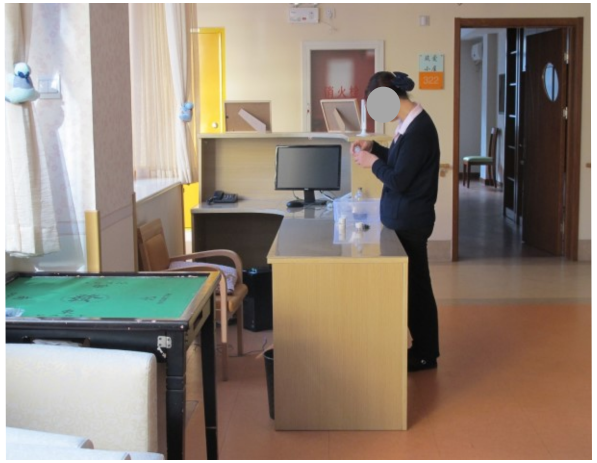

Nursing Station

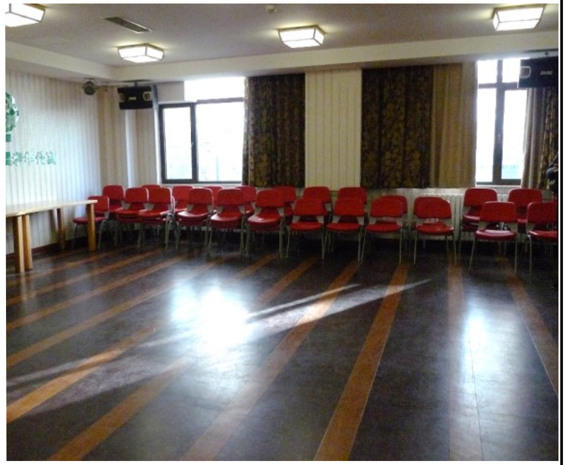

Multi-function Hall

Project 02

Location	Opening Year	Building Area	Number of Beds	Building Area per bed	Building Stories	Occupancy Rate	Types of Residents	Number of Staff	Construction Forms
Beijing	2015	14200m^2	270	52.59 m^2/beds	Ground 4	18.5%	Independent; Functional loss	68	Renovation

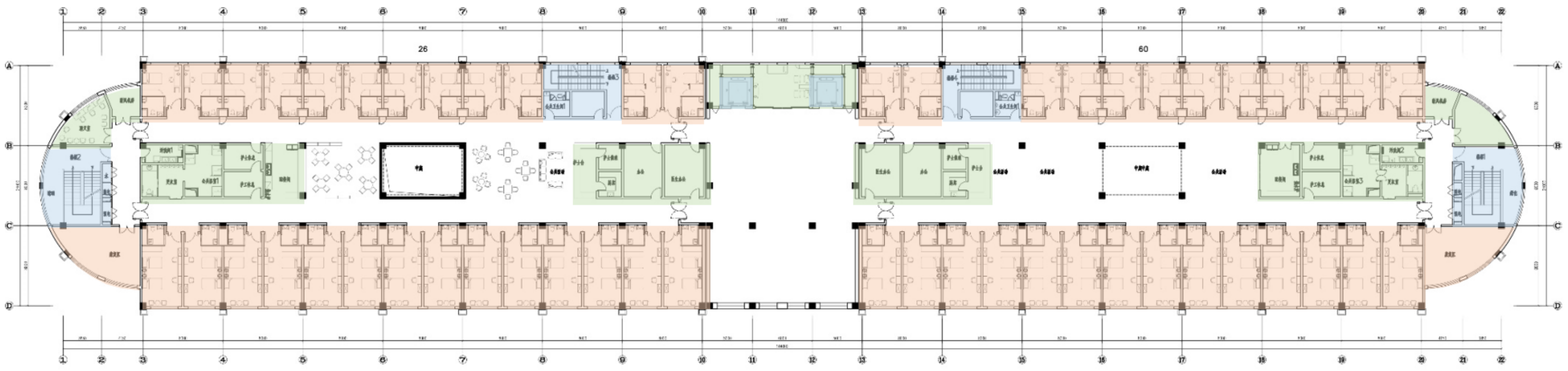

Standard Floor Plan

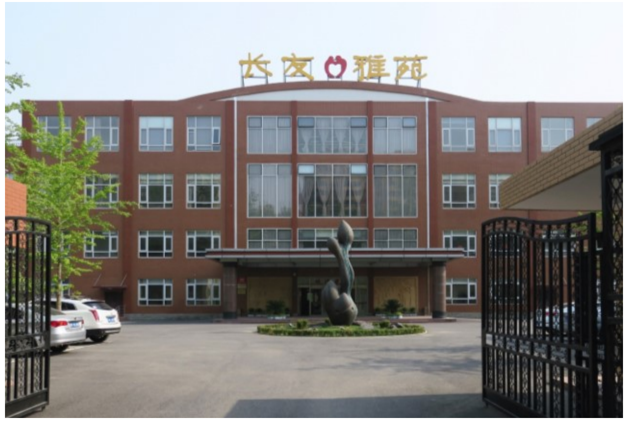

Building Exterior

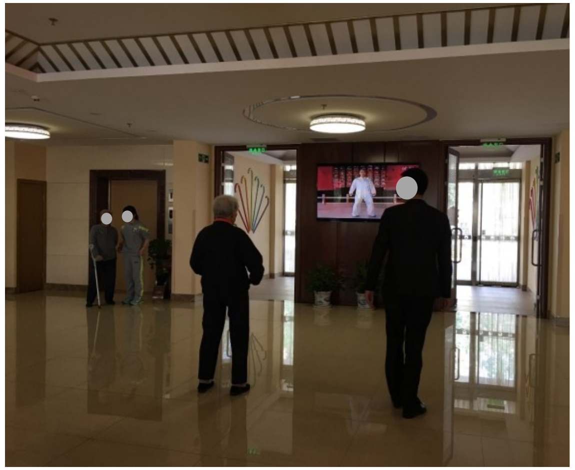

Entry Lobby

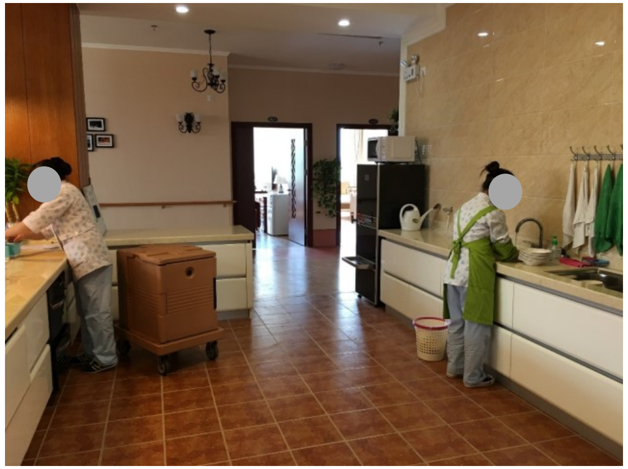

Dining Room

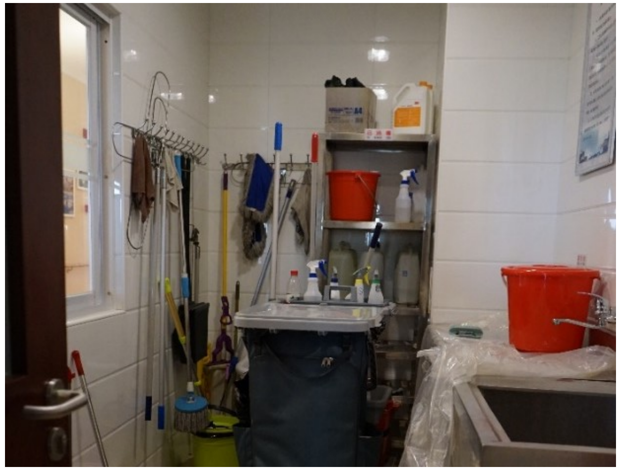

Public Storage Space

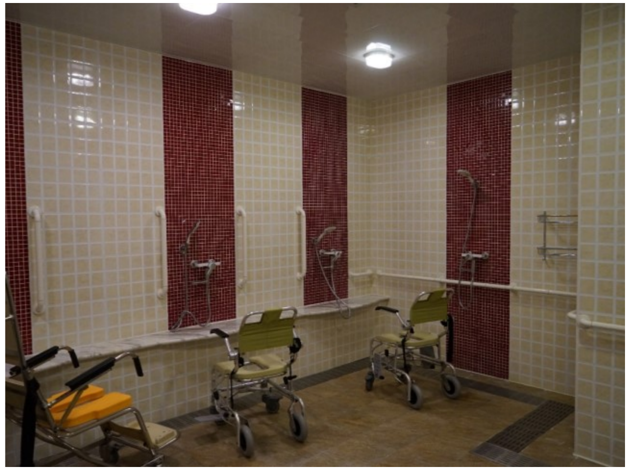

Public Bathroom

Project 03

Location	Opening Year	Building Area	Number of Beds	Building Area per bed	Building Stories	Occupancy Rate	Types of Residents	Number of Staff	Construction Forms
Beijing	2017	20881㎡	480	43.50 ㎡/beds	Ground 10 Underground 2	41.0%	Independent; Functional loss; Dementia	109	Renovation

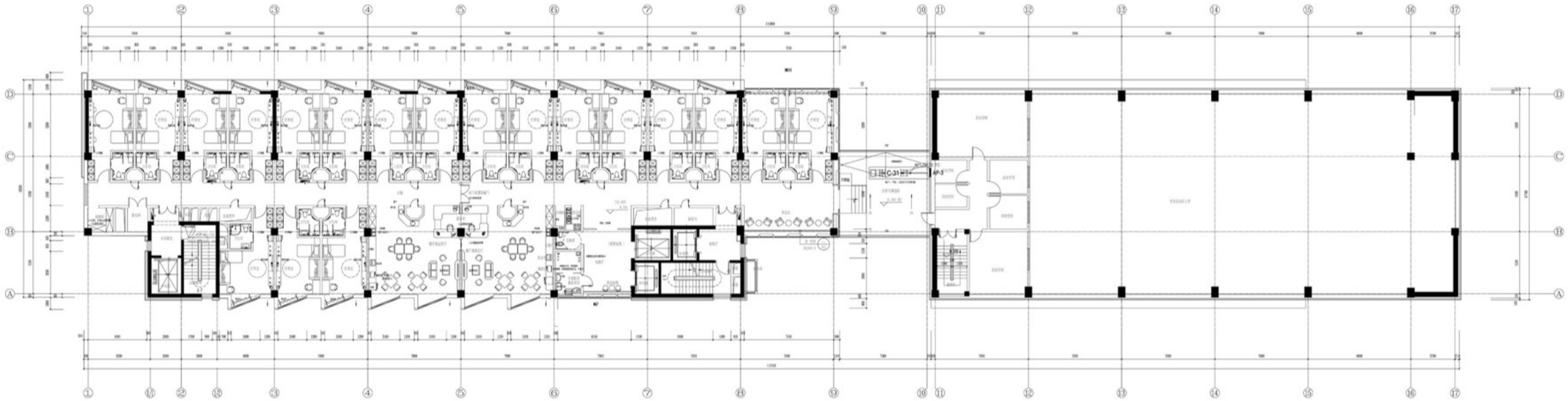
Standard Floor Plan

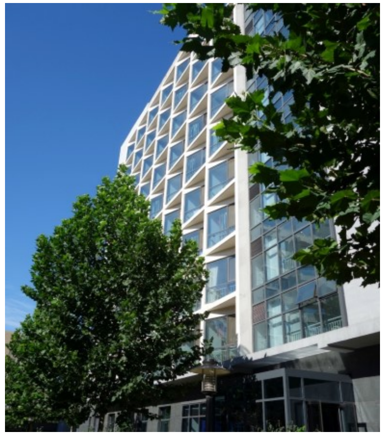
Building Exterior

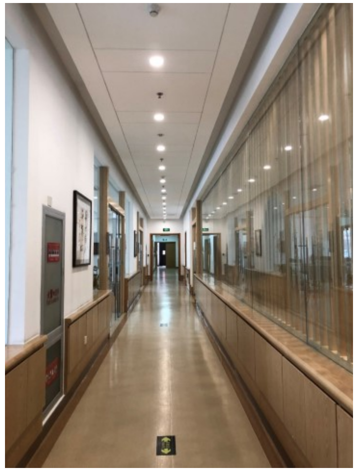
Corridor

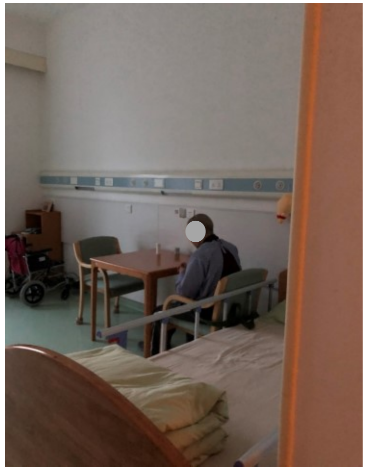
Resident Room

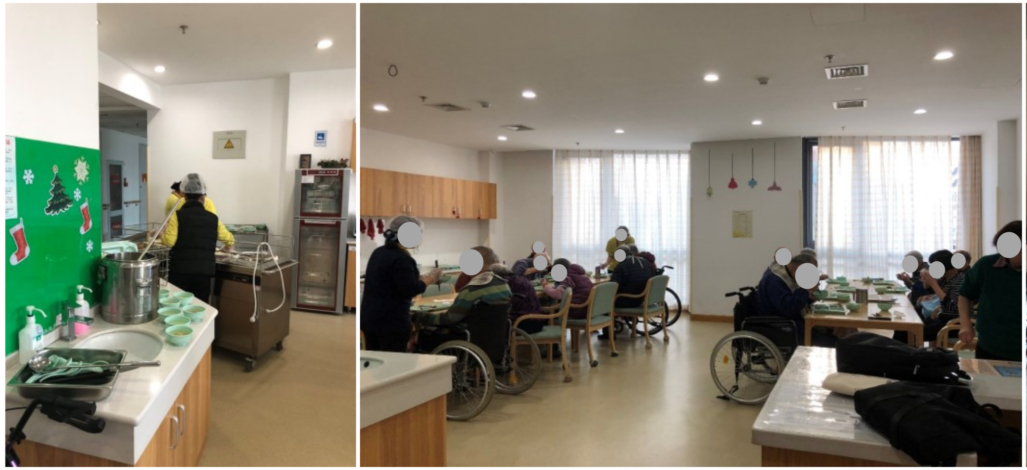
Dining Space

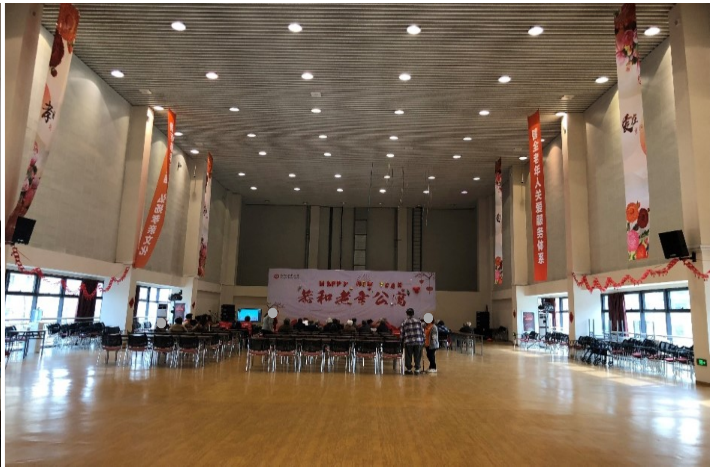
Multi-function Hall

Project 04

Location	Opening Year	Building Area	Number of Beds	Building Area per bed	Building Stories	Occupancy Rate	Types of Residents	Number of Staff	Construction Forms
Beijing	2016	14000 m²	445	31.46 m²/beds	Ground 3 Underground 1	38.2%	Independent; Functional loss	80	Newly Built

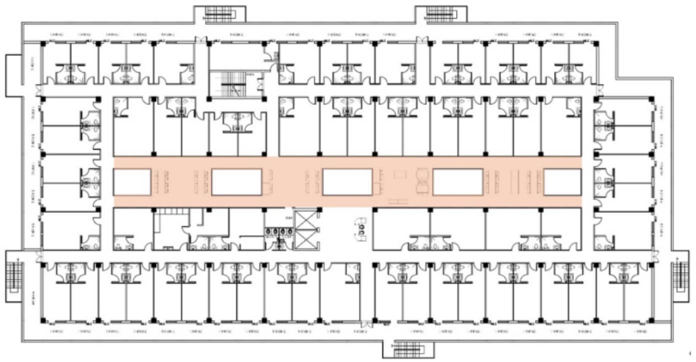

Standard Floor Plan

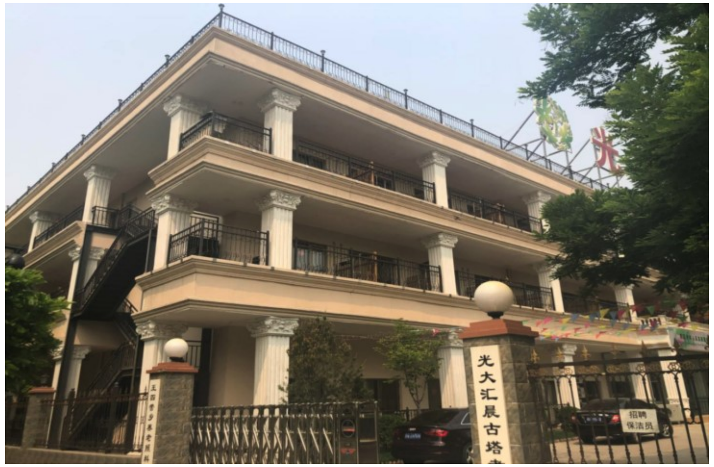

Building Exterior

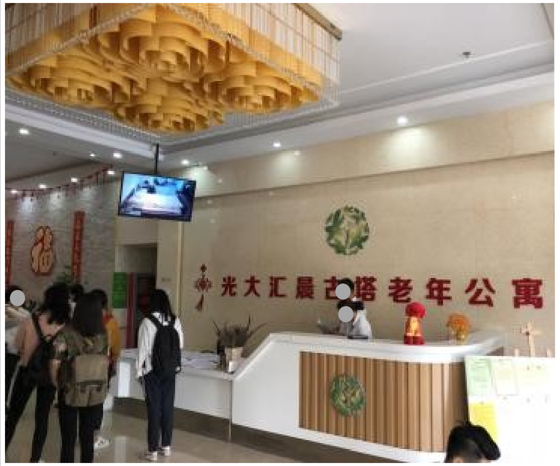

Entry Lobby

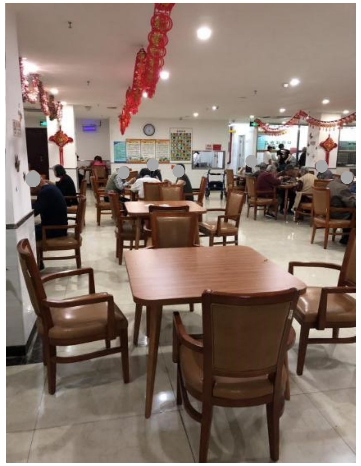

Dining Space

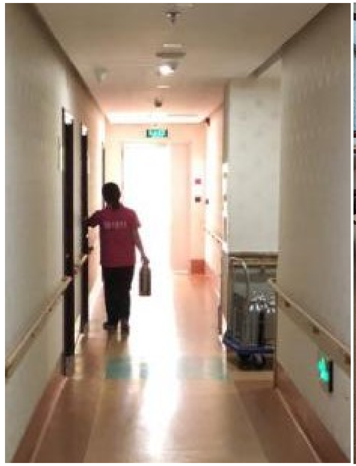

Corridor

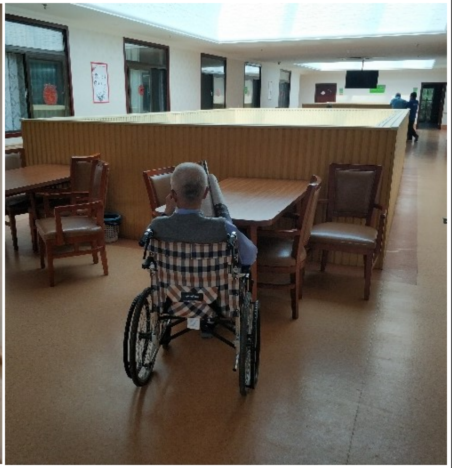

Activity Space

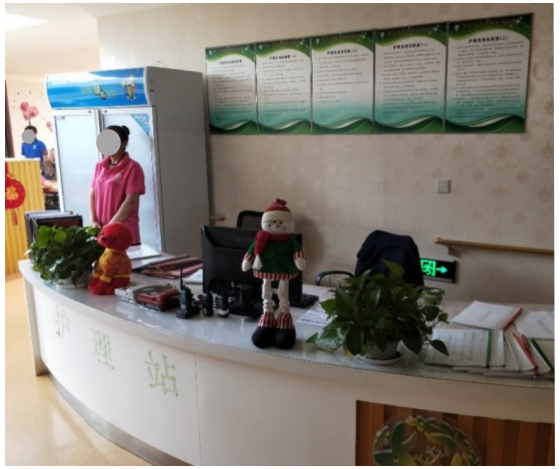

Nursing Station

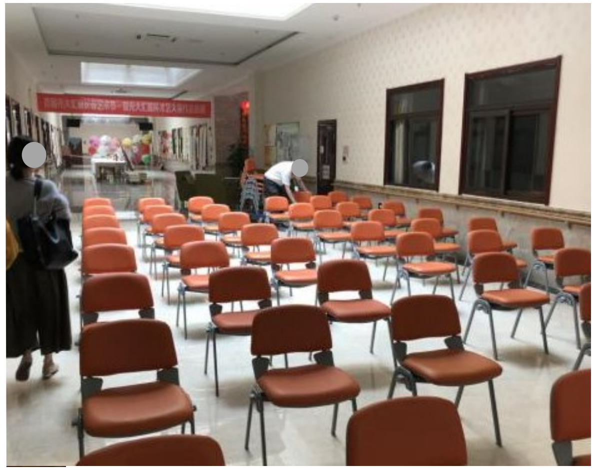

Multi-function Hall

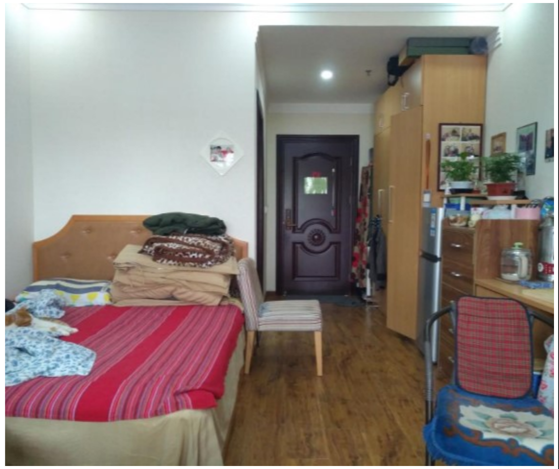

Resident Room

Project 05	Location	Opening Year	Building Area	Number of Beds	Building Area per bed	Building Stories	Occupancy Rate	Types of Residents	Number of Staff	Construction Forms
	Beijing	2018	5865㎡	201	29.18 ㎡/beds	Ground 5	26.9%	Independent; Functional loss	33	Newly Built

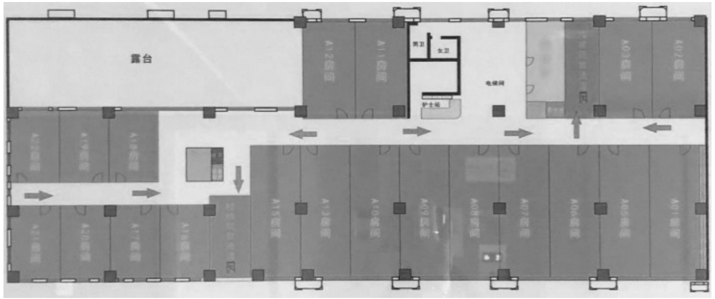
Standard Floor Plan

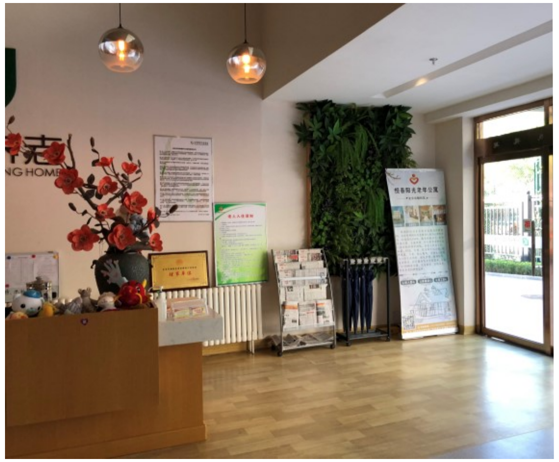
Entry Lobby

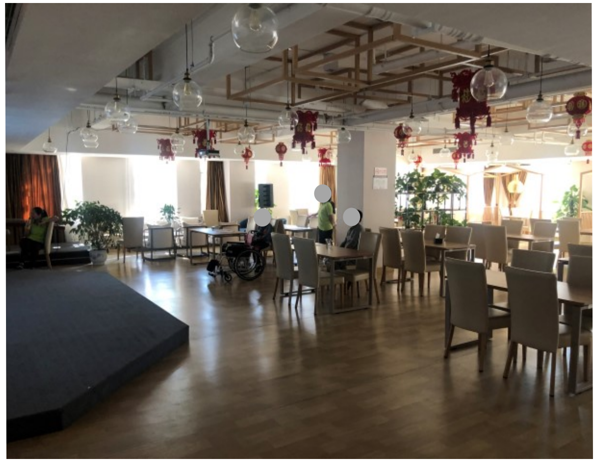
Dining Space

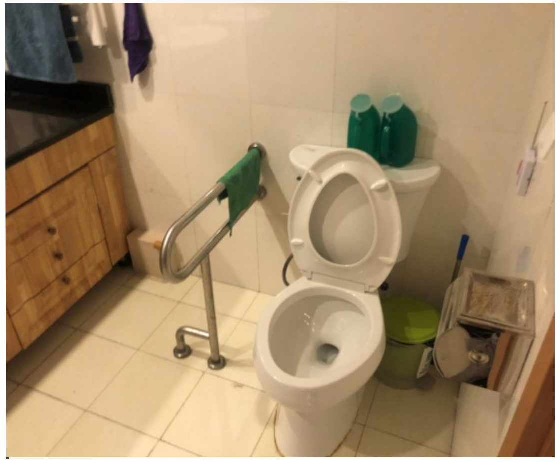
Bathroom within Resident Room

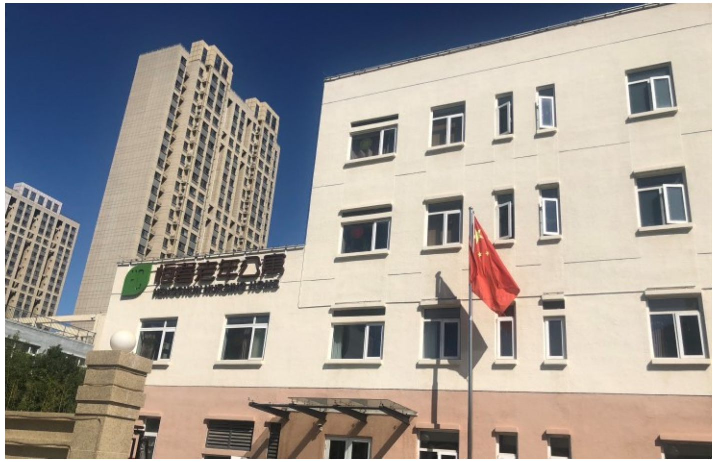
Building Exterior

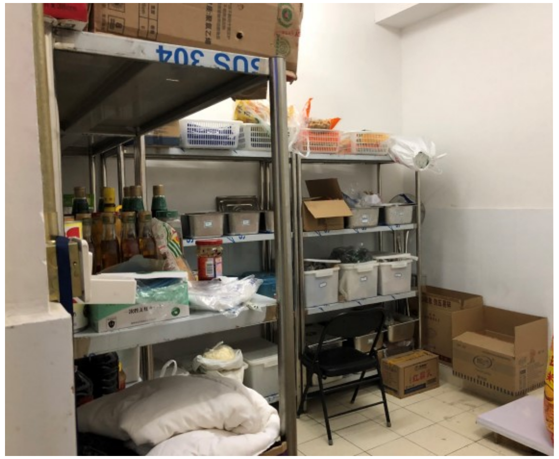
Public Storage Space

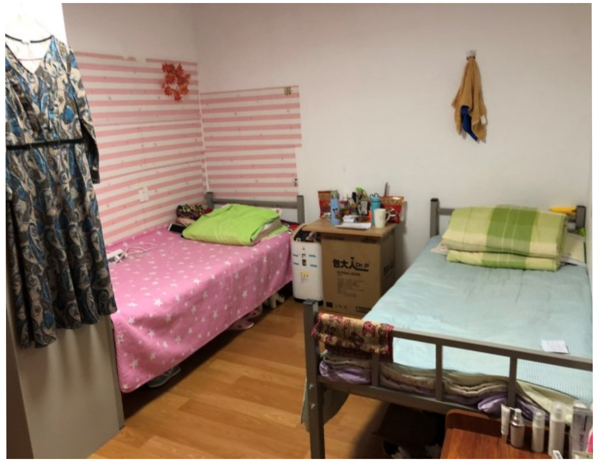
Staff Living Space

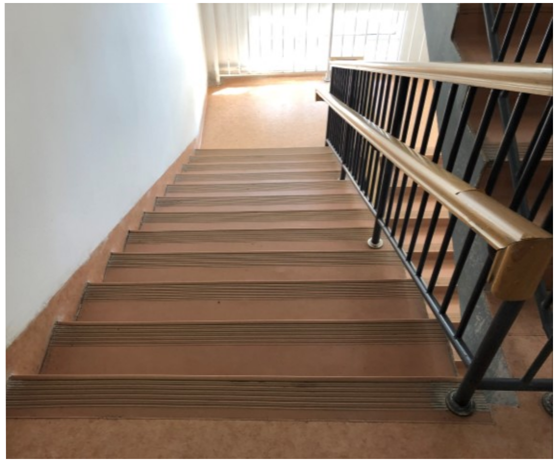
Staircase

Project 06

Location	Opening Year	Building Area	Number of Beds	Building Area per bed	Building Stories	Occupancy Rate	Types of Residents	Number of Staff	Construction Forms
Beijing	2012	1650㎡	68	24.26 ㎡/beds	Ground 2	97.1%	Independent; Functional loss; Dementia	21	Renovation

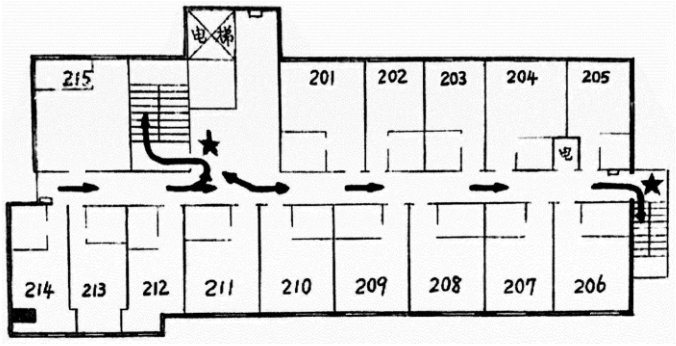

Standard Floor Plan

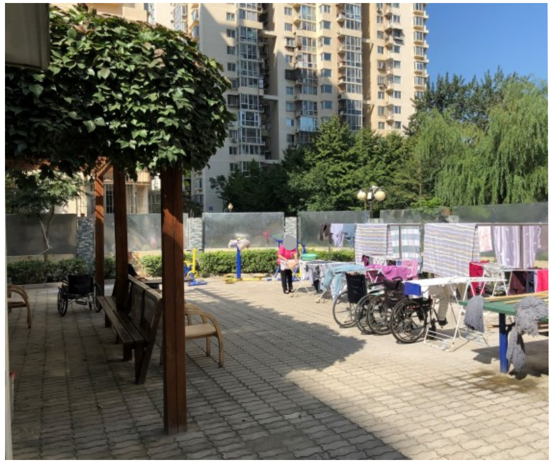
Outdoor Space

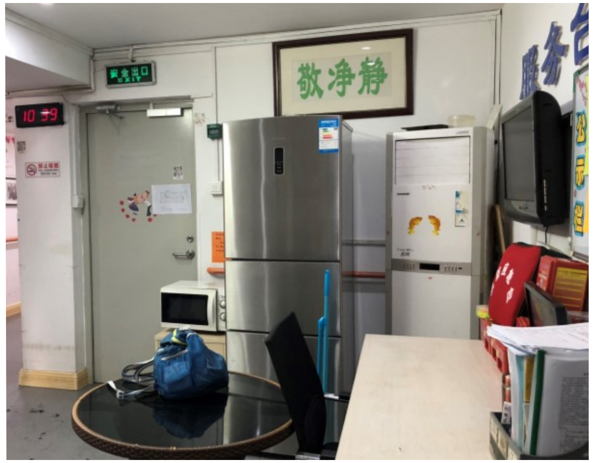

Entry Lobby

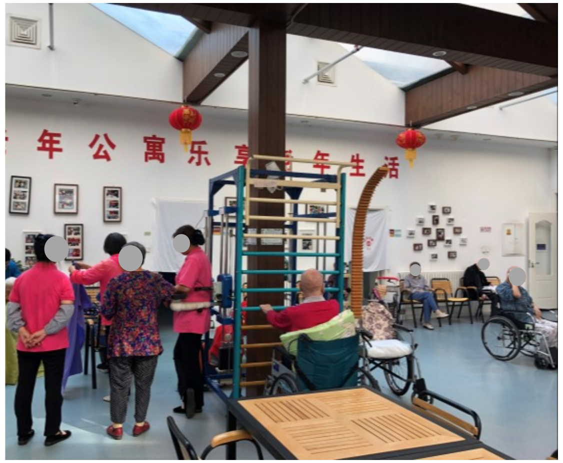
Multi-function Hall

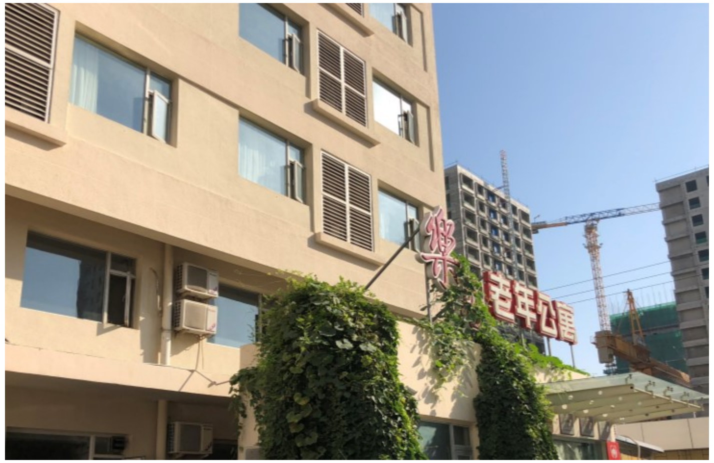
Building Exterior

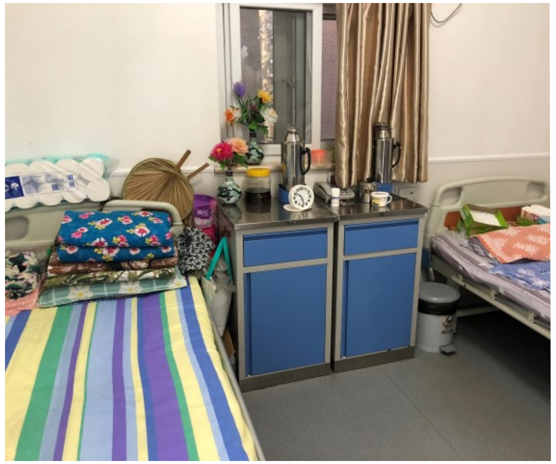
Resident Room

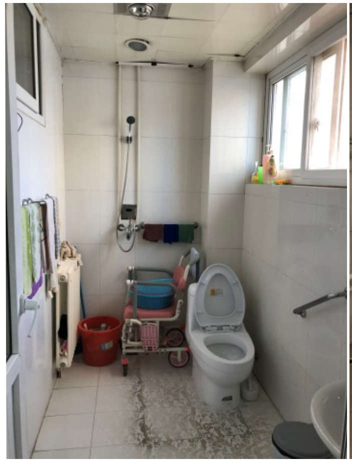
Toilet

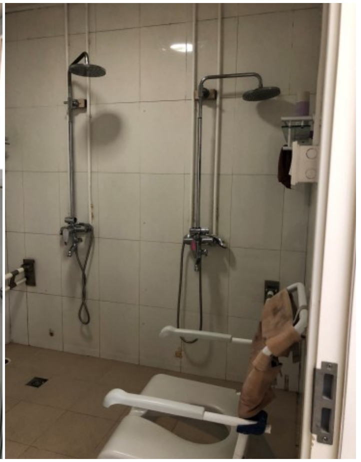
Public Bathroom

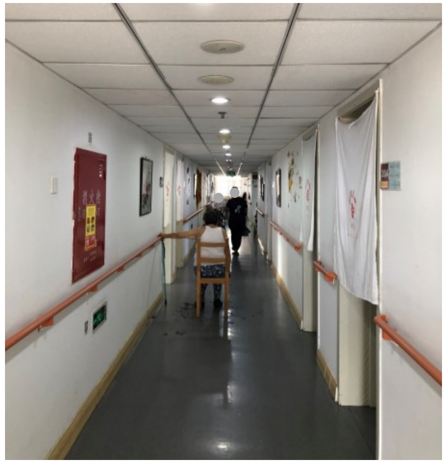
Corridor

Project 07

Location	Opening Year	Building Area	Number of Beds	Building Area per bed	Building Stories	Occupancy Rate	Types of Residents	Number of Staff	Construction Forms
Beijing	2019	4500㎡	75	60.00 ㎡/beds	Ground 2	76.0%	Independent; Functional loss; Dementia	71	Renovation

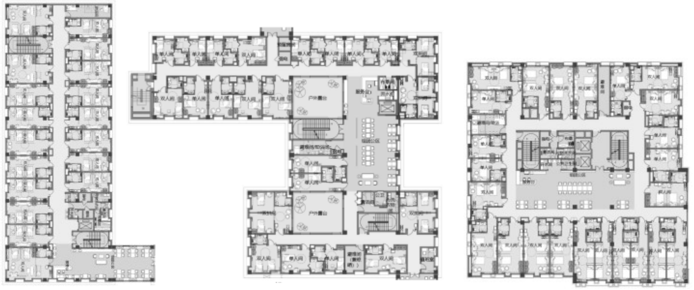

F# E# D#

Standard Floor Plan

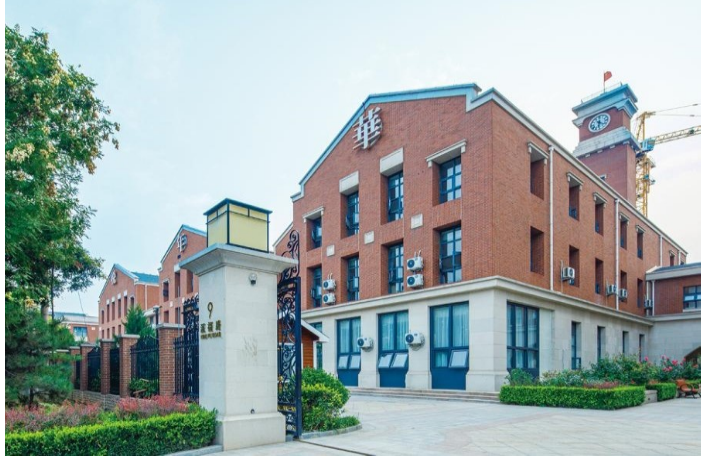

Building Exterior

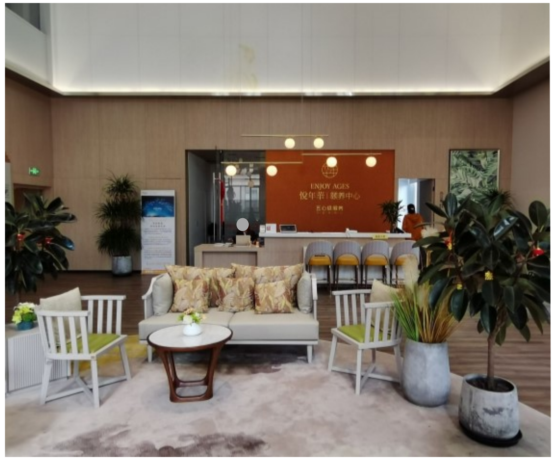

Entry Lobby

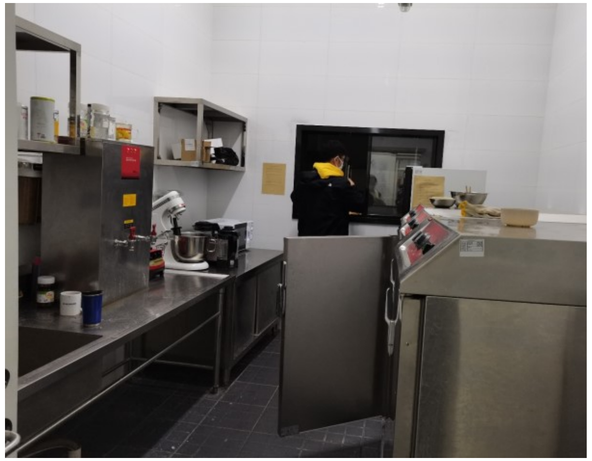

Central Kitchen

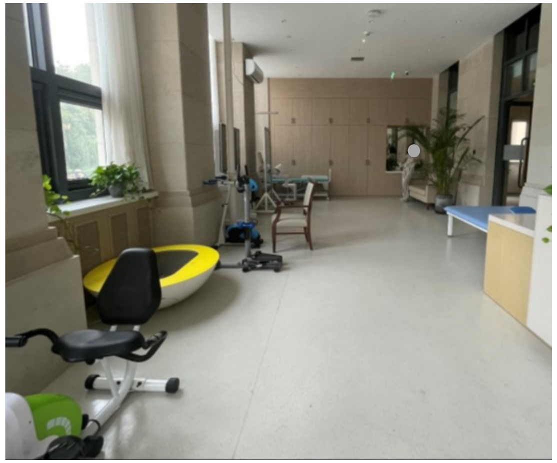

Rehabilitation Space

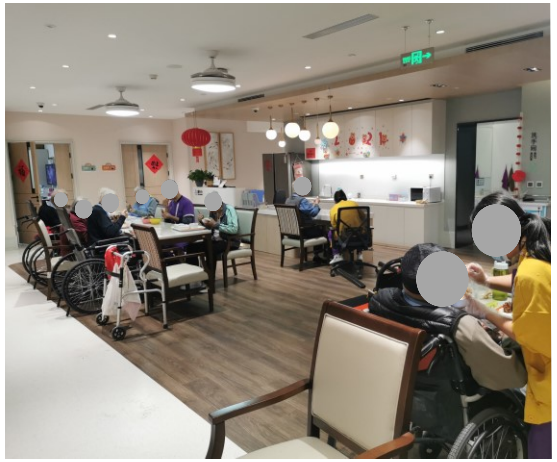

Living Room

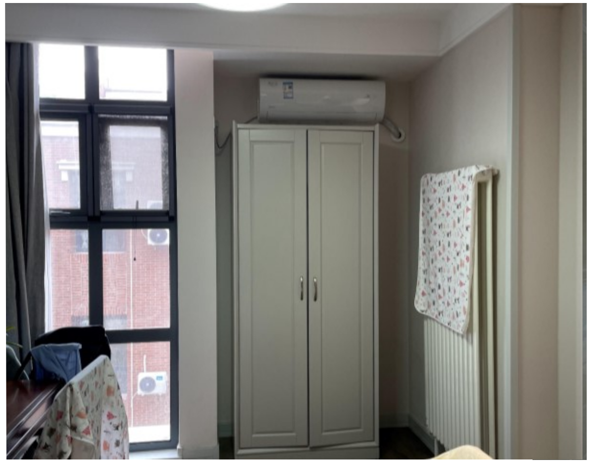

Resident Room

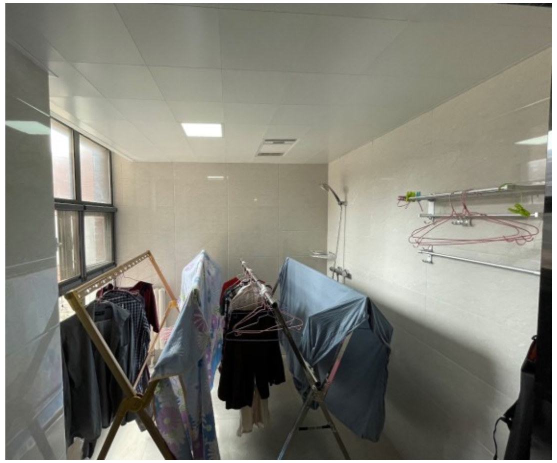

Public Bathroom

Project 08

Location	Opening Year	Building Area	Number of Beds	Building Area per bed	Building Stories	Occupancy Rate	Types of Residents	Number of Staff	Construction Forms
Beijing	2012	7100㎡	157	45.22 ㎡/beds	Ground 4 Underground 1	81.5%	Independent; Functional loss	82	Renovation

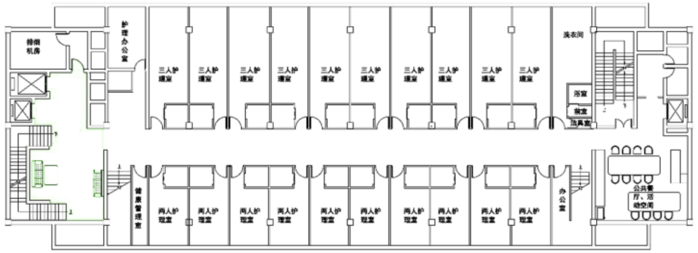

Standard Floor Plan

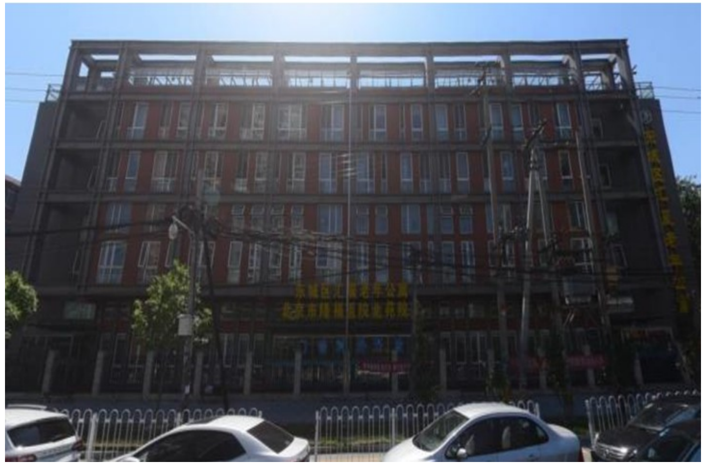

Building Exterior

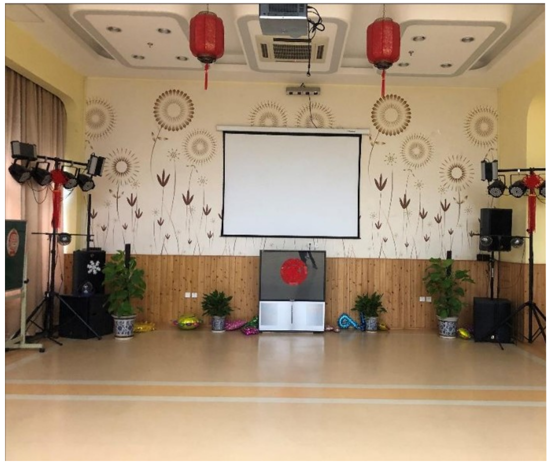

Entry Lobby

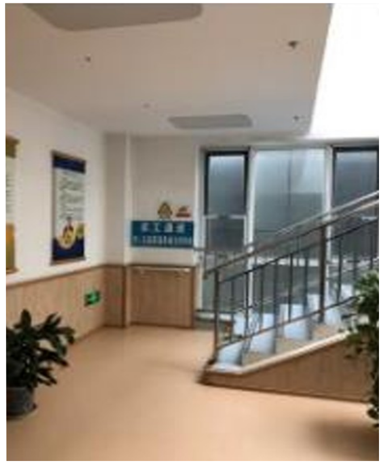

Staircase

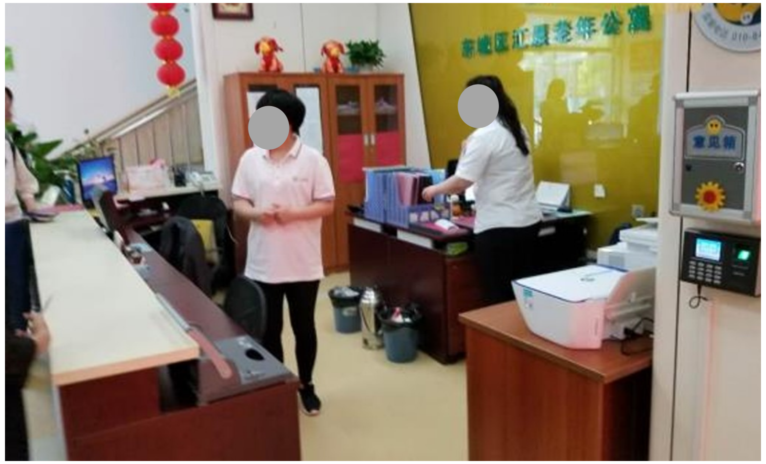

Reception & Staff Working Space

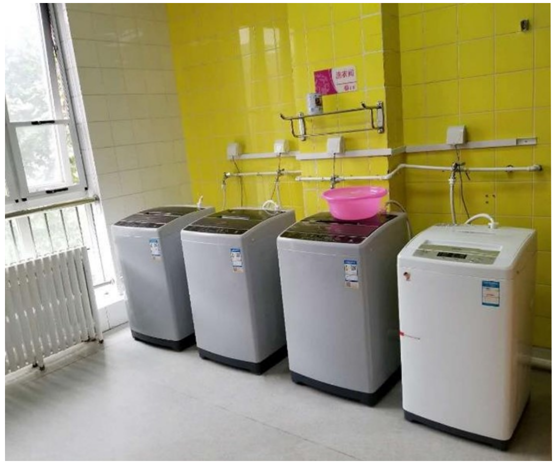

Laundry Space

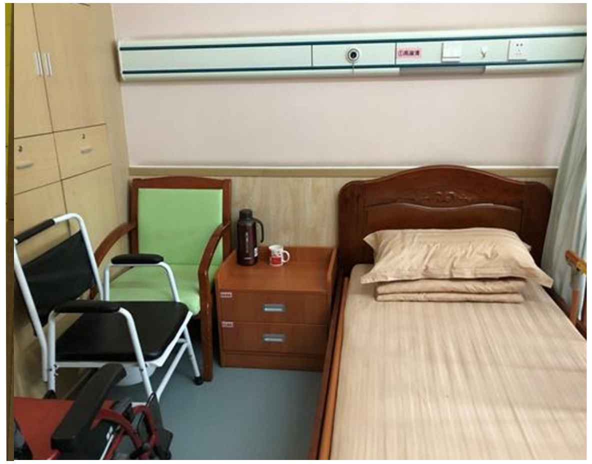

Resident Room

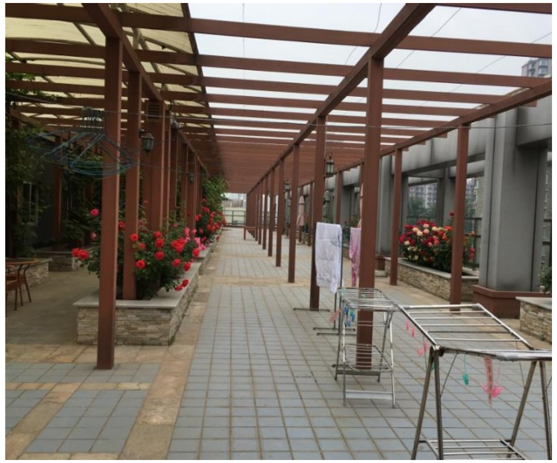

Rooftop Garden

Project 09	Location	Opening Year	Building Area	Number of Beds	Building Area per bed	Building Stories	Occupancy Rate	Types of Residents	Number of Staff	Construction Forms
	Beijing	2017	9246㎡	190	48.66 ㎡/beds	Ground 5 Underground 2	24.7%	Independent; Functional loss; Dementia	80	Newly Built

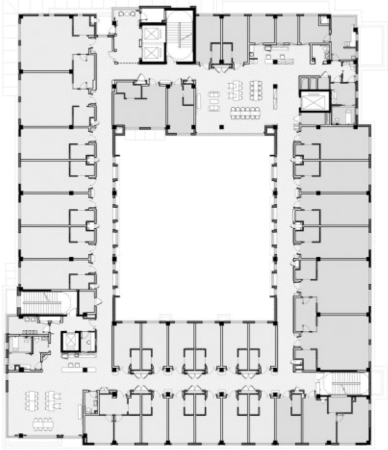

Standard Floor Plan

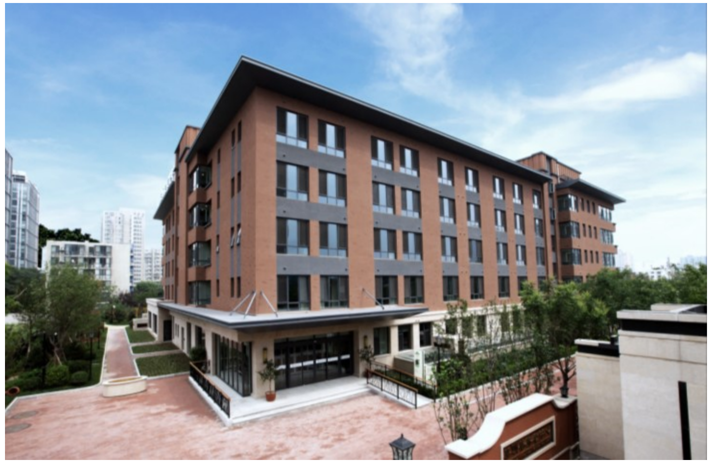

Building Exterior

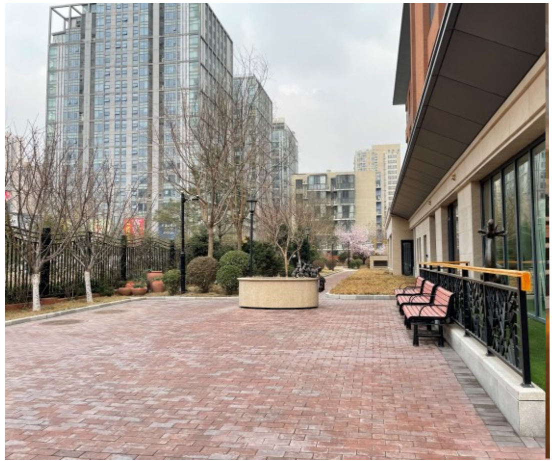

Outdoor Space

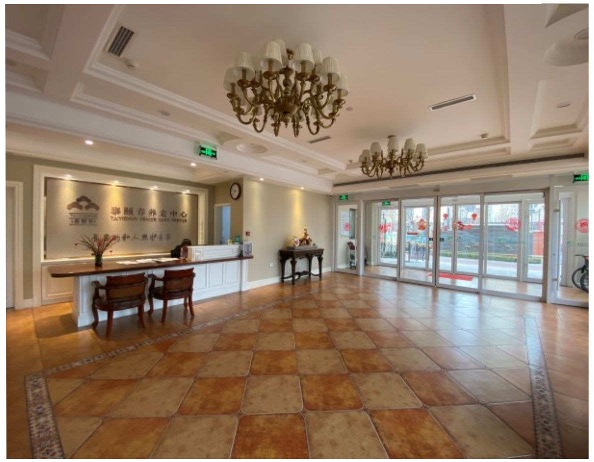

Entry Lobby

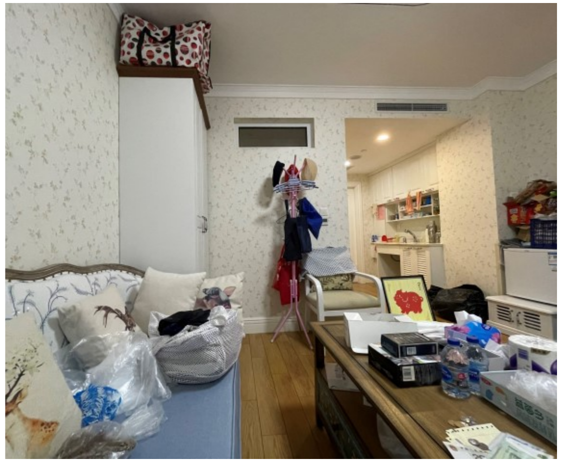

Resident Room

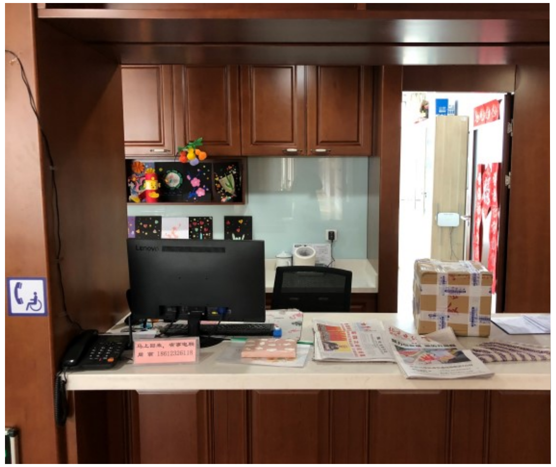

Nursing Station

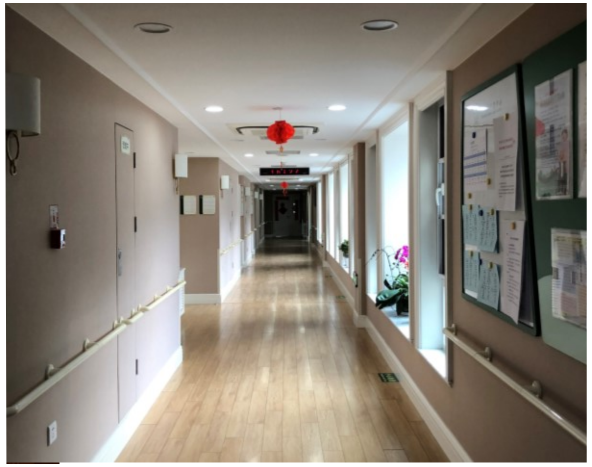

Corridor

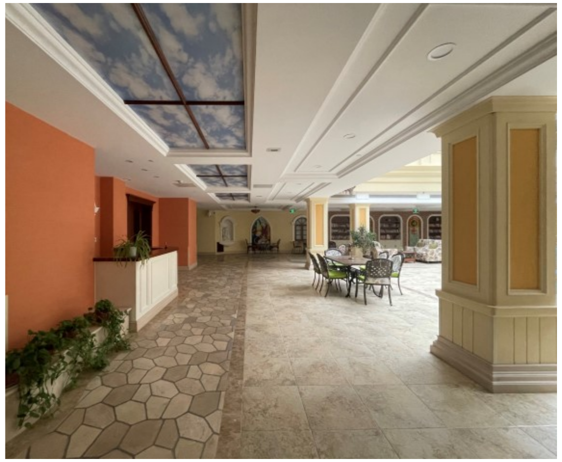

Activity Space

Project	Location	Opening Year	Building Area	Number of Beds	Building Area per bed	Building Stories	Occupancy Rate	Types of Residents	Number of Staff	Construction Forms
**Project 10**	Beijing	2016	1278㎡	28	45.64 ㎡/beds	Ground 3 Underground 1	Unknown	Independent; Functional loss; Dementia	Unknown	Renovation

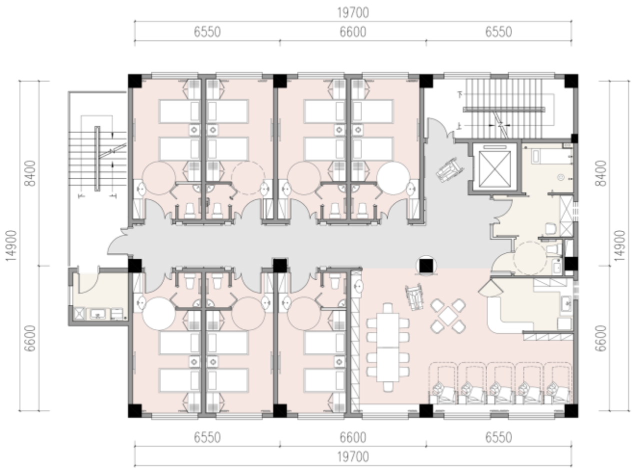

Standard Floor Plan

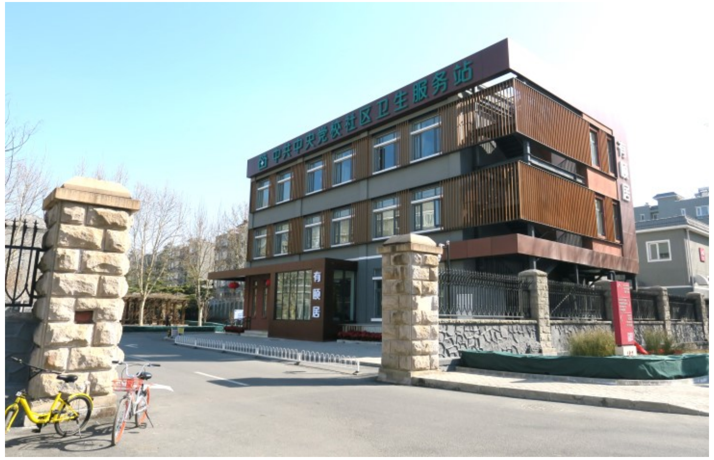

Building Exterior

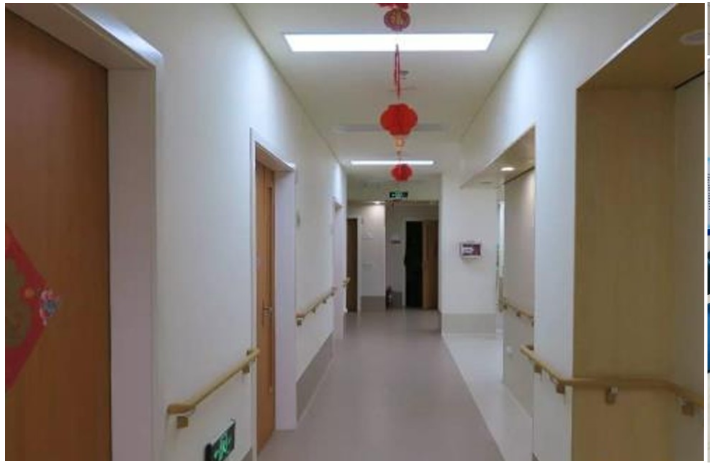

Corridor

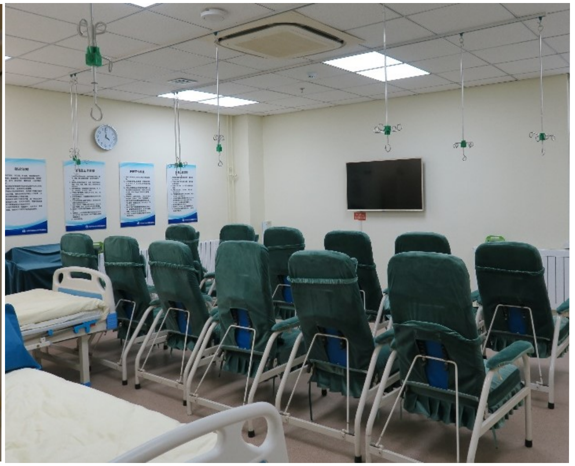

Medical Space

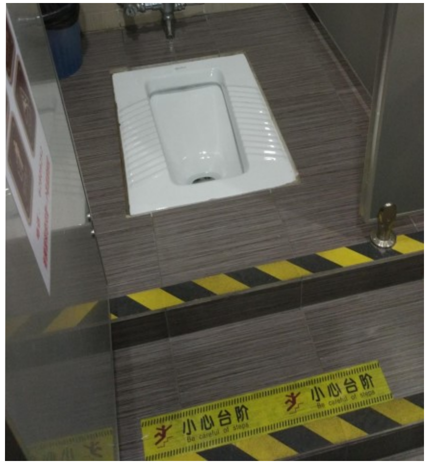

Toilet

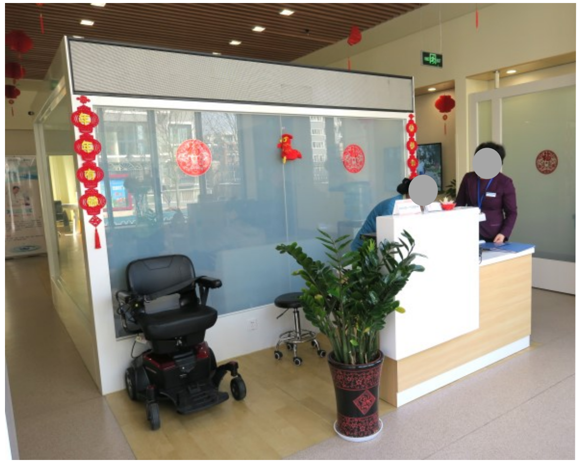

Nursing Station

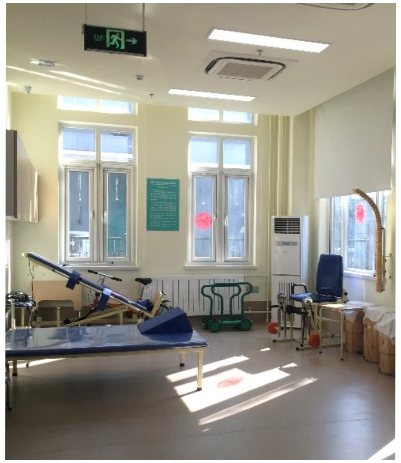

Rehabilitation Space

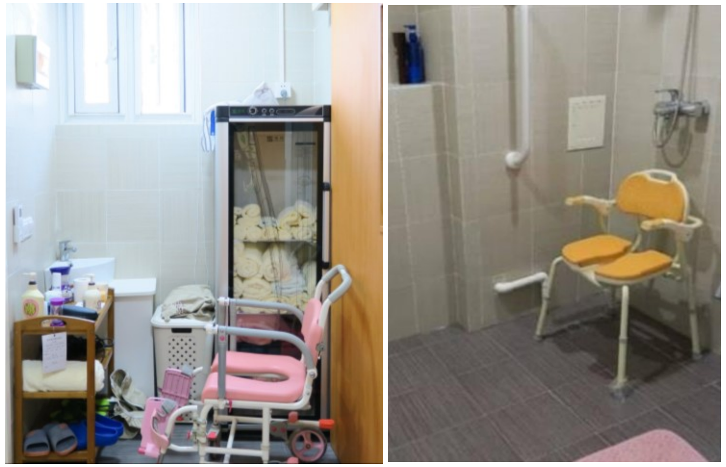

Public Bathroom

Project 11	Location	Opening Year	Building Area	Number of Beds	Building Area per bed	Building Stories	Occupancy Rate	Types of Residents	Number of Staff	Construction Forms
	Chongqing	2018	8215㎡	155	53.00 ㎡/beds	Ground 9	80.0%	Independent; Functional loss; Dementia	44	Newly Built

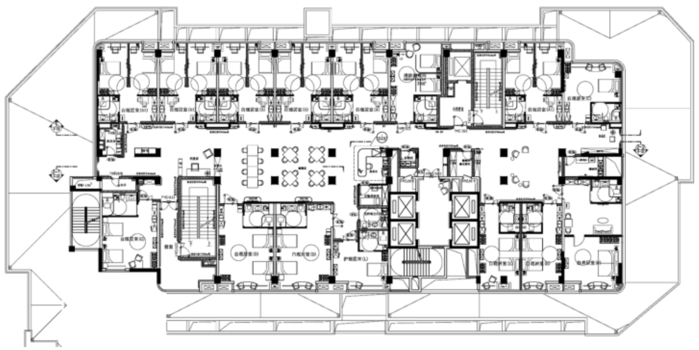
Standard Floor Plan

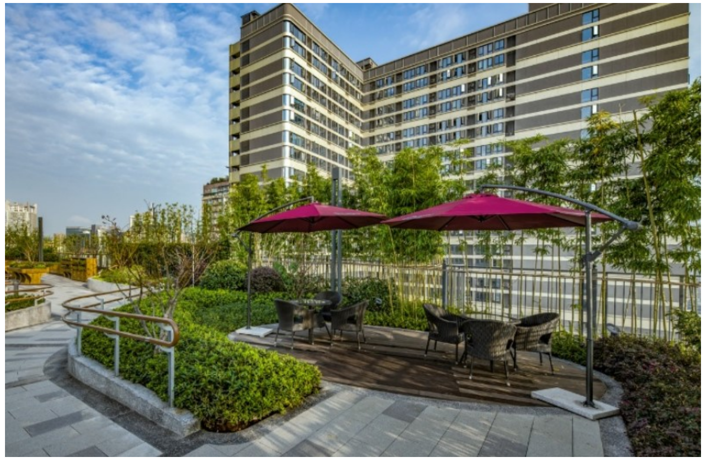
Building Exterior

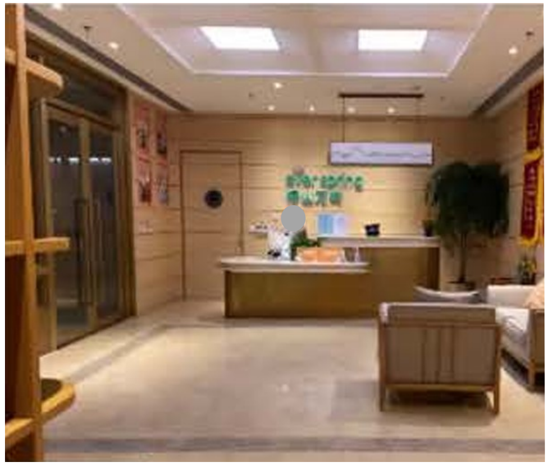
Entry Lobby

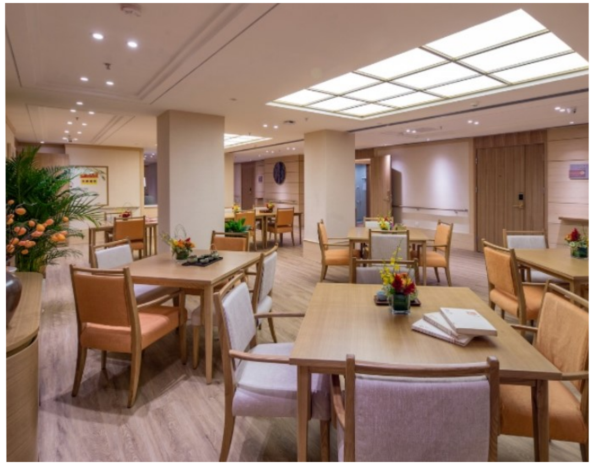
Living Room

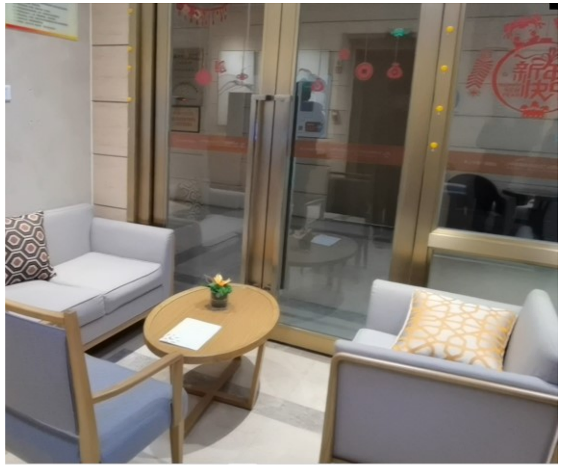
Entry Lobby

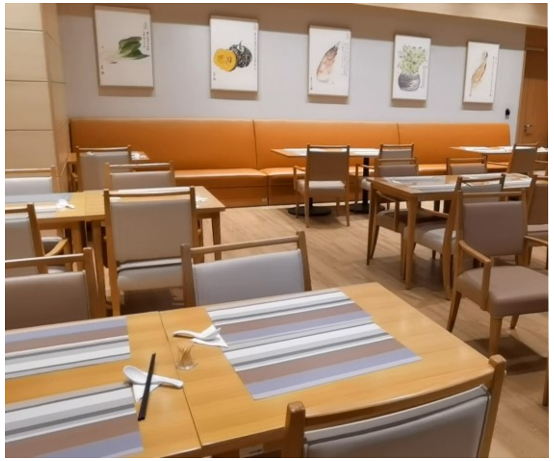
Dining Space

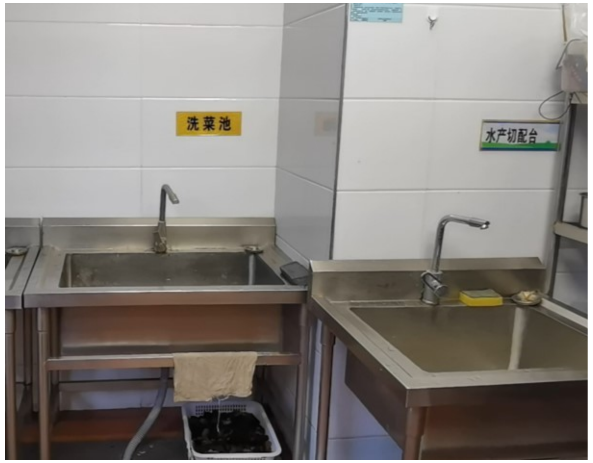

Central Kitchen

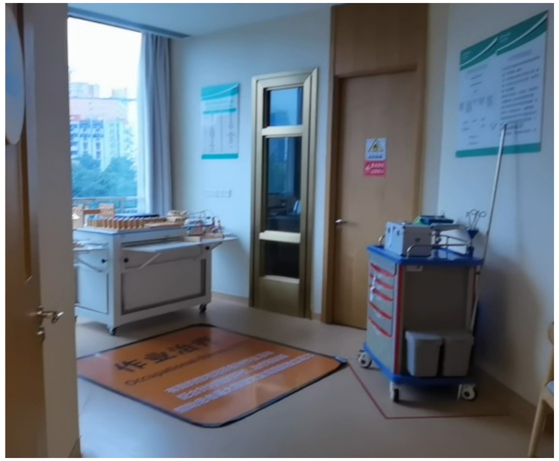
Rehabilitation Space

Project 12

Location	Opening Year	Building Area	Number of Beds	Building Area per bed	Building Stories	Occupancy Rate	Types of Residents	Number of Staff	Construction Forms
Foshan	2000	2366㎡	98	24.14 ㎡/beds	Ground 5	49.0%	Independent; Functional loss; Dementia	23	Renovation

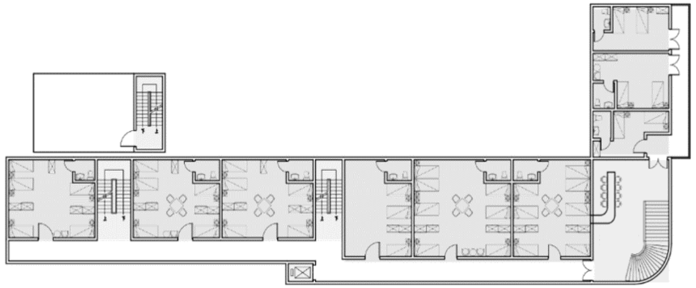

Standard Floor Plan

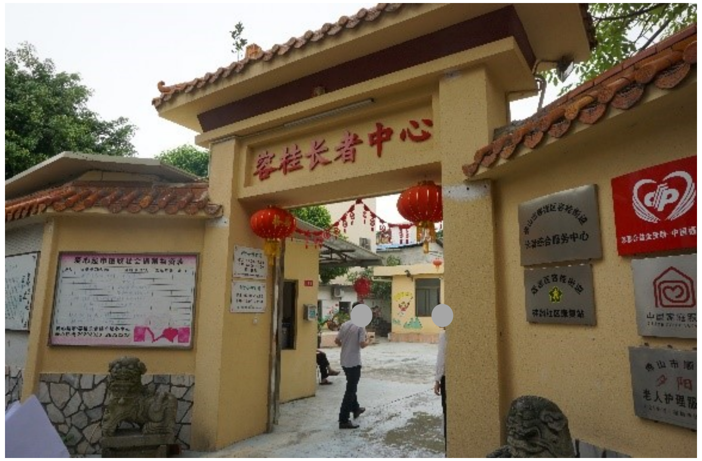

Building Exterior

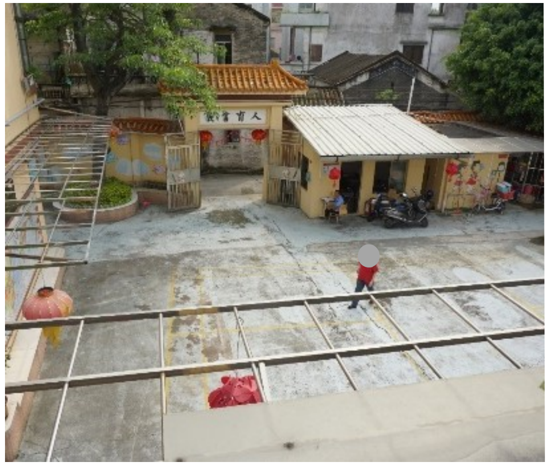

Outdoor Space

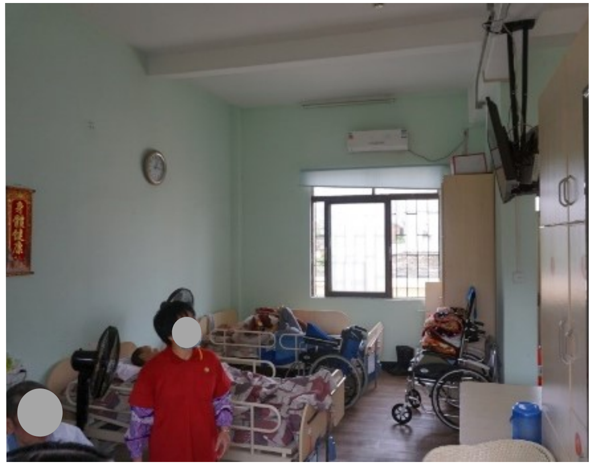

Resident Room

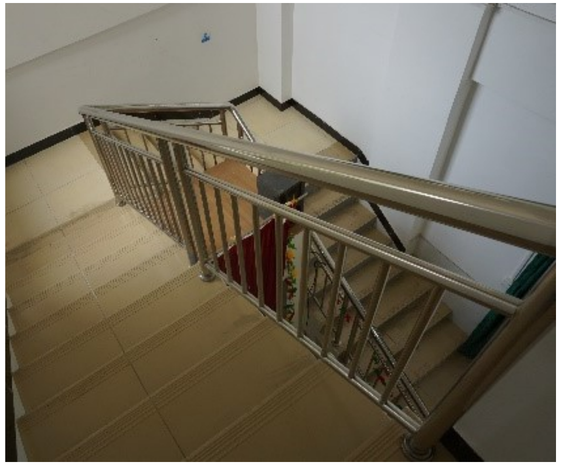

Staircase

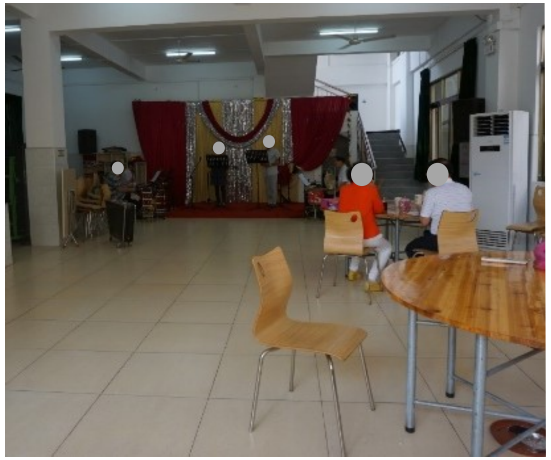

Dining Space

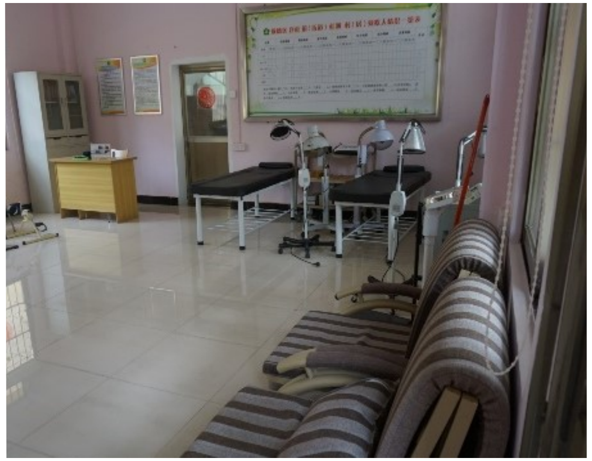

Rehabilitation Space

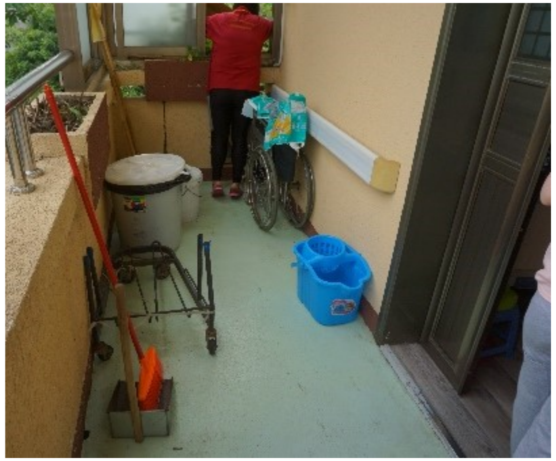

Cleaning Space

Project 13	Location	Opening Year	Building Area	Number of Beds	Building Area per bed	Building Stories	Occupancy Rate	Types of Residents	Number of Staff	Construction Forms
	Foshan	1998	5034㎡	174	28.93 ㎡/beds	Ground 4	95.4%	Independent; Functional loss; Dementia	46	Newly Built

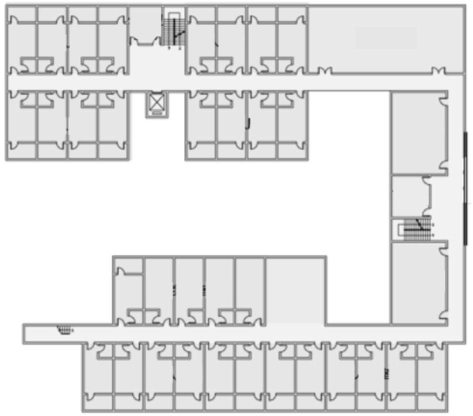

Standard Floor Plan

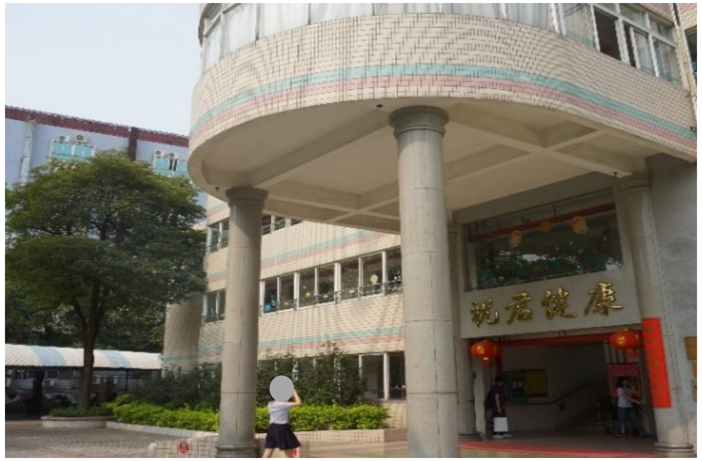

Building Exterior

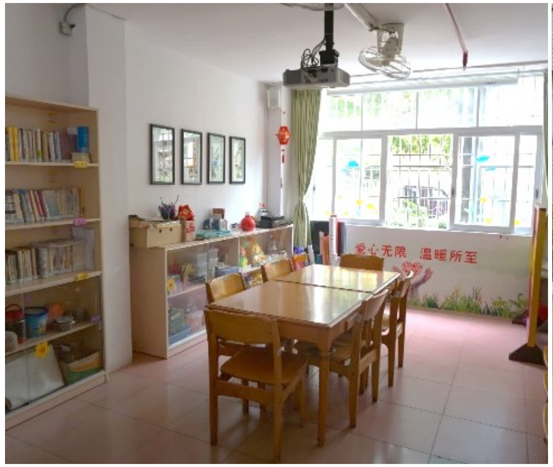

Activity Space

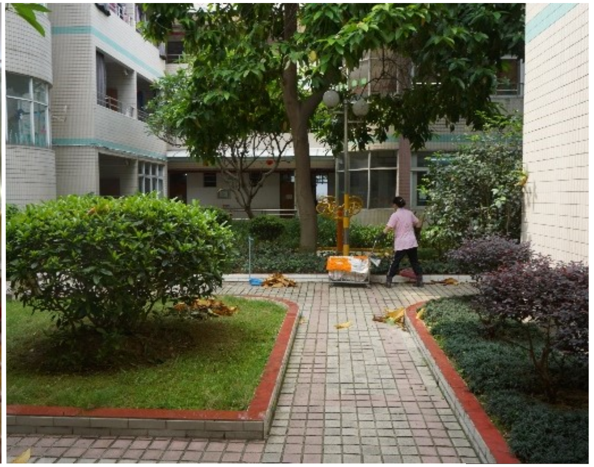

Outdoor Space

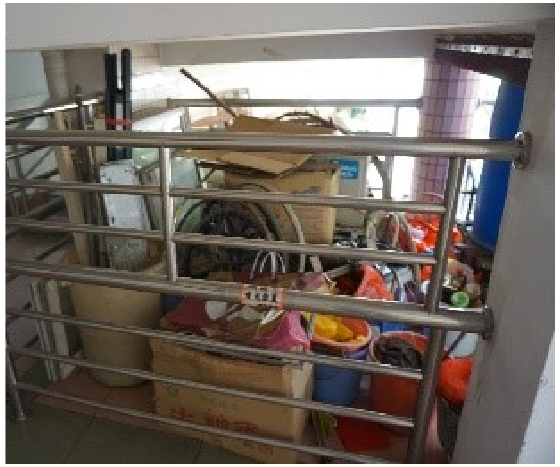

Storage Space

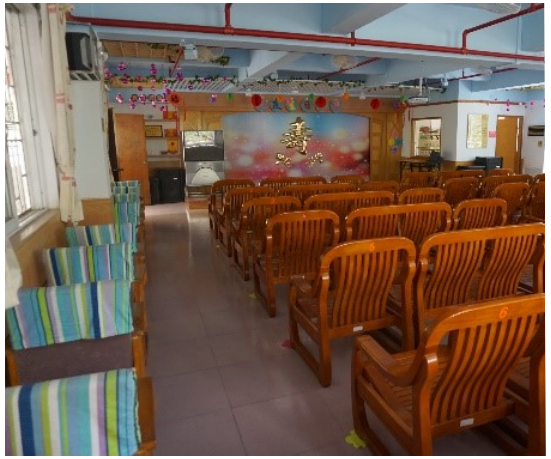

Multi-function Room

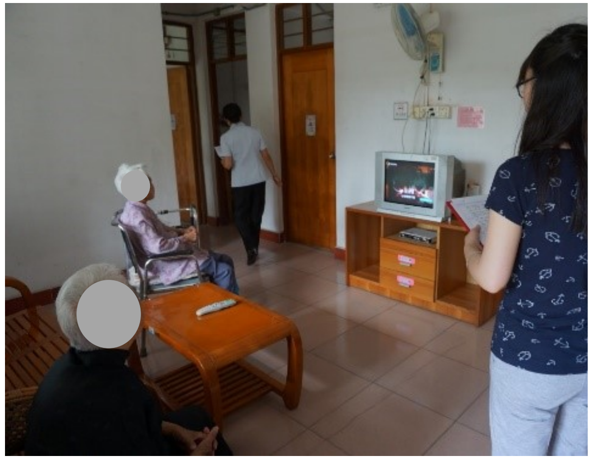

Resident Room

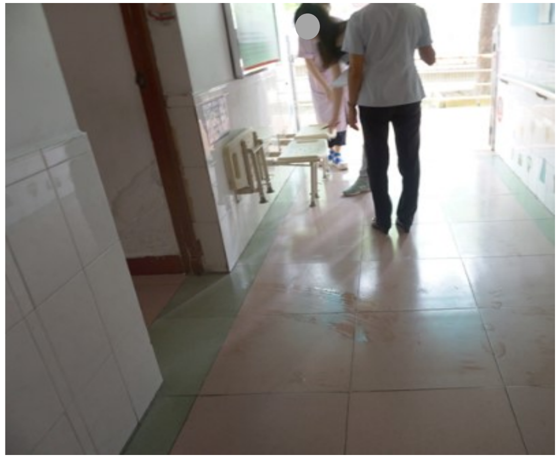

Corridor

Project 14

Location	Opening Year	Building Area	Number of Beds	Building Area per bed	Building Stories	Occupancy Rate	Types of Residents	Number of Staff	Construction Forms
Foshan	2014	5400㎡	130	41.54 ㎡/beds	Ground 4	84.6%	Independent; Functional loss; Dementia	41	Renovation

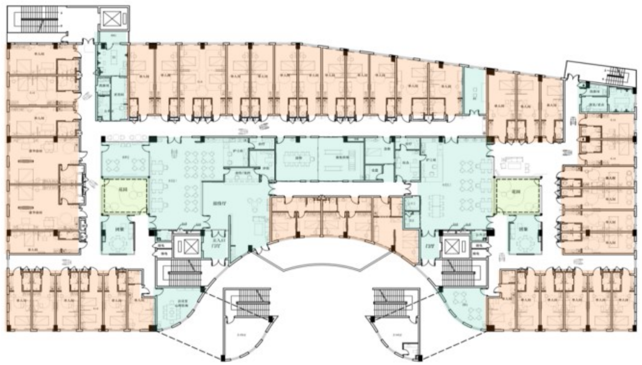

Standard Floor Plan

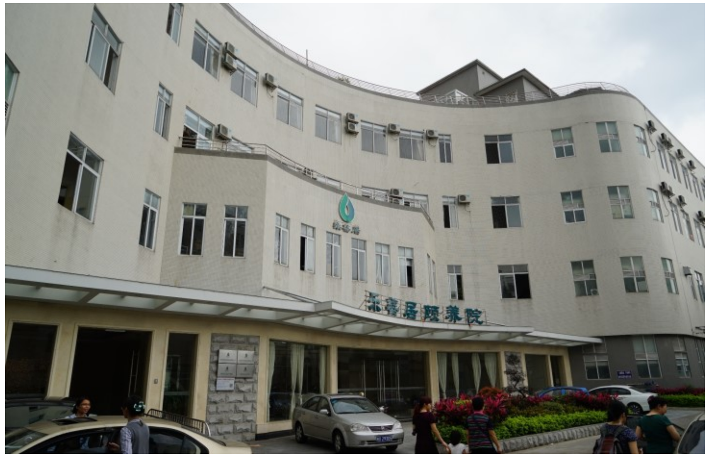

Building Exterior

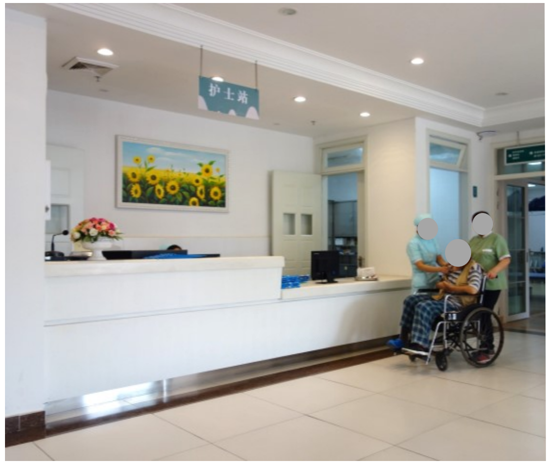

Nursing Station

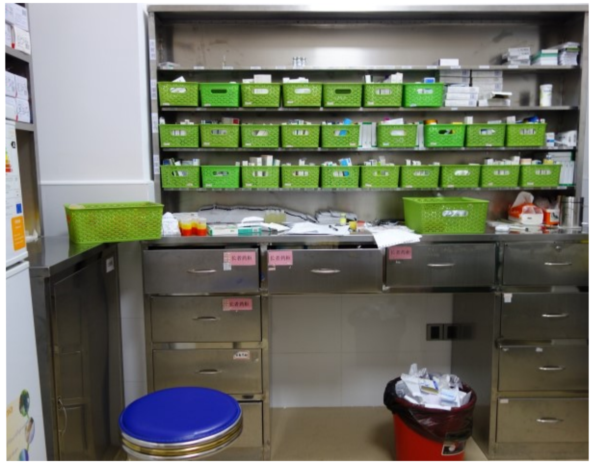

Medical Space

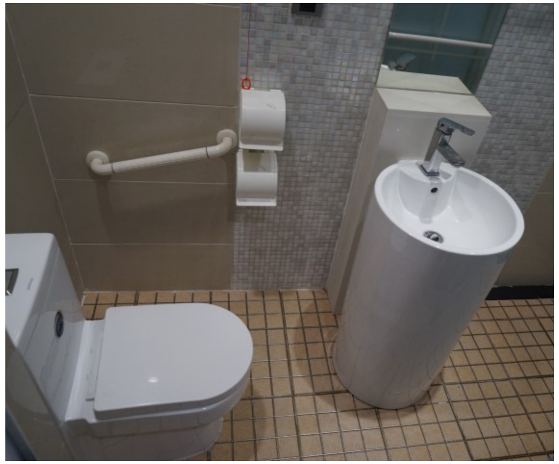

Toilet

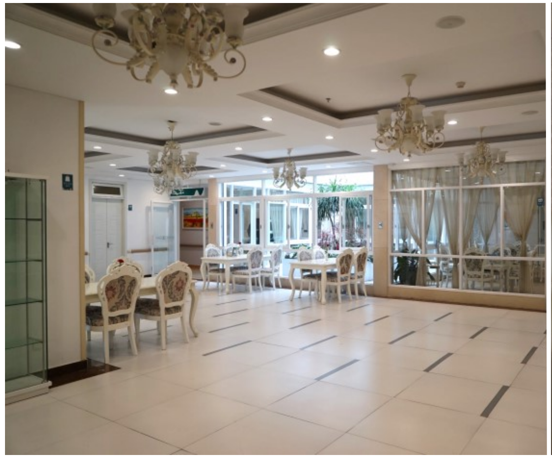

Living Room

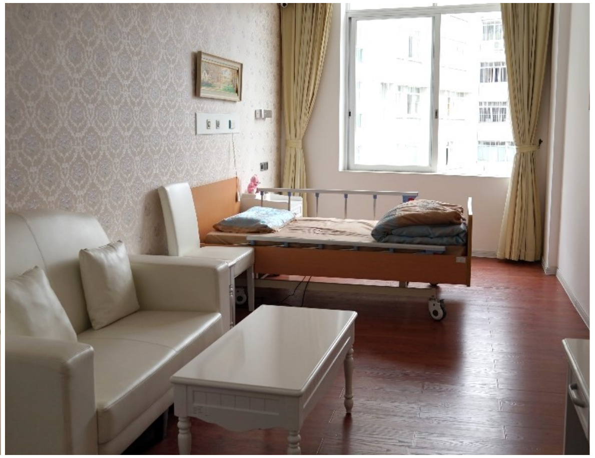

Resident Room

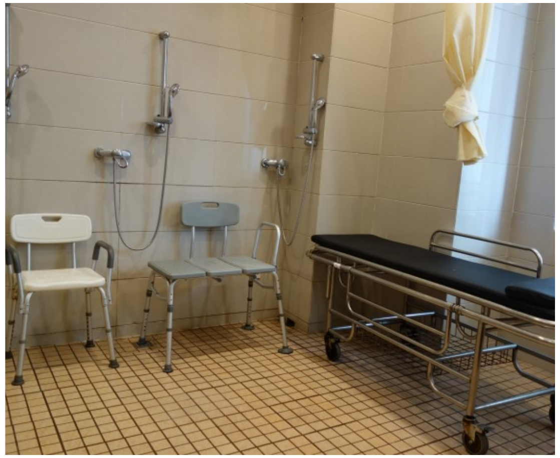

Public Bathroom

Project 15	Location	Opening Year	Building Area	Number of Beds	Building Area per bed	Building Stories	Occupancy Rate	Types of Residents	Number of Staff	Construction Forms
	Foshan	2017	2000㎡	94	21.28 ㎡/beds	Ground 4	100.0%	Independent; Functional loss	Unknown	Renovation

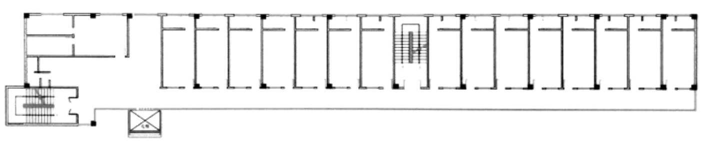

Standard Floor Plan

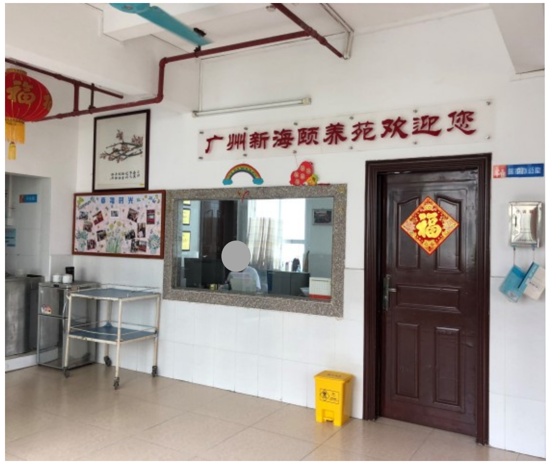

Staff Working Space

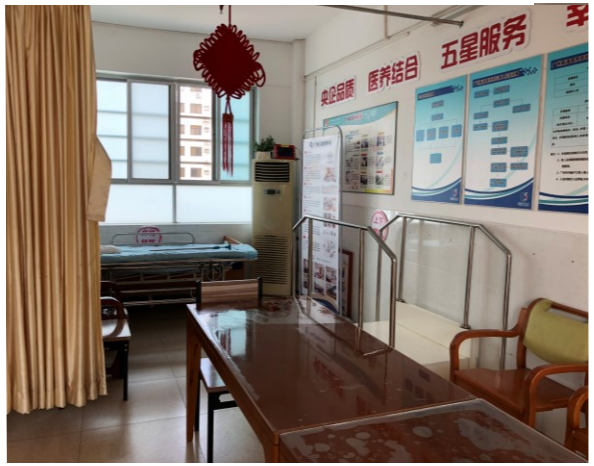

Living Room

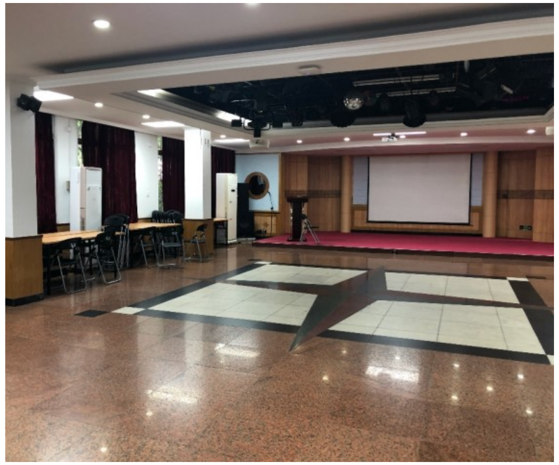

Multi-function Hall

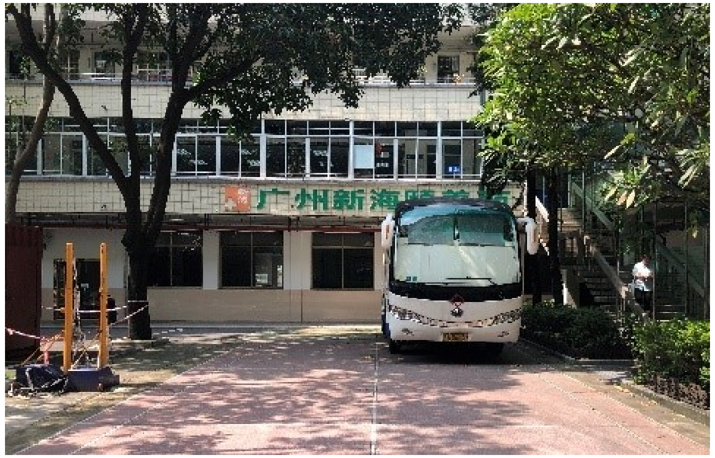

Building Exterior

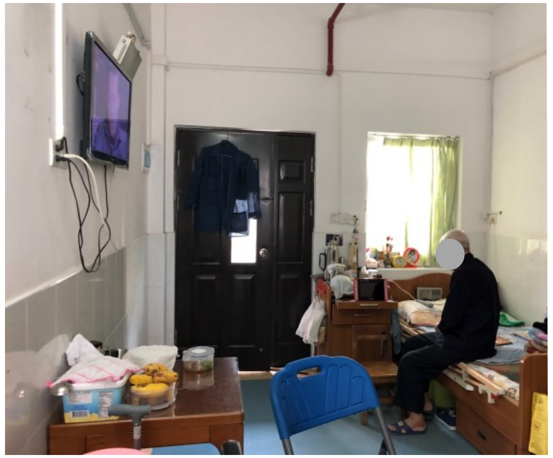

Resident Room

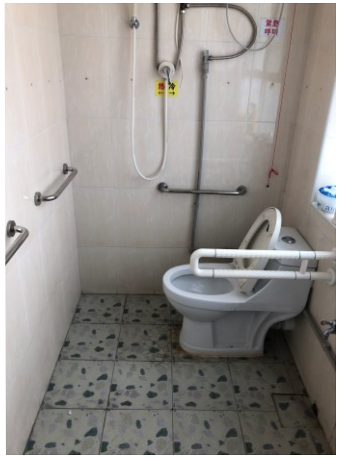

Toilet

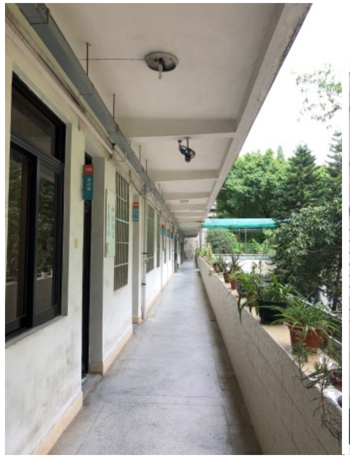

Corridor

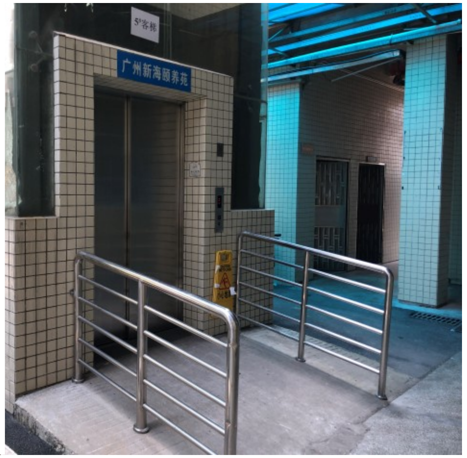

Elevator

Project 16	Location	Opening Year	Building Area	Number of Beds	Building Area per bed	Building Stories	Occupancy Rate	Types of Residents	Number of Staff	Construction Forms
	Shanghai	2012	14000㎡	445	31.46 ㎡/beds	Ground 17	96.6%	Independent; Functional loss; Dementia	140	Renovation

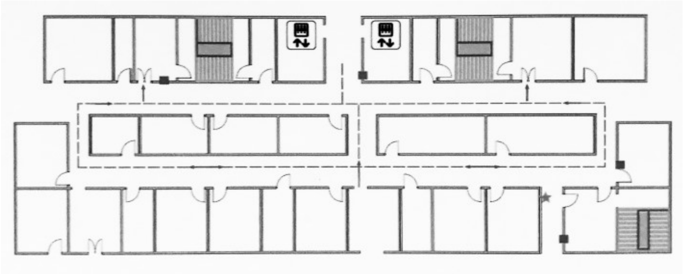

Standard Floor Plan

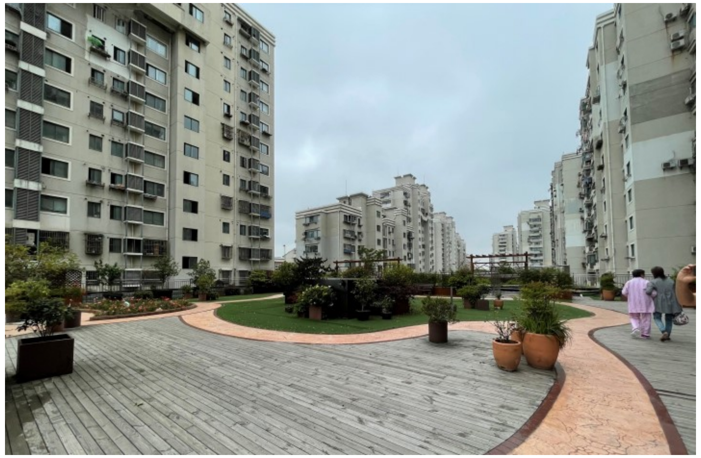

Rooftop Garden

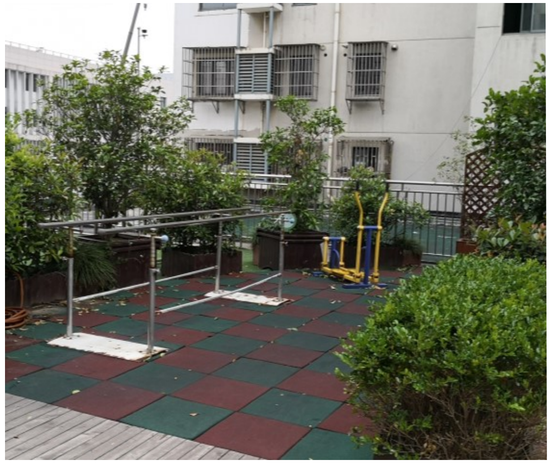

Outdoor Space

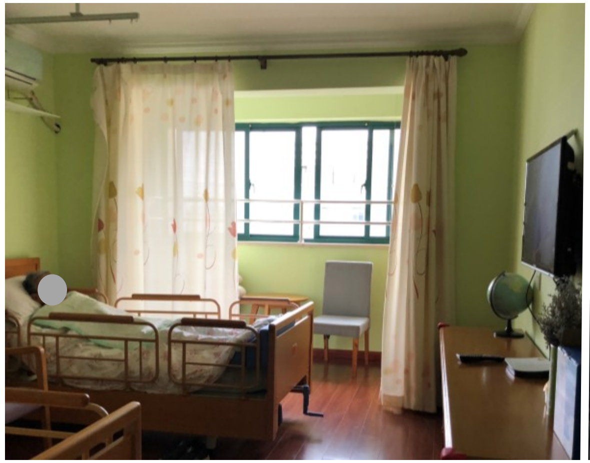

Resident Room

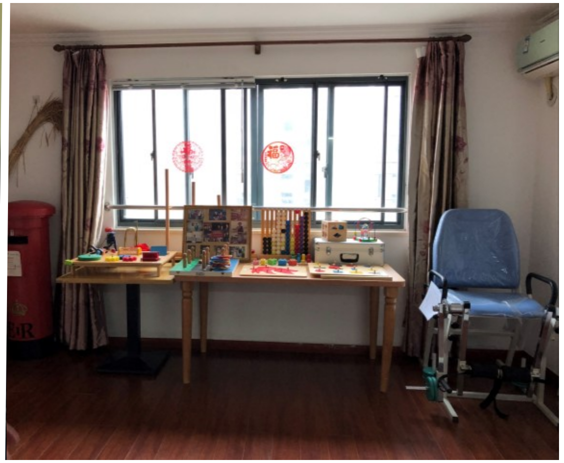

Living Room

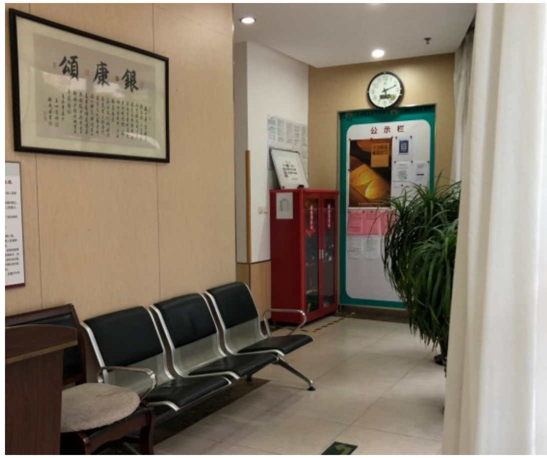

Entry Lobby

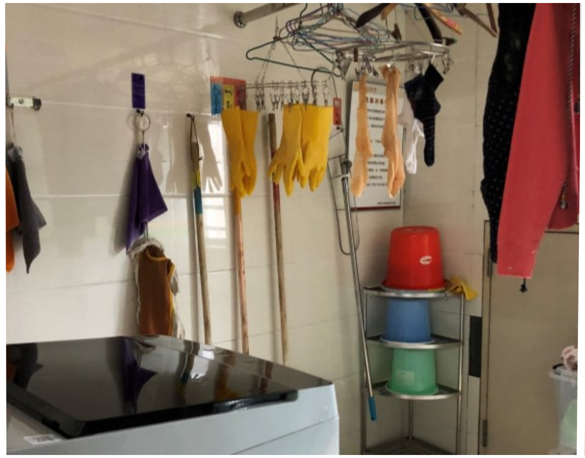

Laundry Space

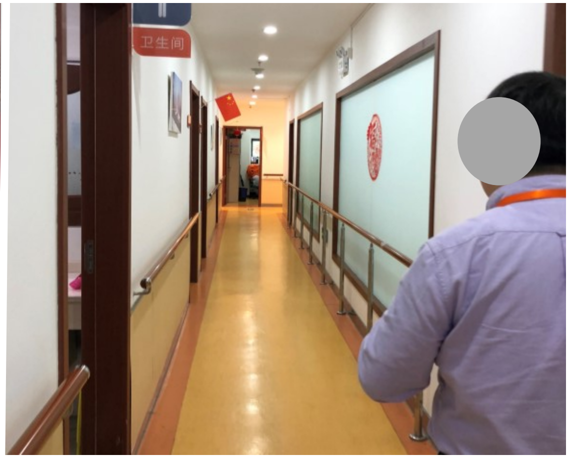

Corridor

Project 17	Location	Opening Year	Building Area	Number of Beds	Building Area per bed	Building Stories	Occupancy Rate	Types of Residents	Number of Staff	Construction Forms
	Chengdu	2017	3000㎡	71	42.25 ㎡/beds	Ground 6	50.7%	Independent; Functional loss	35	Renovation

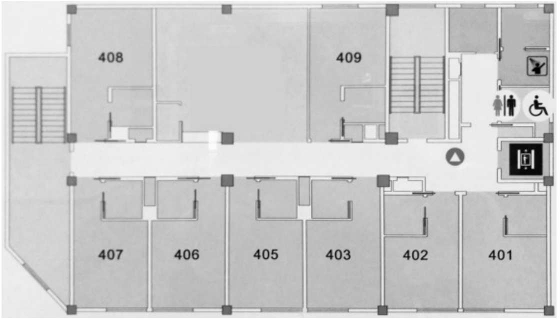

Standard Floor Plan

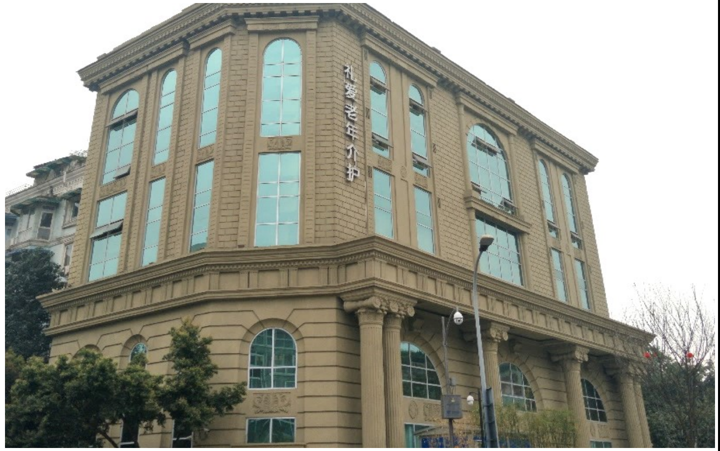

Building Exterior

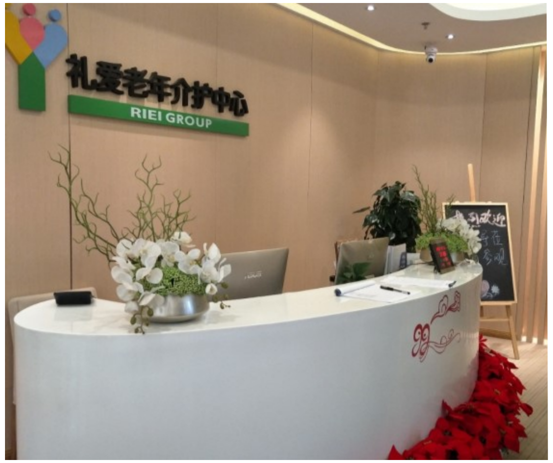

Entry Lobby

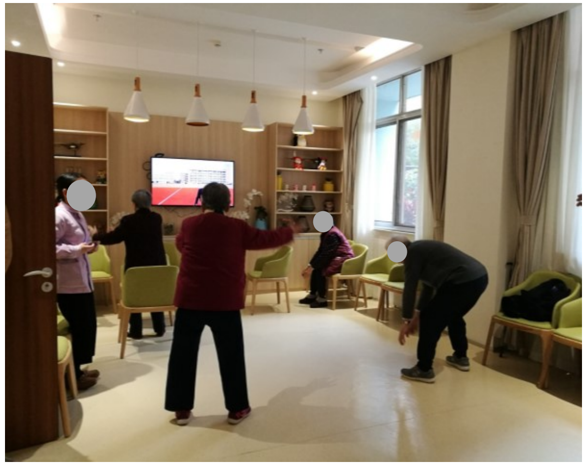

Activity Space

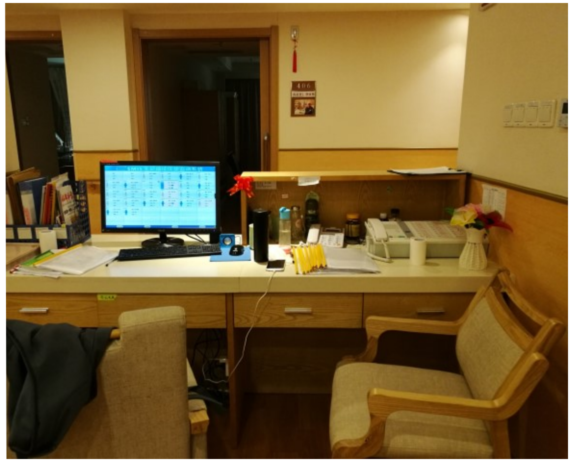

Nursing Station

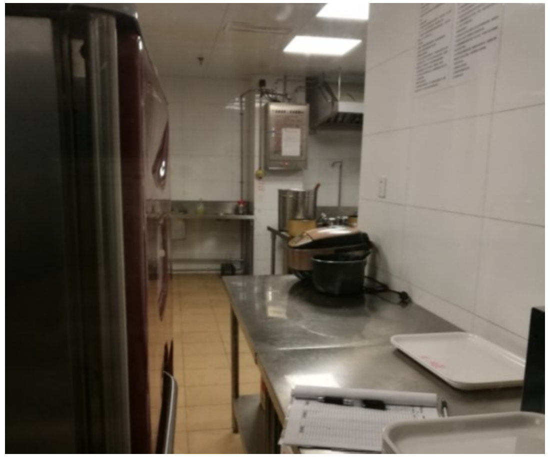

Central Kitchen

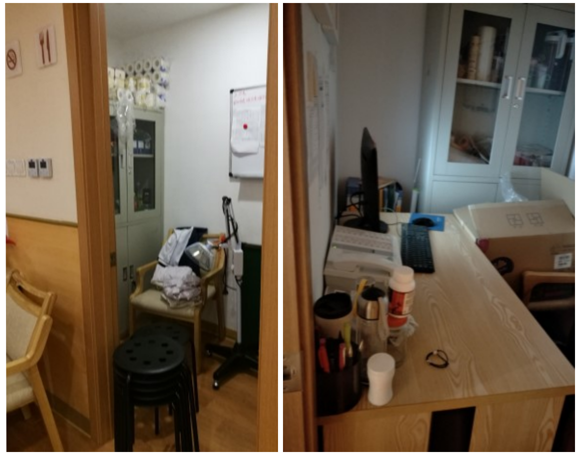

Staff Working Space

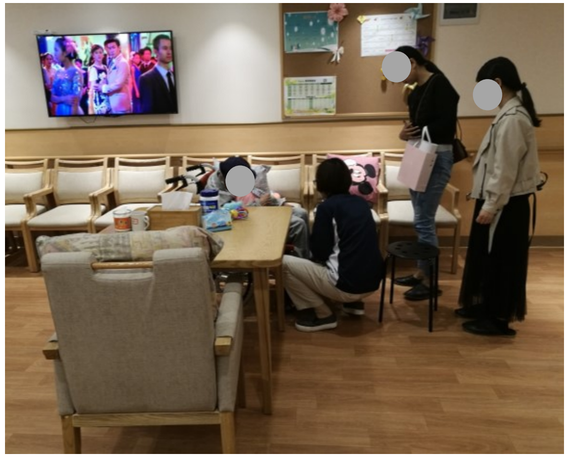

Living Room

Project	Location	Opening Year	Building Area	Number of Beds	Building Area per bed	Building Stories	Occupancy Rate	Types of Residents	Number of Staff	Construction Forms
Project 18	Hangzhou	2020	13600㎡	123	110.57 ㎡/beds	Ground 5 Underground 1	11.4%	Independent; Functional loss; Dementia	50	Newly Built

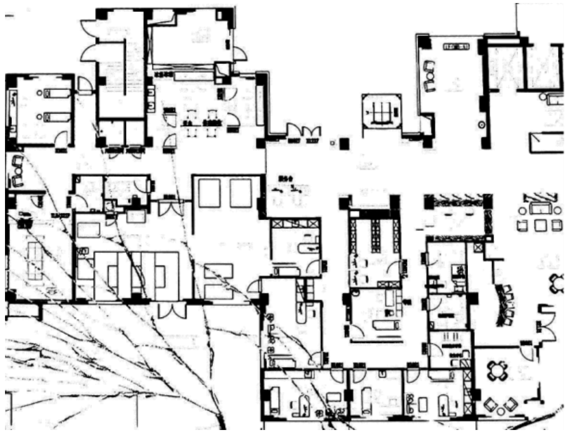
Standard Floor Plan

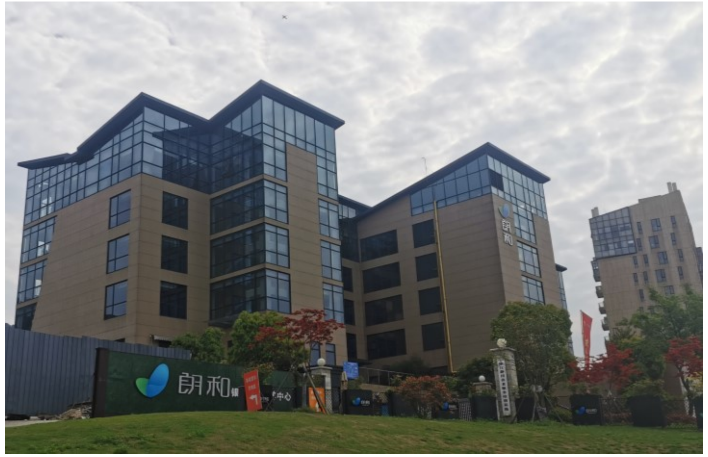

Building Exterior

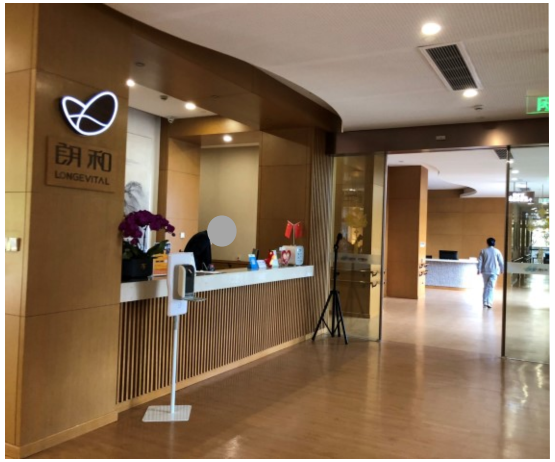

Entry Lobby

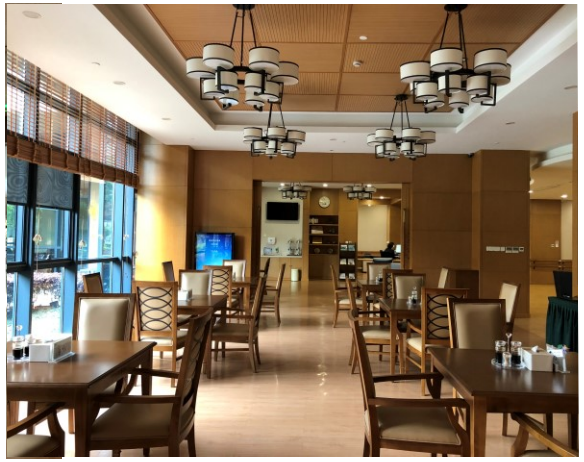
Dining Space

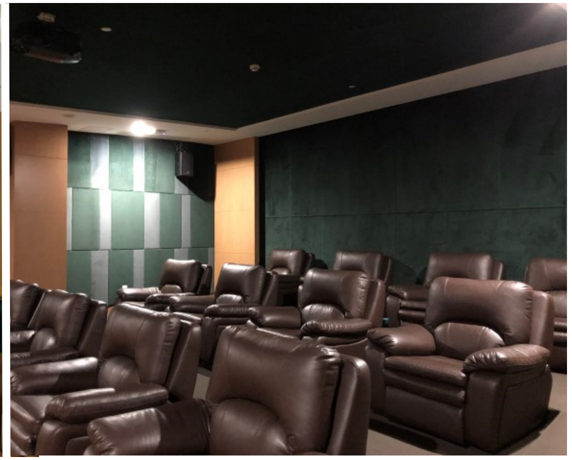
Activity Space

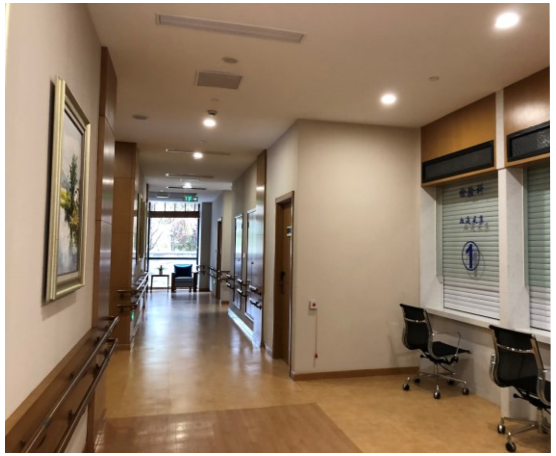
Corridor

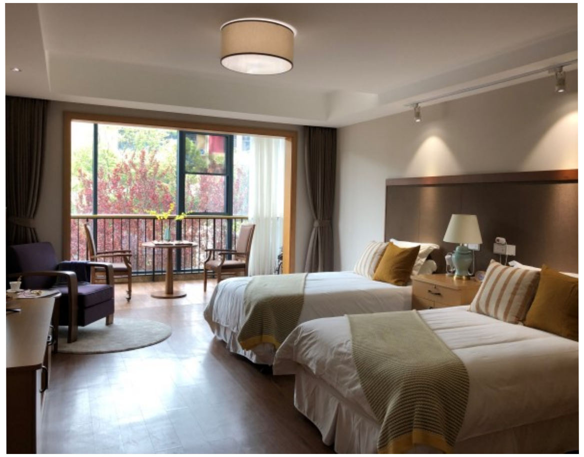
Resident Room

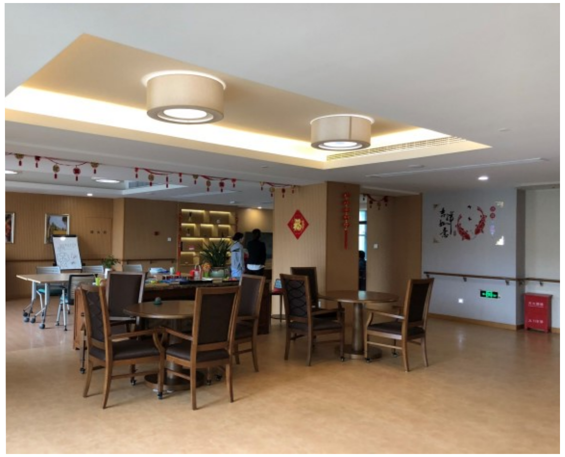
Living Room

Project 19	Location	Opening Year	Building Area	Number of Beds	Building Area per bed	Building Stories	Occupancy Rate	Types of Residents	Number of Staff	Construction Forms
	Beijing	2017	2300㎡	50	46.00 ㎡/beds	Ground 4	40.0%	Independent; Functional loss	Unknown	Renovation

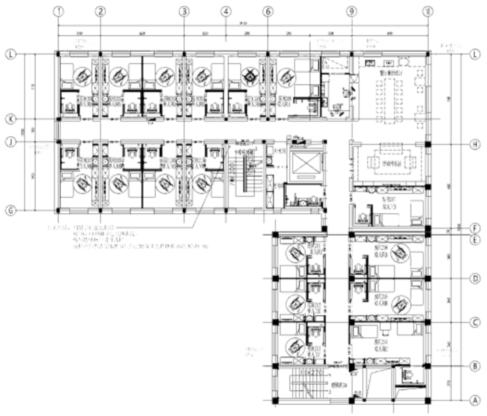

Standard Floor Plan

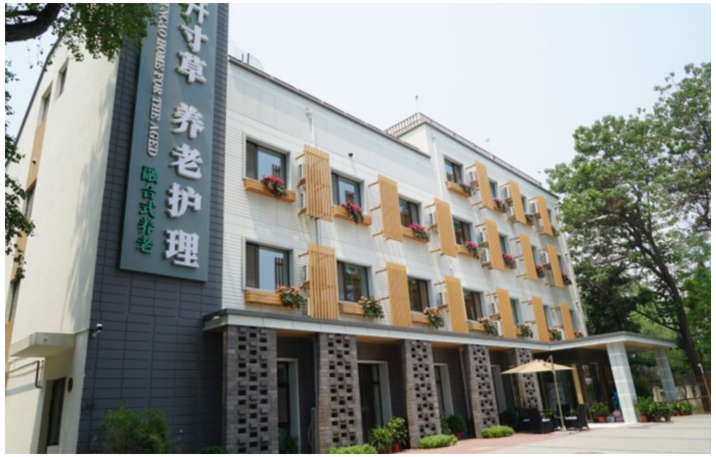

Building Exterior

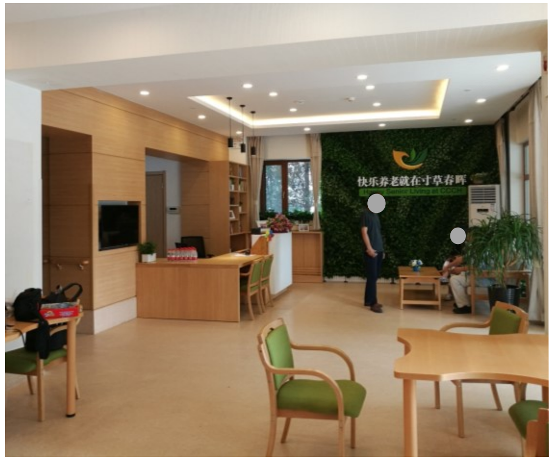

Entry Lobby

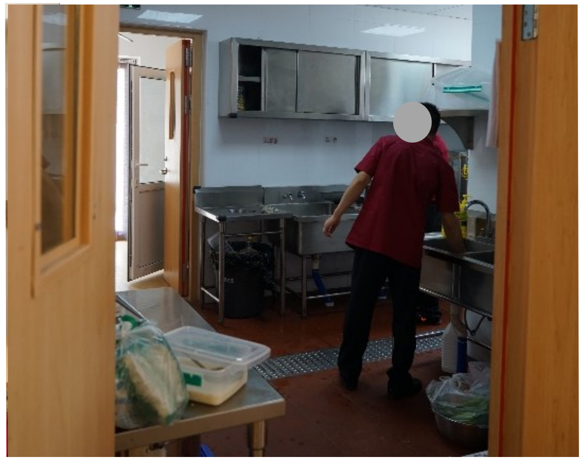

Central Kitchen

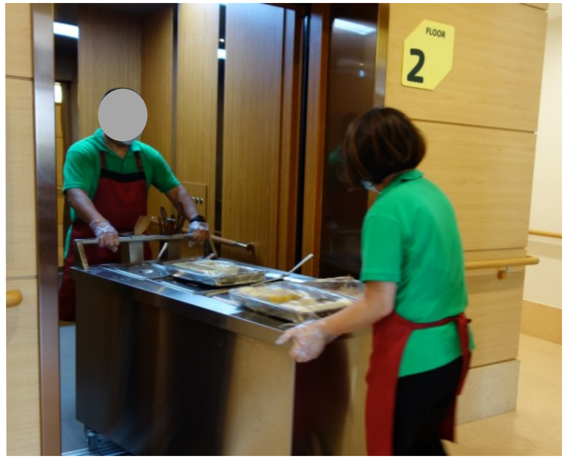

Elevator

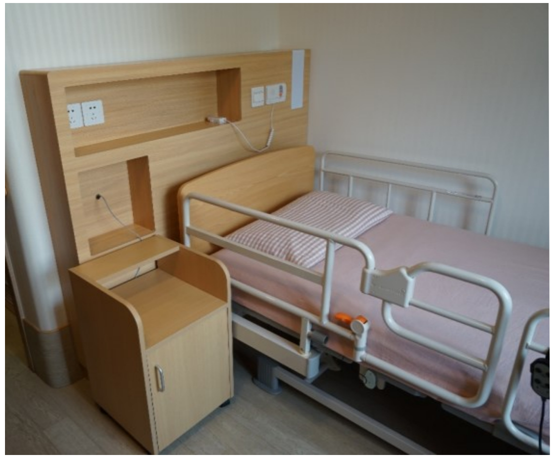

Resident Room

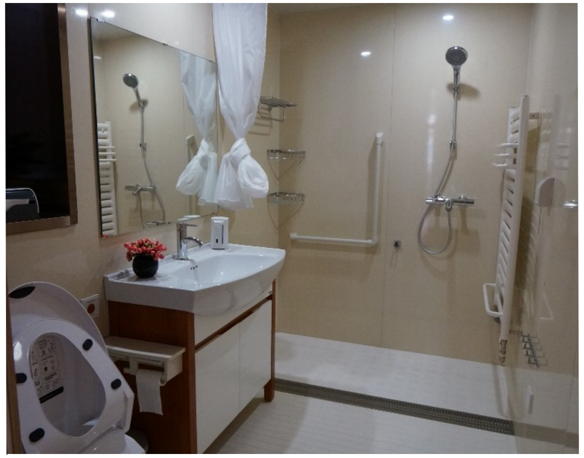

Public Bathroom

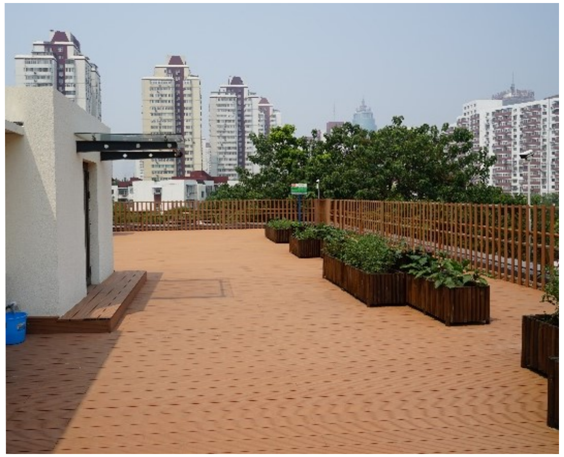

Outdoor Space

Project 20	Location	Opening Year	Building Area	Number of Beds	Building Area per bed	Building Stories	Occupancy Rate	Types of Residents	Number of Staff	Construction Forms
	Beijing	2018	1873㎡	47	39.85 ㎡/beds	Ground 1	95.7%	Independent; Functional loss; Dementia	Unknown	Renovation

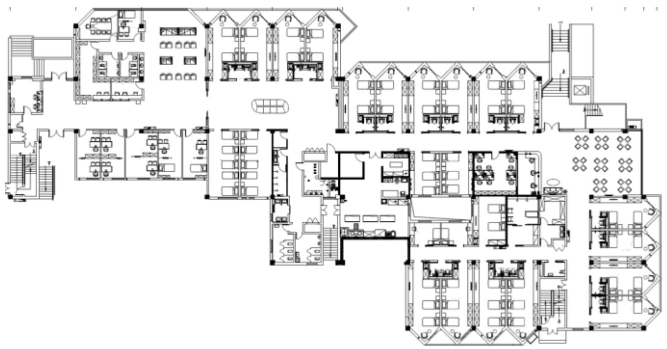
Standard Floor Plan

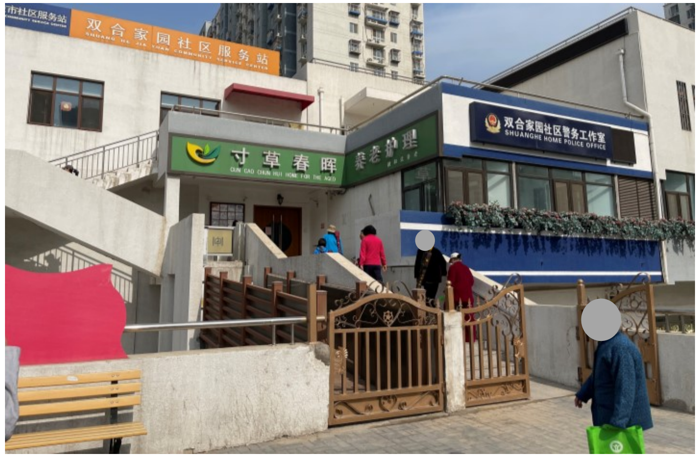

Building Exterior

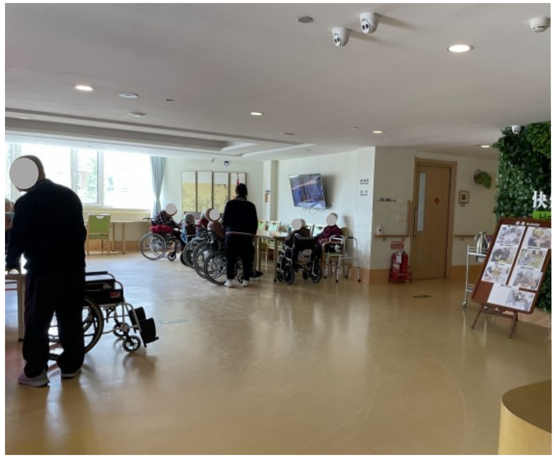
Entry Lobby

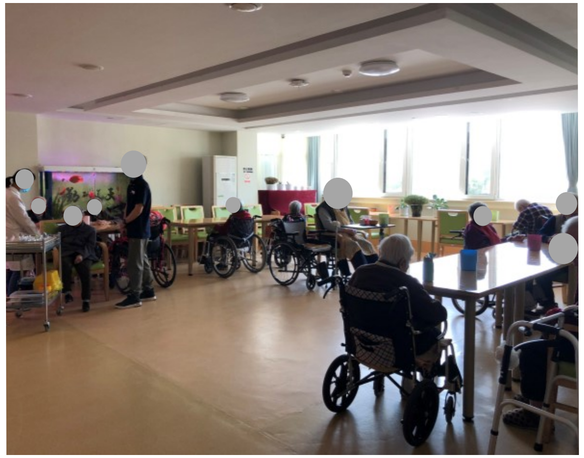
Living Room

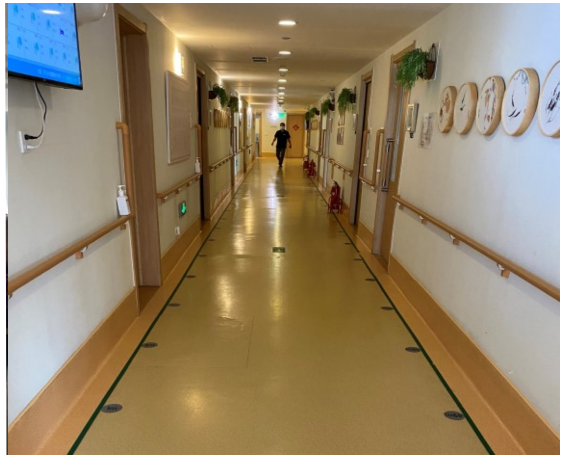
Corridor

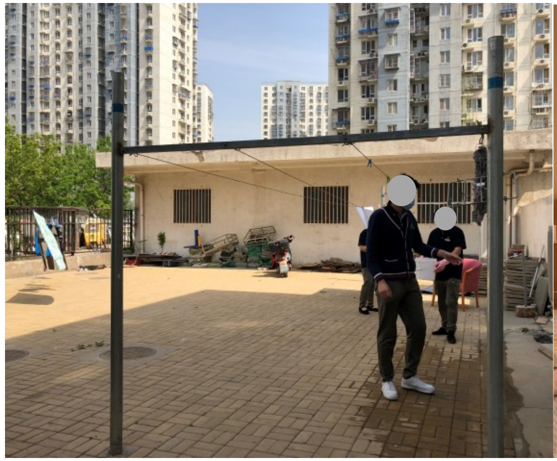
Outdoor Space

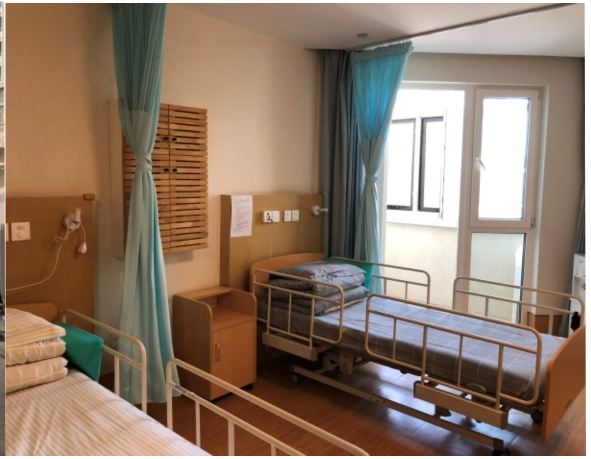
Resident Room

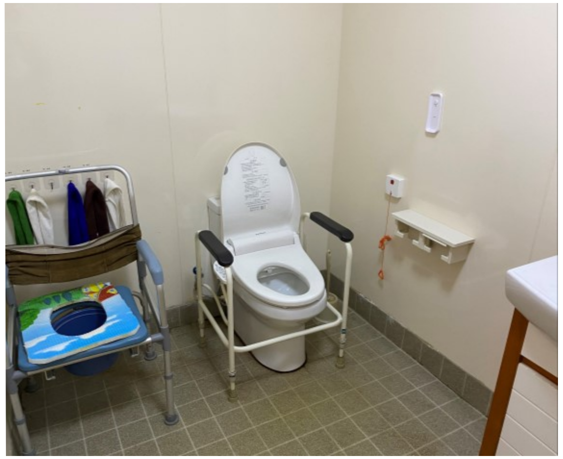
Toilet

Project 21

Location	Opening Year	Building Area	Number of Beds	Building Area per bed	Building Stories	Occupancy Rate	Types of Residents	Number of Staff	Construction Forms
Beijing	2018	3518㎡	82	42.90 ㎡/beds	Ground 3	92.7%	Independent; Functional loss; Dementia	40	Renovation

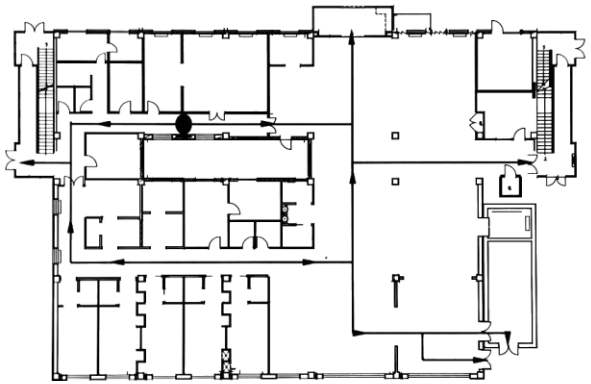

Standard Floor Plan

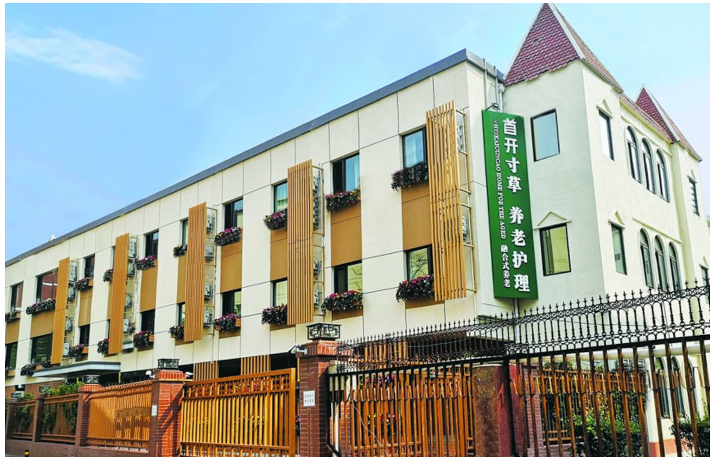

Building Exterior

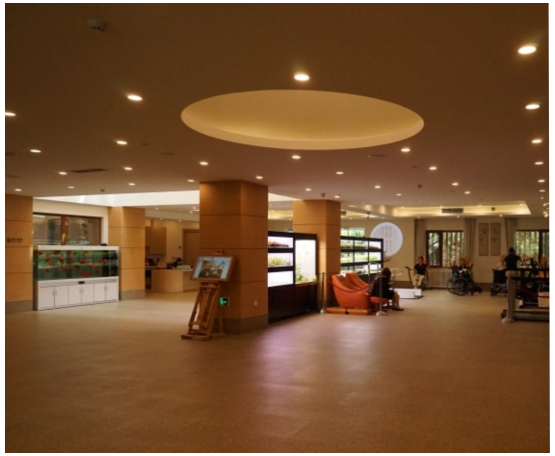

Entry Lobby

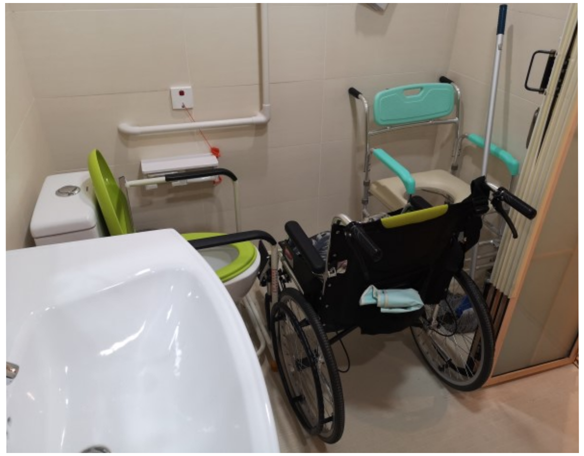

Public Bathroom

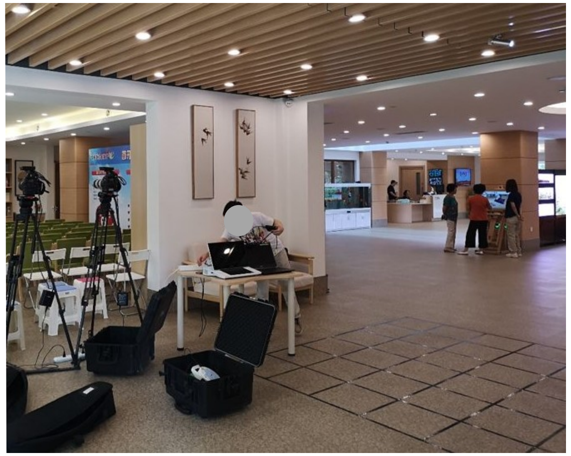

Multi-function Room

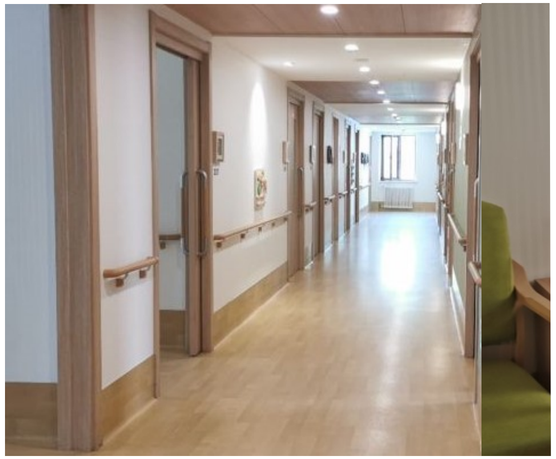

Corridor

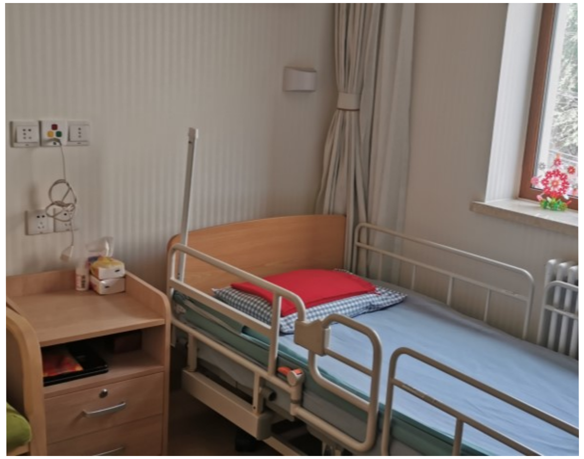

Resident Room

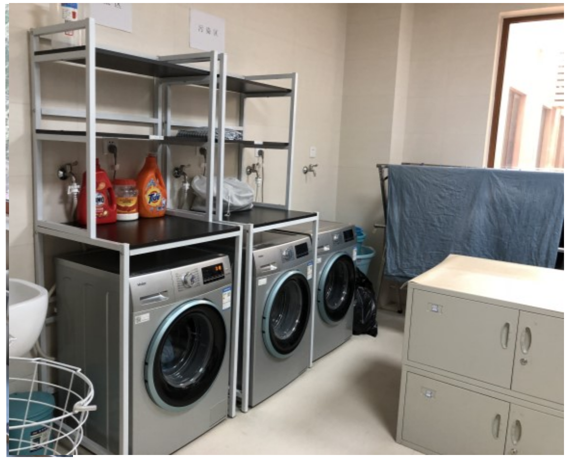

Laundry Space

Project 22	Location	Opening Year	Building Area	Number of Beds	Building Area per bed	Building Stories	Occupancy Rate	Types of Residents	Number of Staff	Construction Forms
	Beijing	2015	759㎡	28	27.10 ㎡/beds	Ground 2	75.0%	Independent; Functional loss	9	Renovation

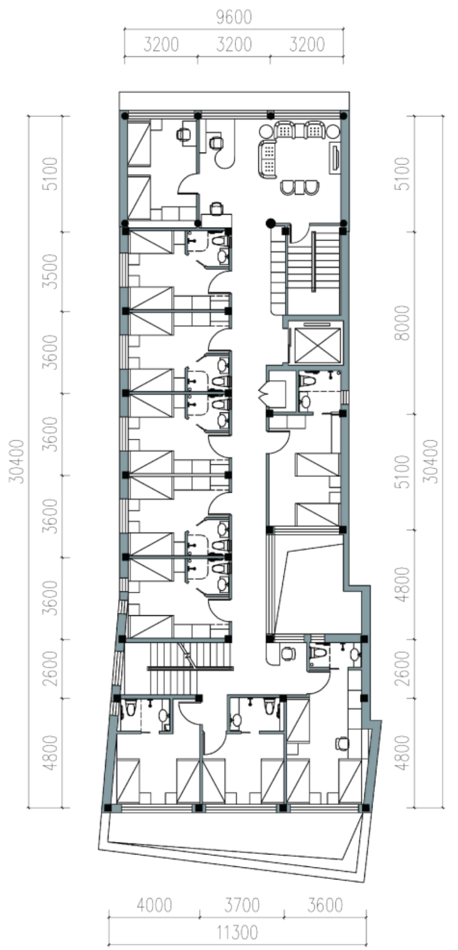

Second Floor Plan

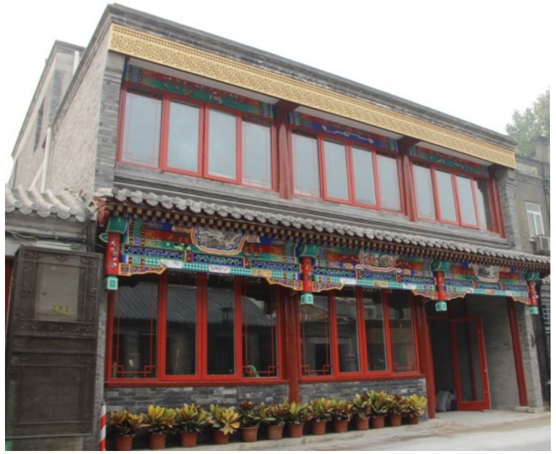

Building Exterior

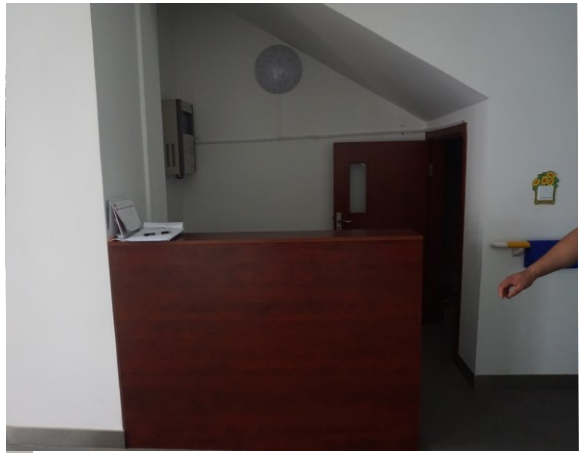

Nursing Station

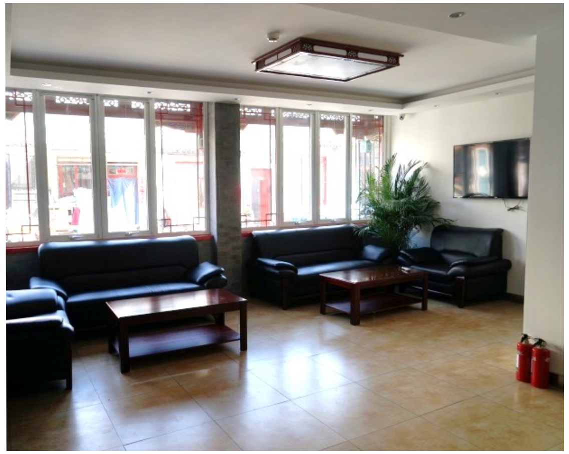

Living Room

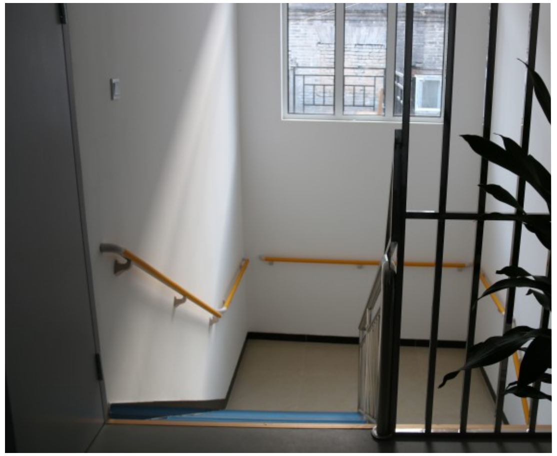

Staircase

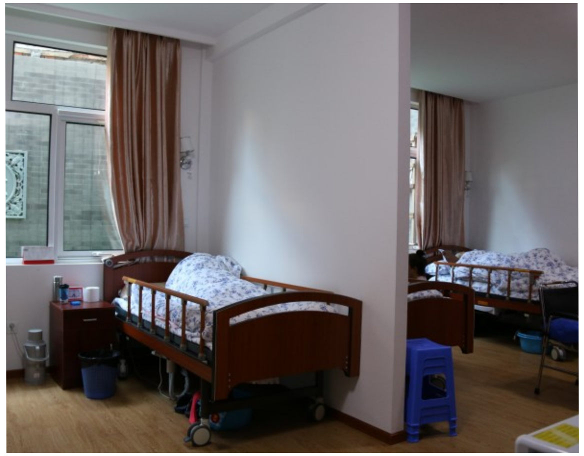

Resident Room

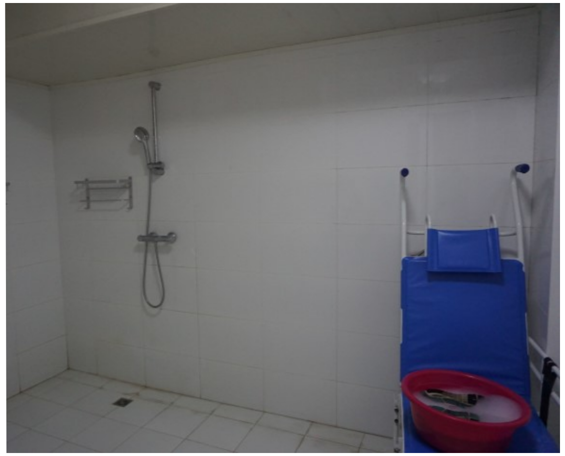

Public Bathroom

Project 23

Location	Opening Year	Building Area	Number of Beds	Building Area per bed	Building Stories	Occupancy Rate	Types of Residents	Number of Staff	Construction Forms
Guangzhou	2017	1250㎡	38	32.89 ㎡/beds	Ground 2	73.7%	Independent; Functional loss; Dementia	18	Renovation

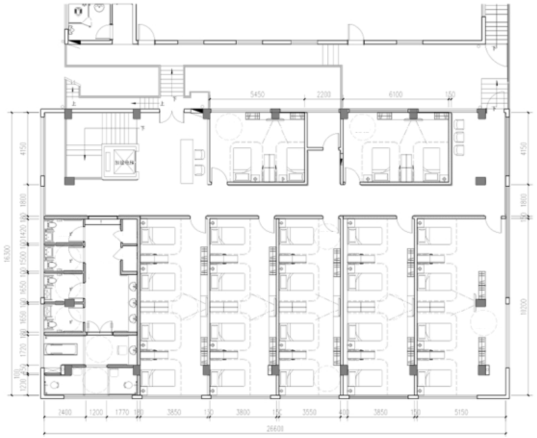
Second Floor Plan

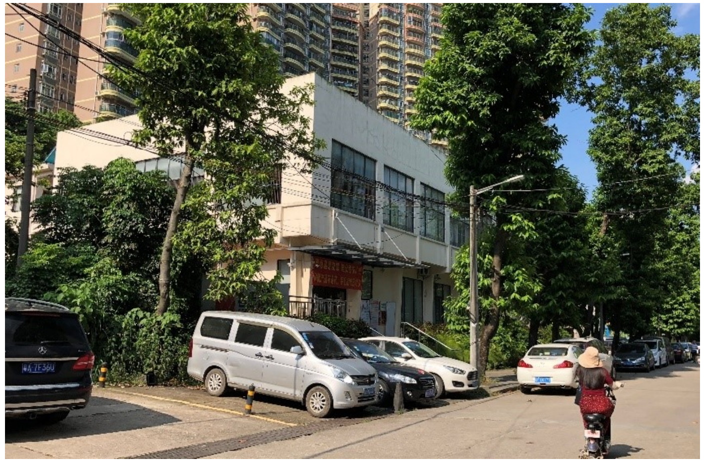
Building Exterior

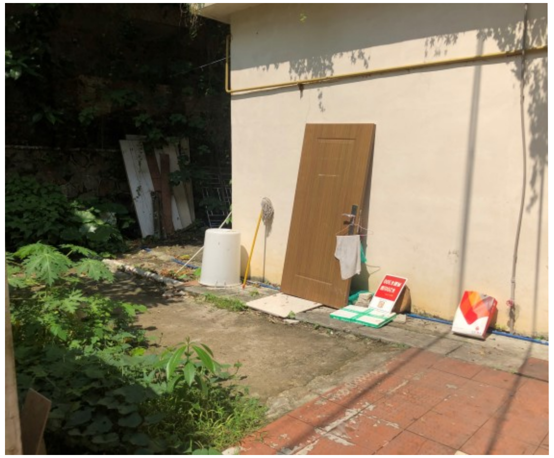
Outdoor Space

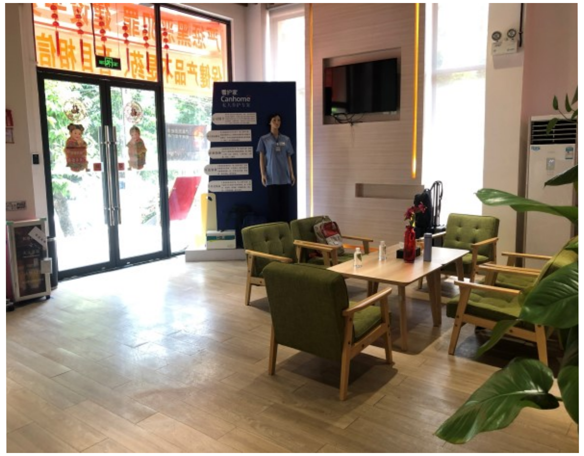
Entry Lobby

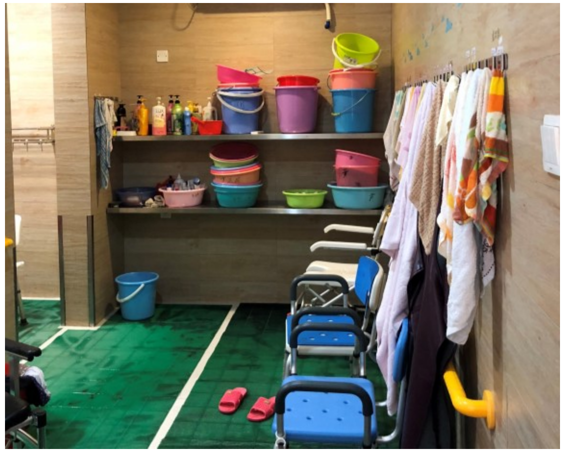
Public Bathroom

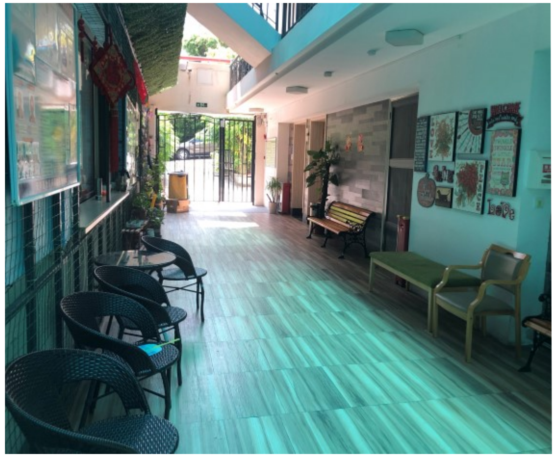
Corridor

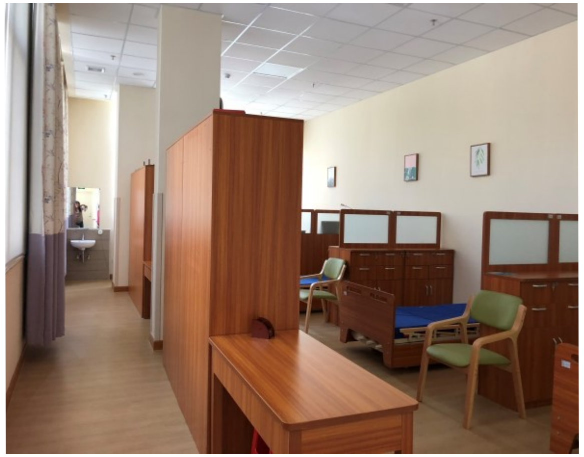
Resident Room

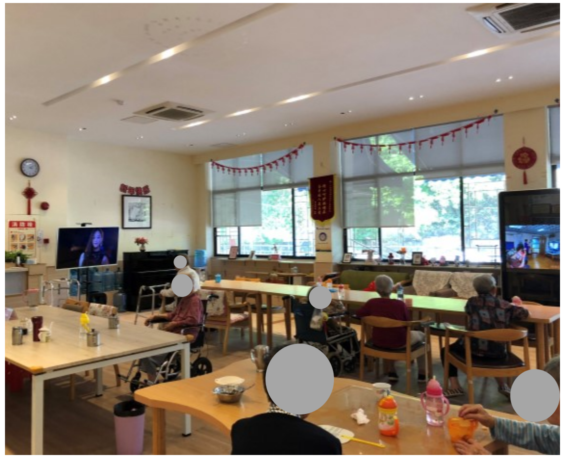
Dining Space

Project 24	Location	Opening Year	Building Area	Number of Beds	Building Area per bed	Building Stories	Occupancy Rate	Types of Residents	Number of Staff	Construction Forms
	Guangzhou	2015	1253㎡	48	26.10 ㎡/beds	Ground 7	100.0%	Independent; Functional loss; Dementia	21	Renovation

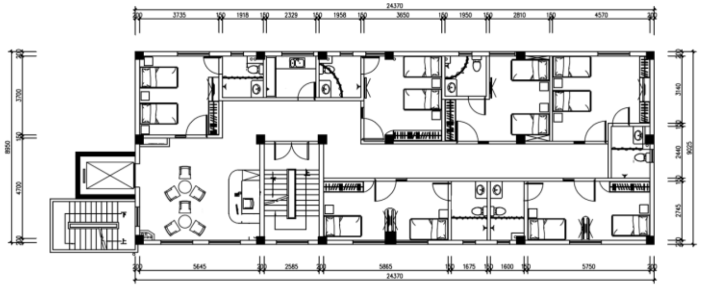

Standard Floor Plan

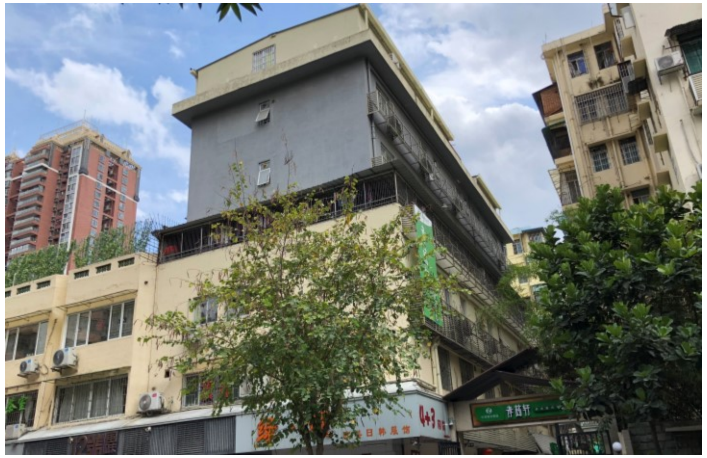

Building Exterior

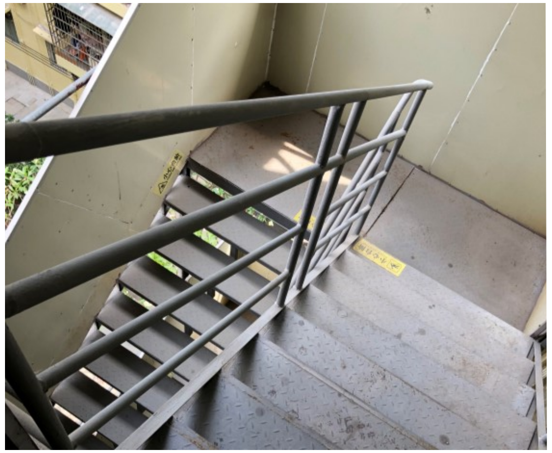

Staircase

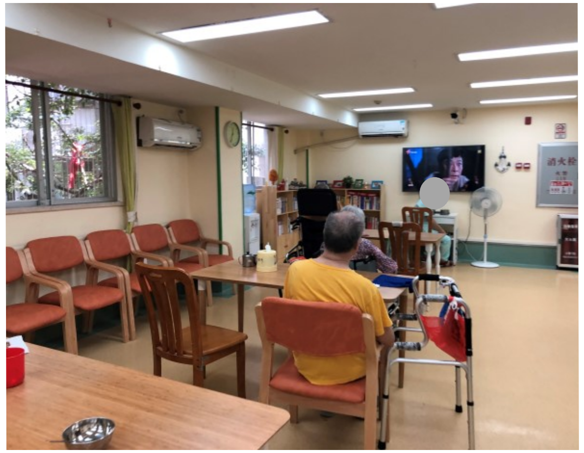

Living Room

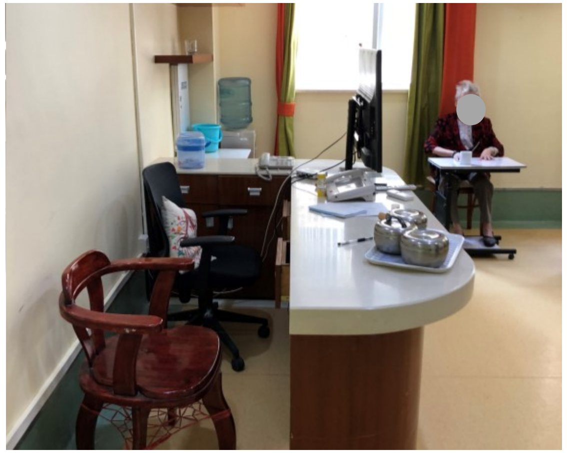

Nursing Station

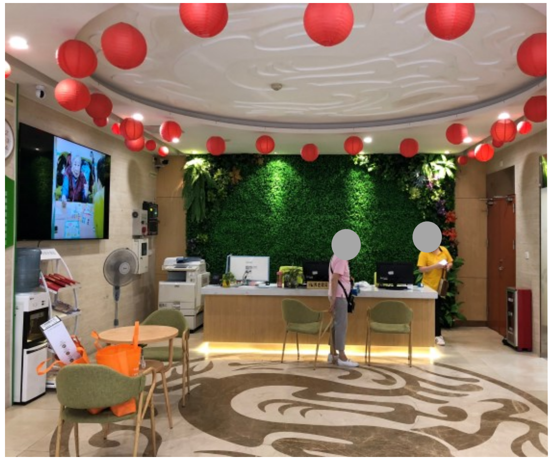

Entry Lobby

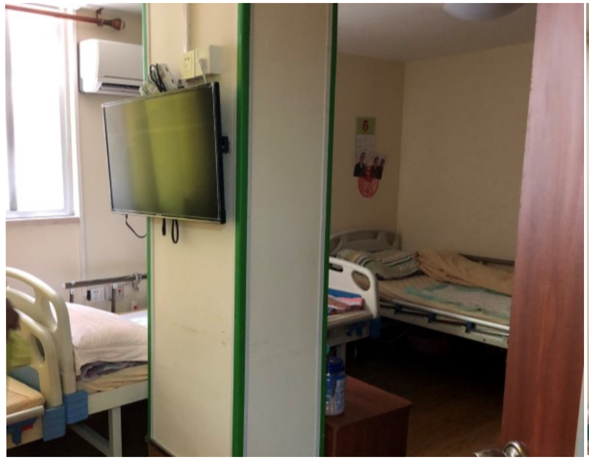

Resident Room

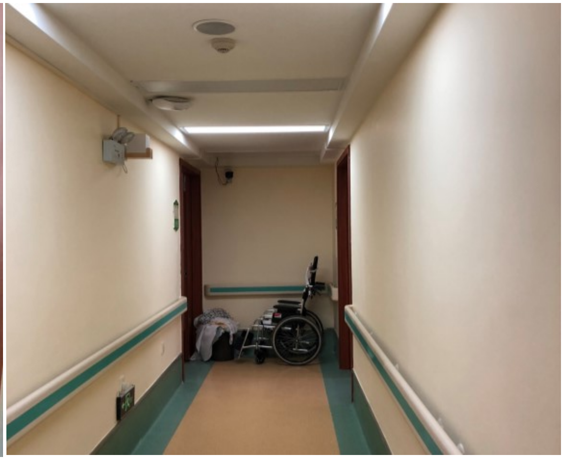

Corridor

Project 25	Location	Opening Year	Building Area	Number of Beds	Building Area per bed	Building Stories	Occupancy Rate	Types of Residents	Number of Staff	Construction Forms
	Shenzhen	2015	1010㎡	60	16.83 ㎡/beds	Ground 3	100.0%	Independent; Functional loss; Dementia	17	Renovation

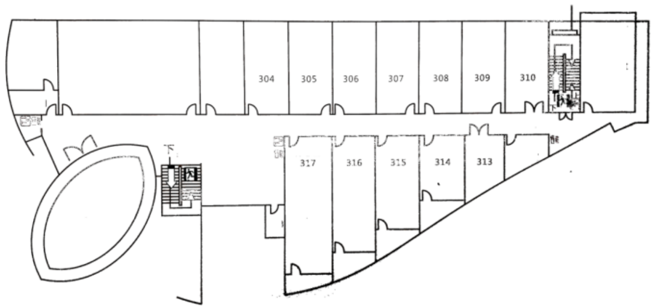

Third Floor Plan

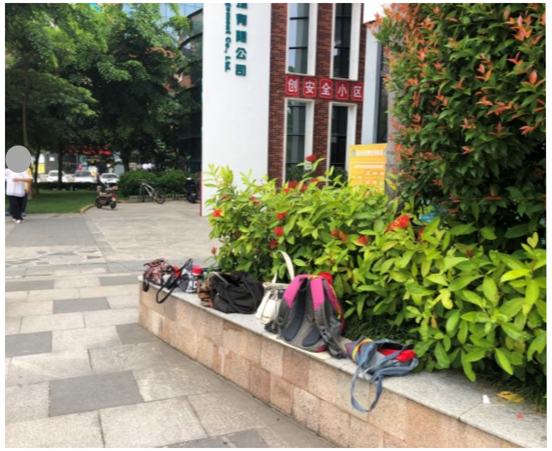

Outdoor Space

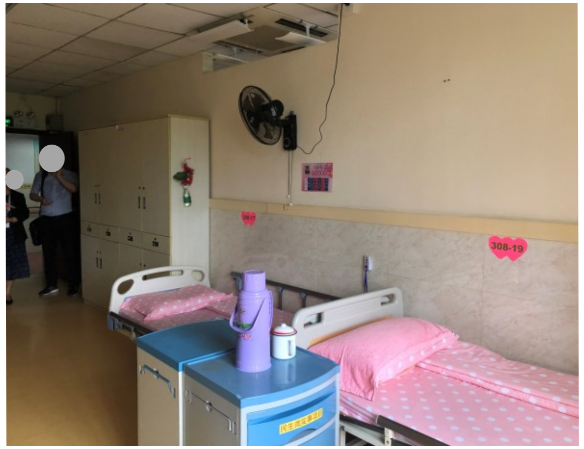

Resident Room

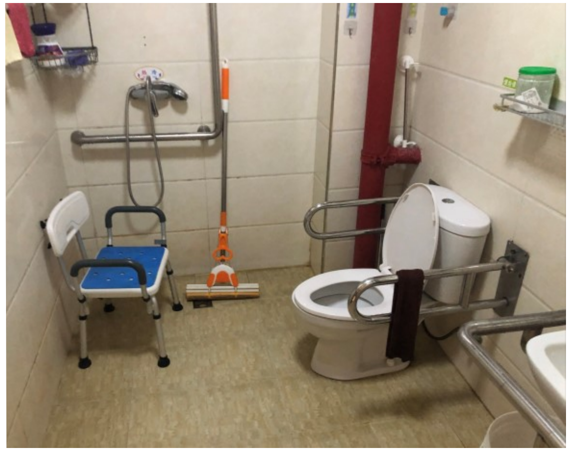

Toilet

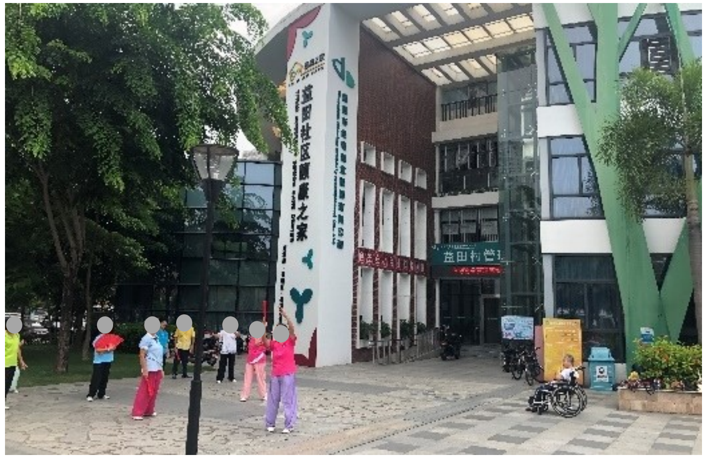

Building Exterior

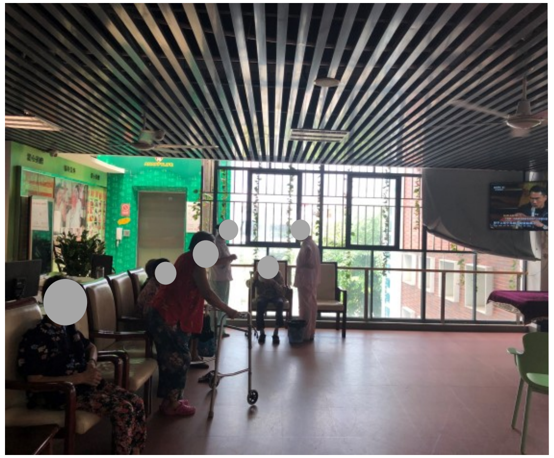

Living Room

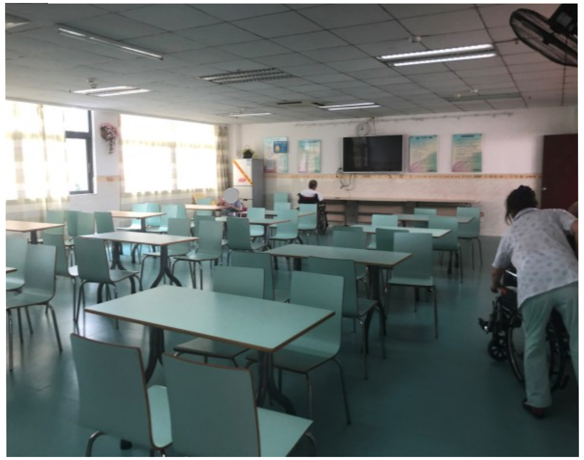

Dining Space

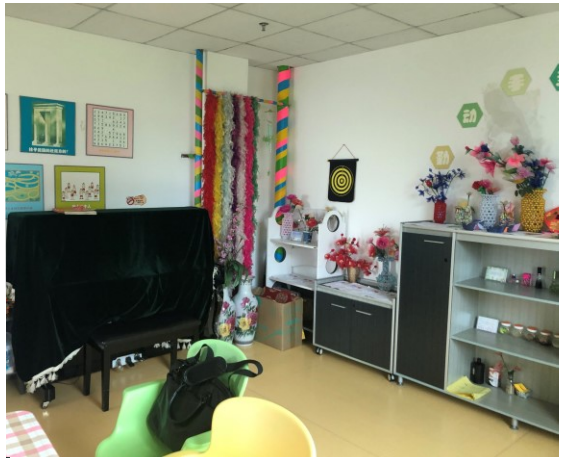

Multi-function Room

Project 26	Location	Opening Year	Building Area	Number of Beds	Building Area per bed	Building Stories	Occupancy Rate	Types of Residents	Number of Staff	Construction Forms
	Nanjing	2017	2247㎡	83	27.07 ㎡/beds	Ground 4 Underground 1	83.1%	Independent; Functional loss; Dementia	45	Renovation

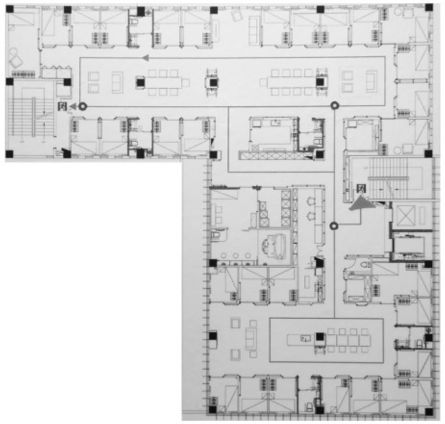

Standard Floor Plan

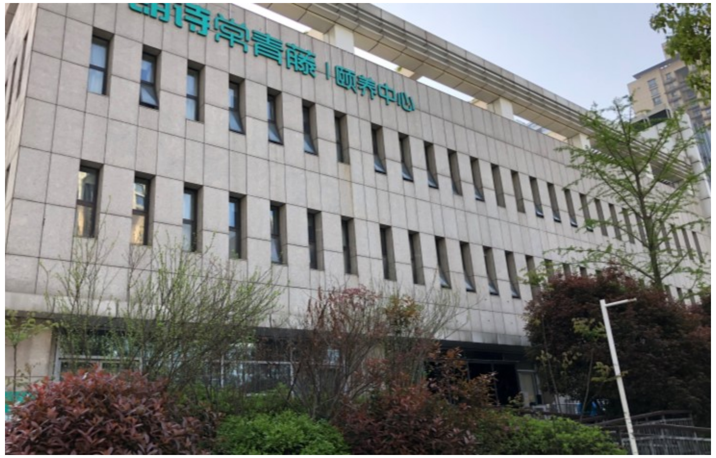

Building Exterior

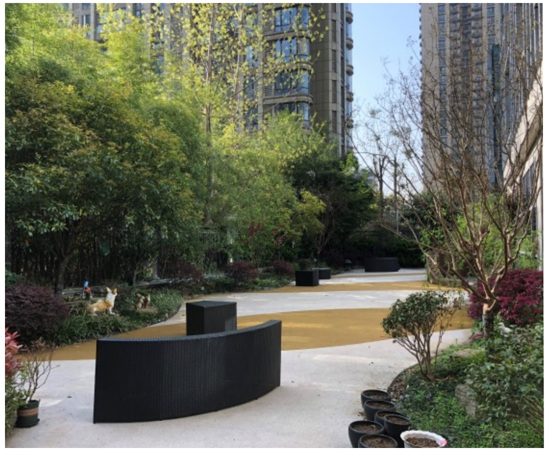

Outdoor Space

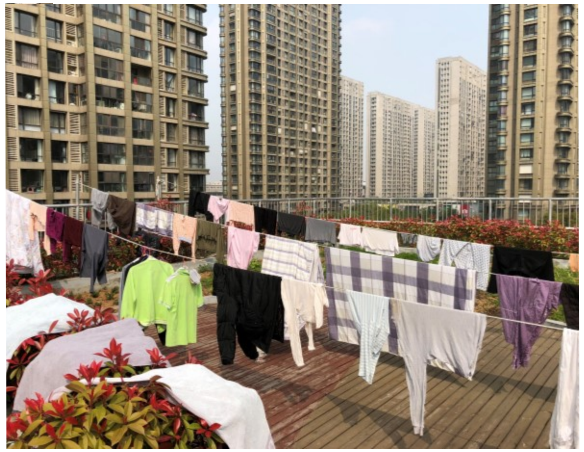

Outdoor Terrace

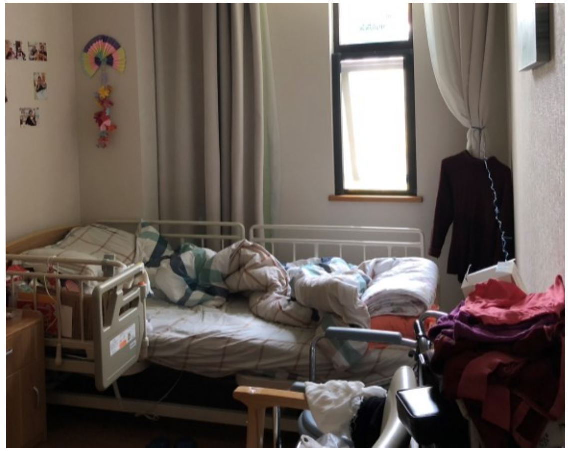

Resident Room

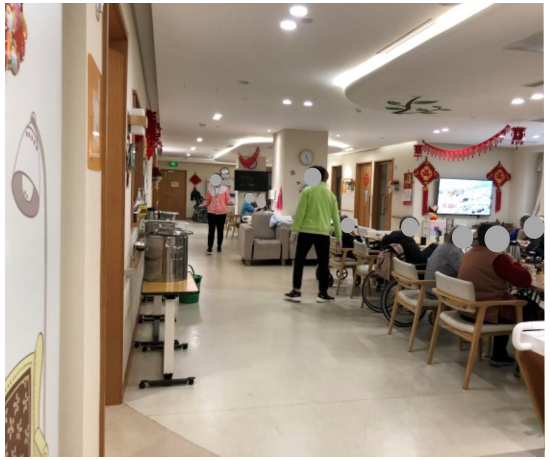

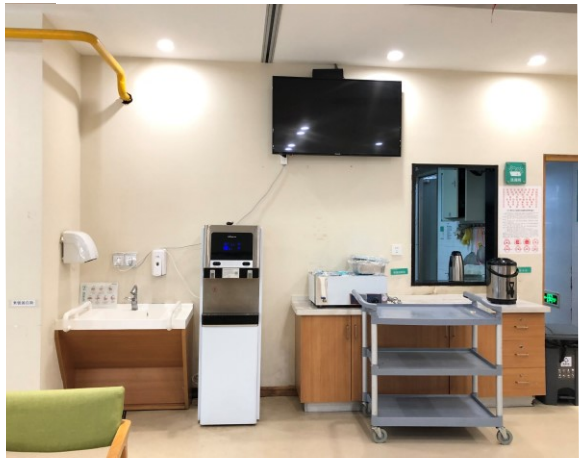

Living Room

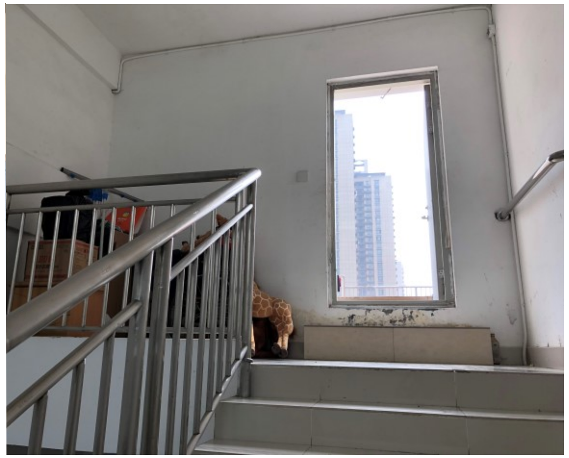

Staircase

Project	Location	Opening Year	Building Area	Number of Beds	Building Area per bed	Building Stories	Occupancy Rate	Types of Residents	Number of Staff	Construction Forms
Project 27	Nanjing	2018	6588㎡	164	40.17 ㎡/beds	Ground 2	82.3%	Independent; Functional loss; Dementia	Unknown	Renovation

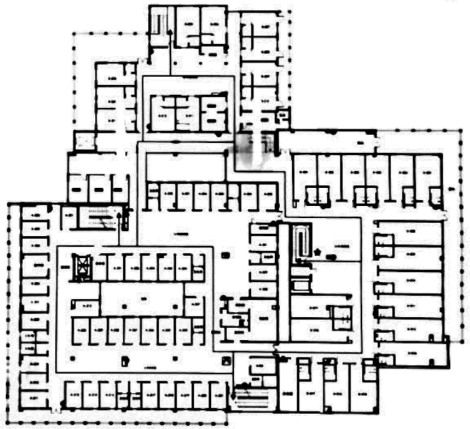

Second Floor Plan

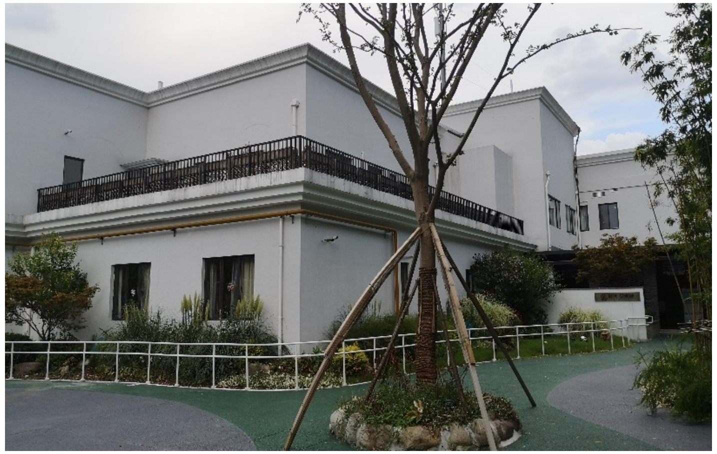

Building Exterior

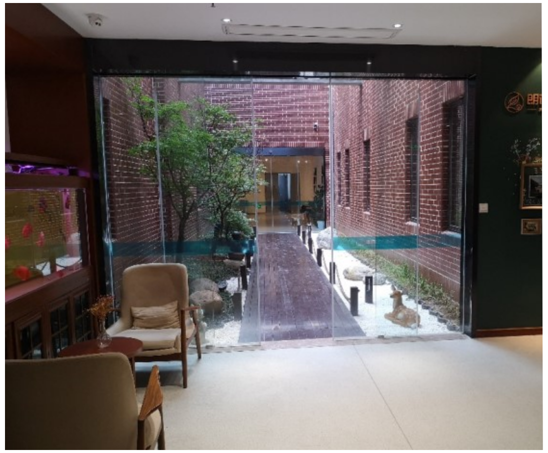

Entry Lobby

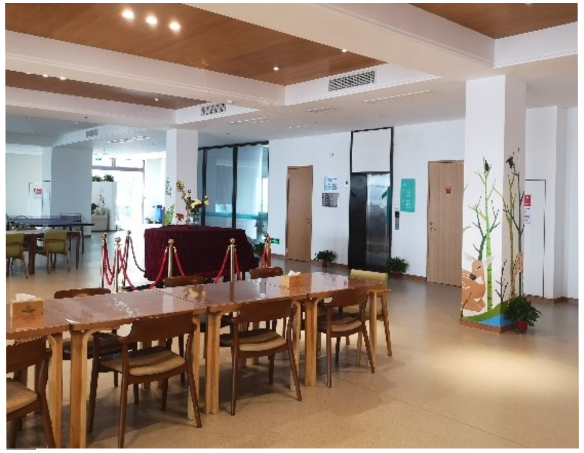

Dining Space

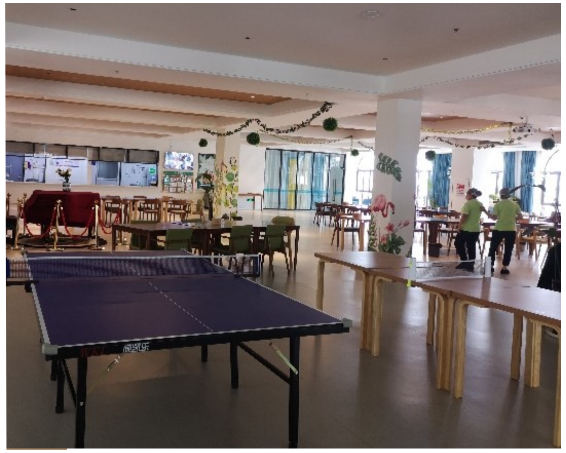

Activity Space

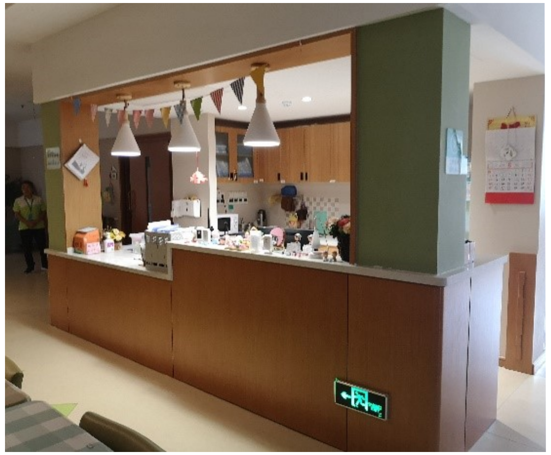

Nursing Station

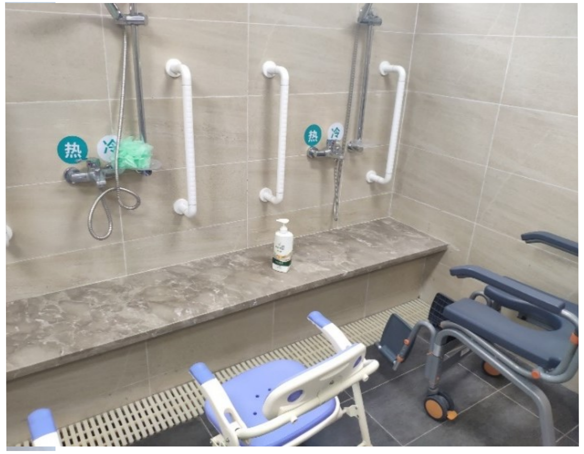

Public Bathroom

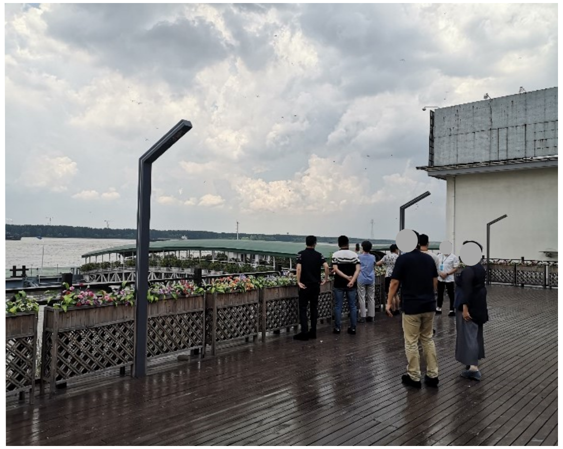

Outdoor Terrace

Project	Location	Opening Year	Building Area	Number of Beds	Building Area per bed	Building Stories	Occupancy Rate	Types of Residents	Number of Staff	Construction Forms
Project 28	Nanjing	2016	1650㎡	48	34.38 ㎡/beds	Ground 3	85.4%	Independent; Functional loss; Dementia	Unknown	Renovation

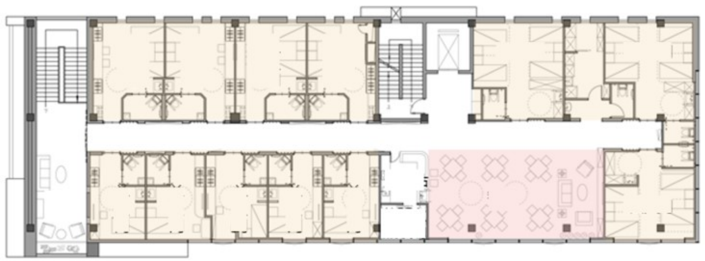

Standard Floor Plan

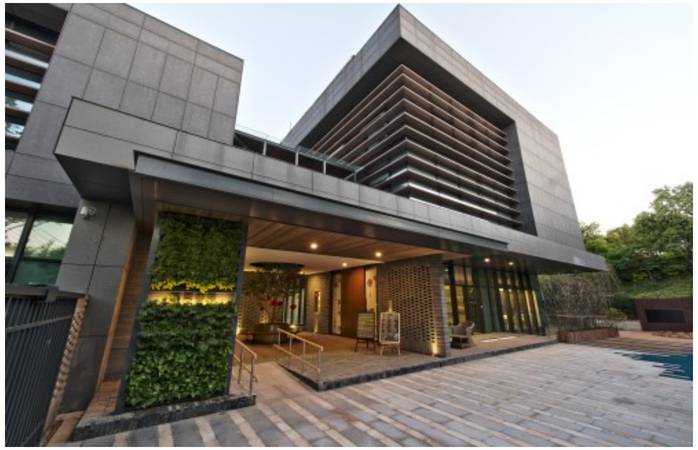

Building Exterior

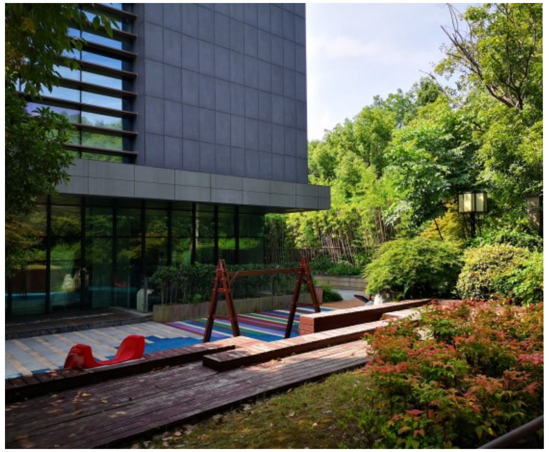

Outdoor Space

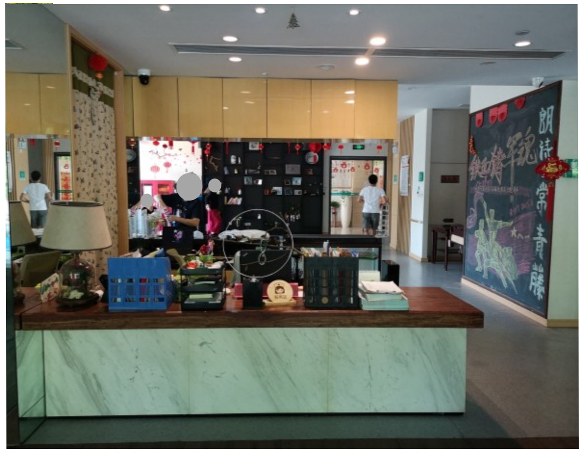

Entry Lobby

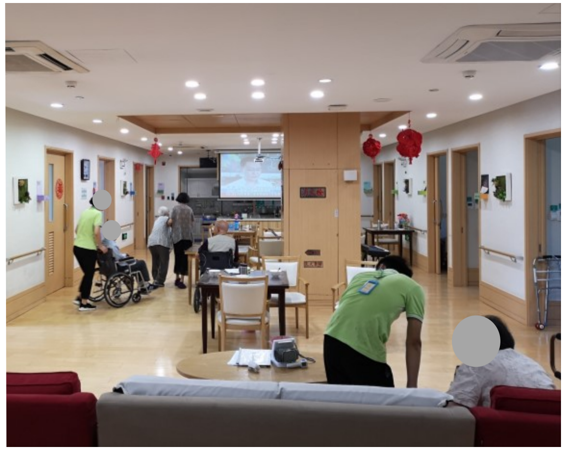

Living Room

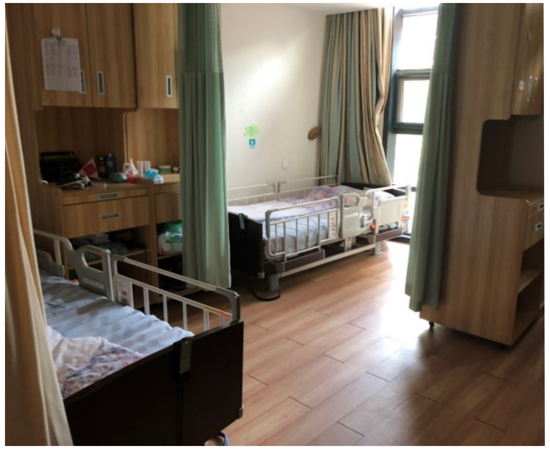

Resident Room

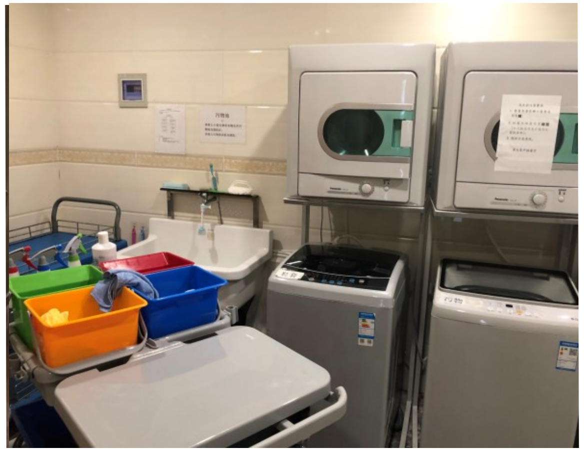

Laundry Space

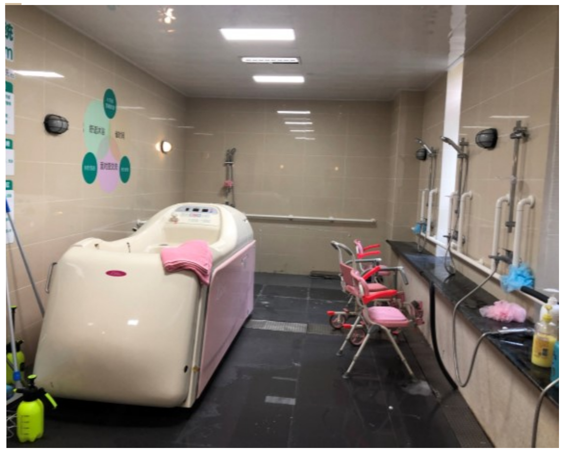

Public Bathroom

Project 29

Location	Opening Year	Building Area	Number of Beds	Building Area per bed	Building Stories	Occupancy Rate	Types of Residents	Number of Staff	Construction Forms
Shanghai	2020	2800㎡	85	32.94 ㎡/beds	Ground 4	23.5%	Independent; Functional loss; Dementia	20	Newly Built

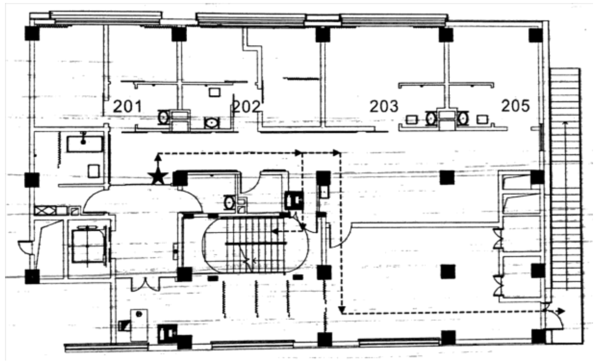

Standard Floor Plan

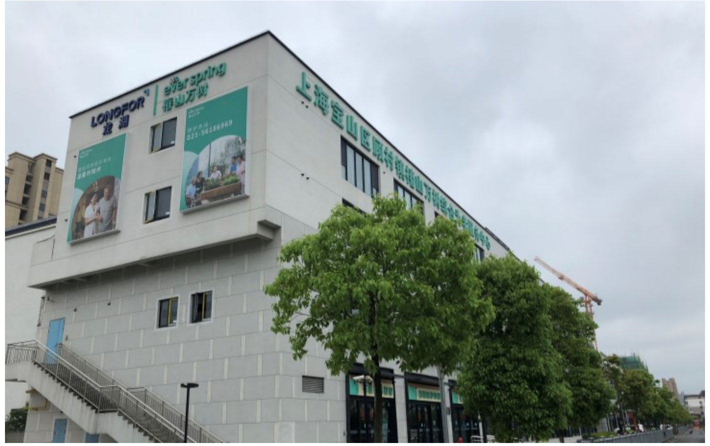

Building Exterior

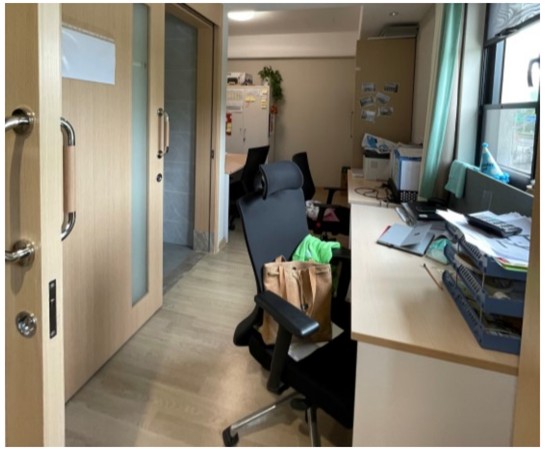
Staff Working Space

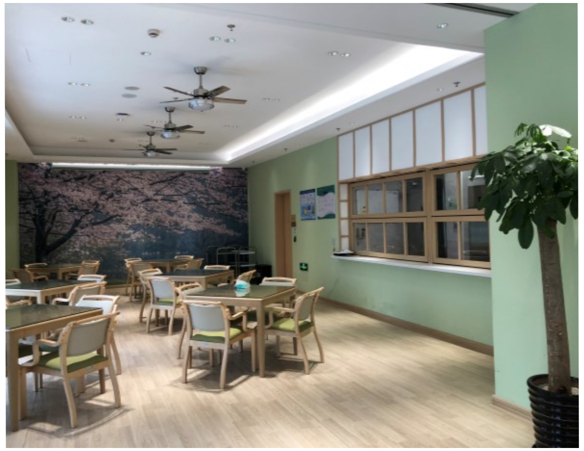
Dining Space

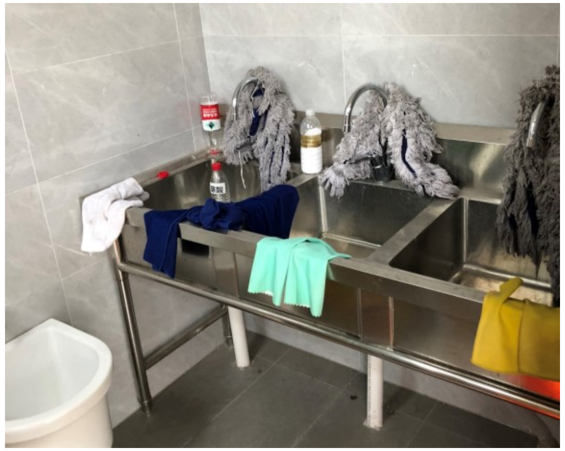
Cleaning Space

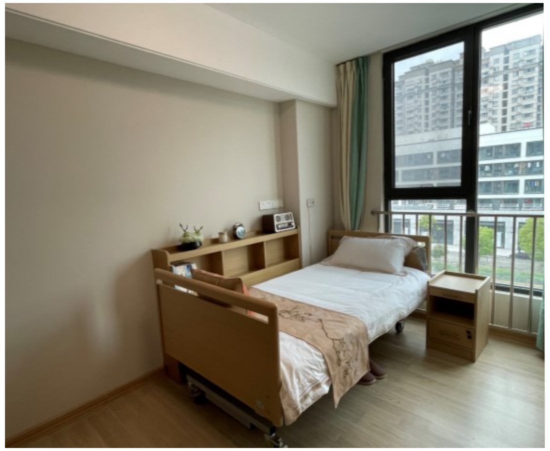
Resident Room

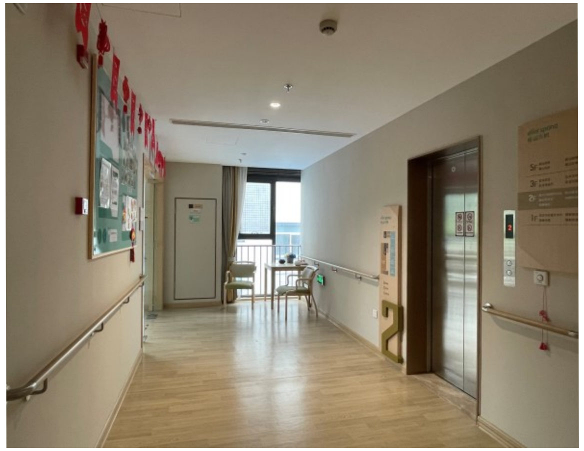
Corridor

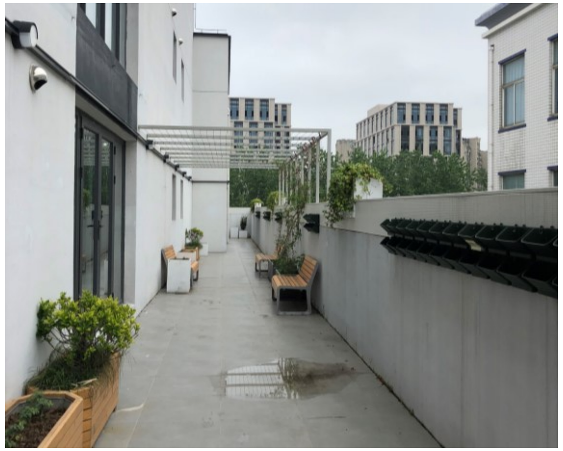
Outdoor Space

Project	Location	Opening Year	Building Area	Number of Beds	Building Area per bed	Building Stories	Occupancy Rate	Types of Residents	Number of Staff	Construction Forms
Project 30	Chengdu	2016	3588㎡	110	32.62 ㎡/beds	Ground 6	81.8%	Independent; Functional loss	22	Renovation

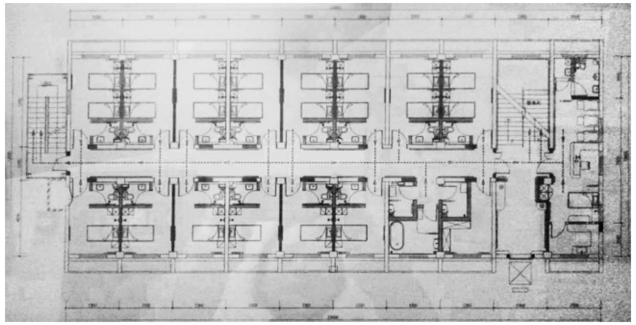

Standard Floor Plan

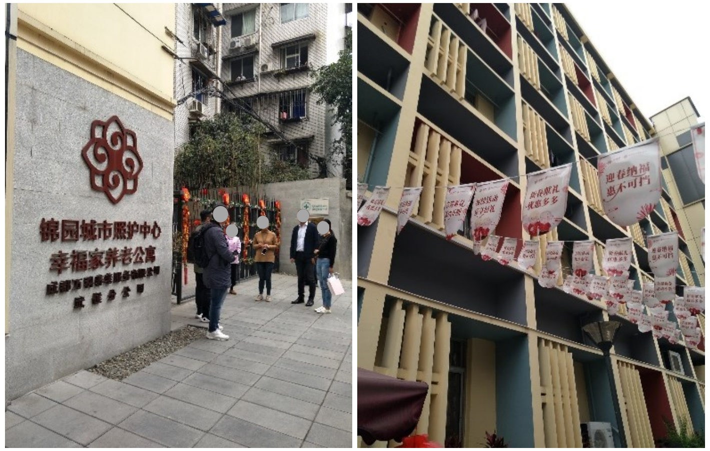

Building Exterior

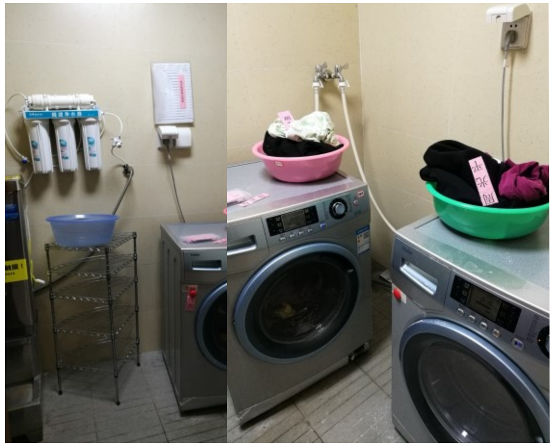

Laundry Space

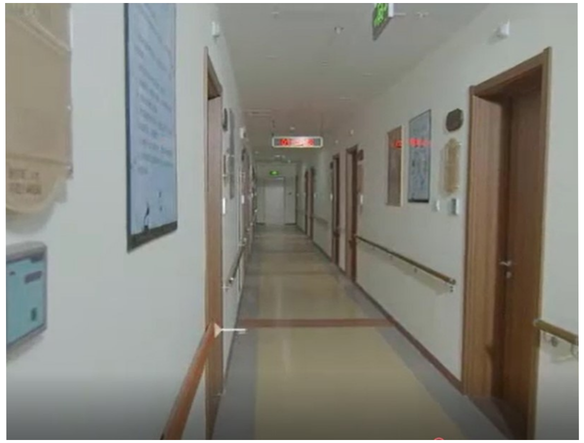

Corridor

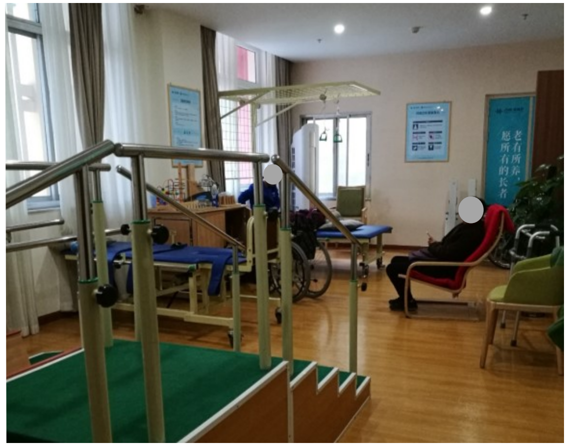

Rehabilitation Space

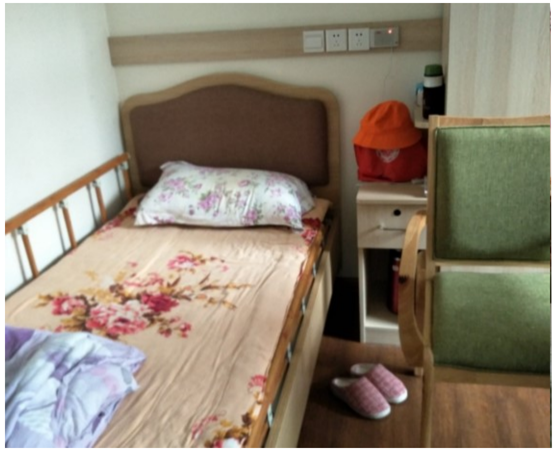

Resident Room

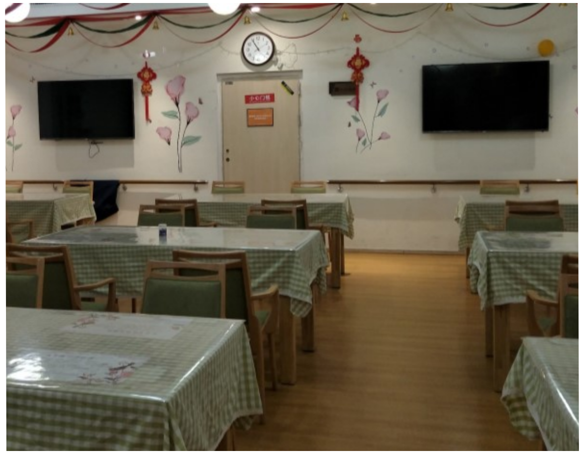

Dining Space

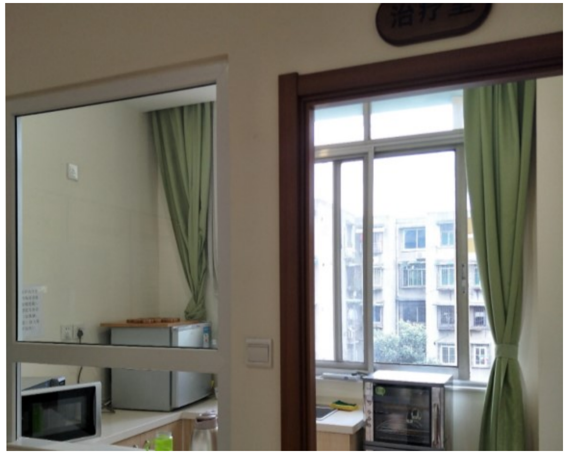

Nursing Station

Project 31

Location	Opening Year	Building Area	Number of Beds	Building Area per bed	Building Stories	Occupancy Rate	Types of Residents	Number of Staff	Construction Forms
Guangzhou	2010	6527 m^2	189	34.53 m^2/beds	Ground 8	Unknown	Independent; Functional loss; Dementia	Unknown	Newly Built

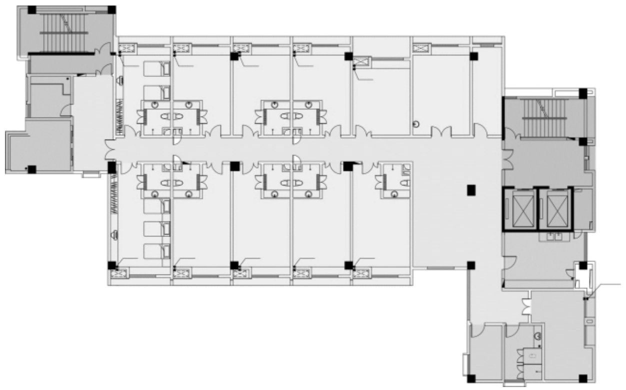

Standard Floor Plan

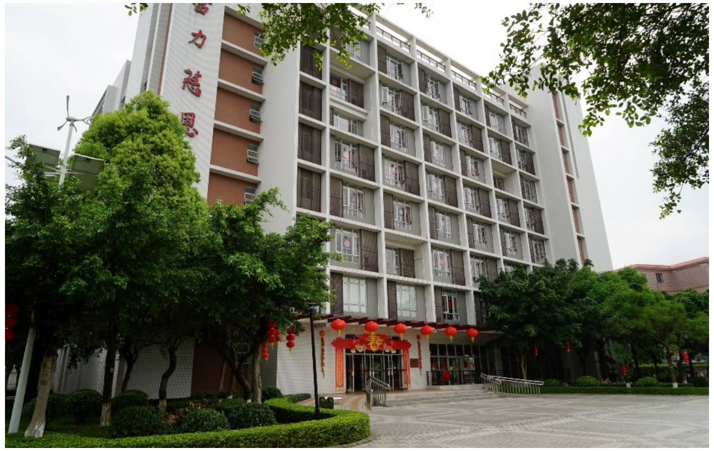

Building Exterior

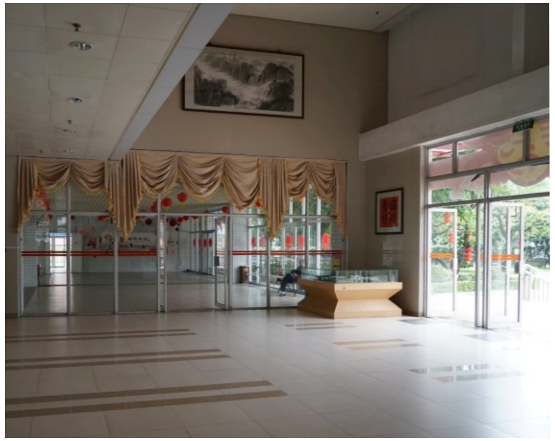

Entry Lobby

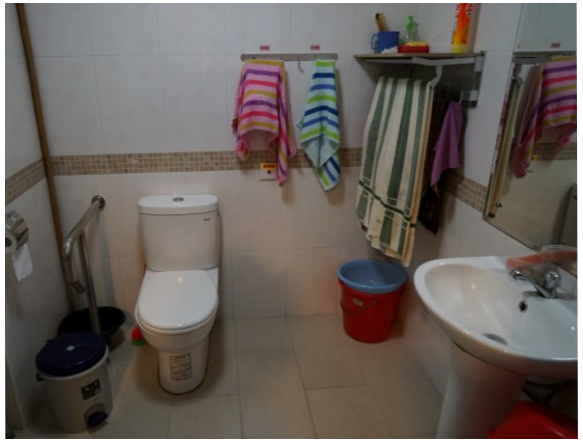

Toilet

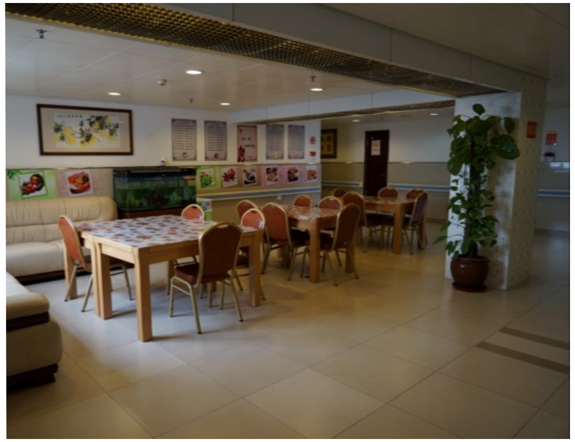

Dining Space

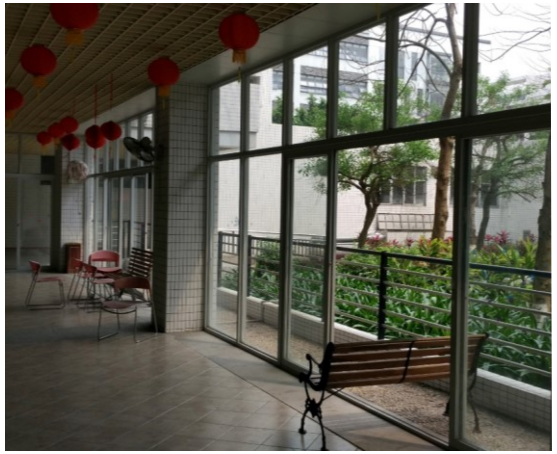

Activity Space

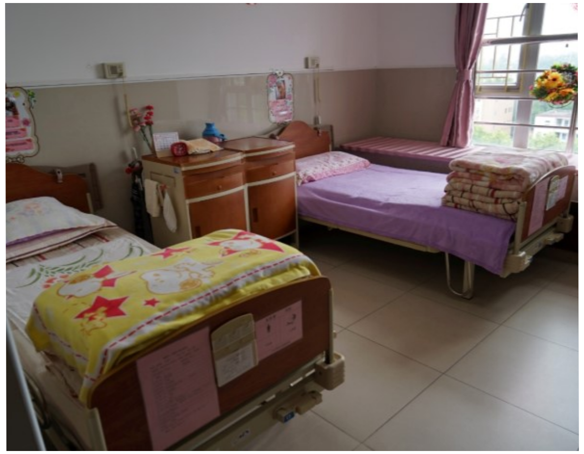

Resident Room

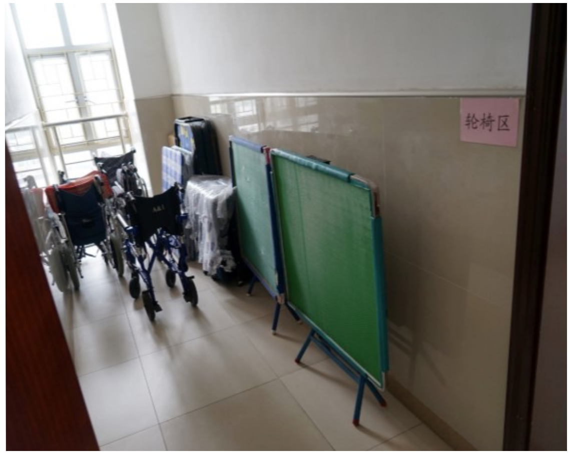

Public Storage Space

Project 32	Location	Opening Year	Building Area	Number of Beds	Building Area per bed	Building Stories	Occupancy Rate	Types of Residents	Number of Staff	Construction Forms
	Shenzhen	2015	31469㎡	791	39.78 ㎡/beds	Ground 10 Underground 1	44.9%	Independent; Functional loss; Dementia	175	Newly Built

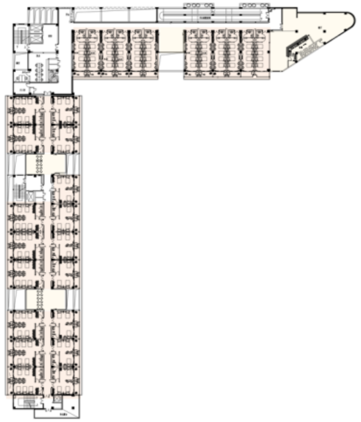

Standard Floor Plan

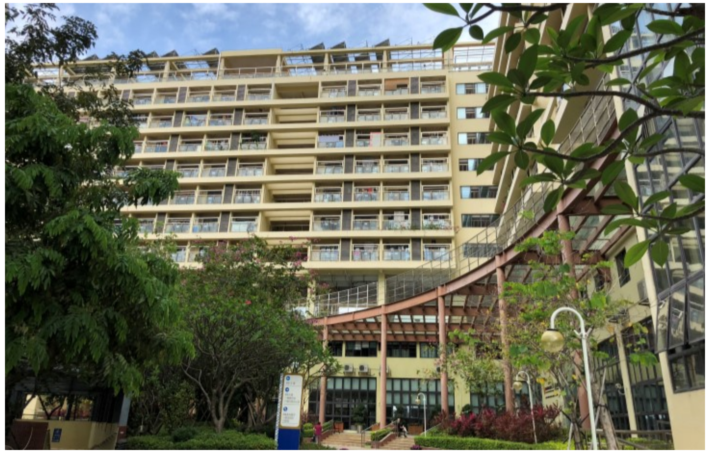

Building Exterior

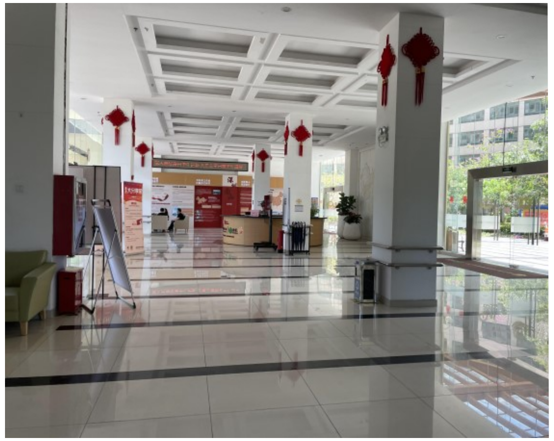

Entry Lobby

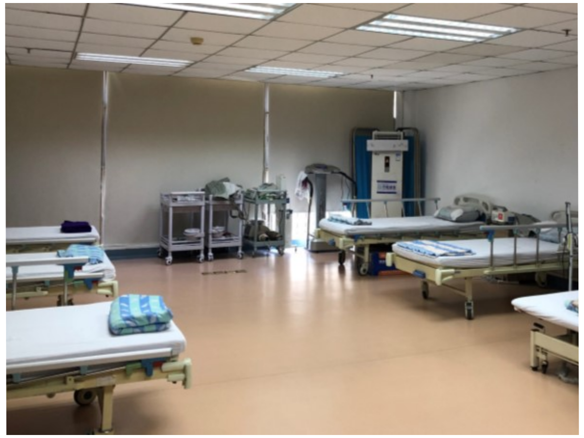

Rehabilitation Space

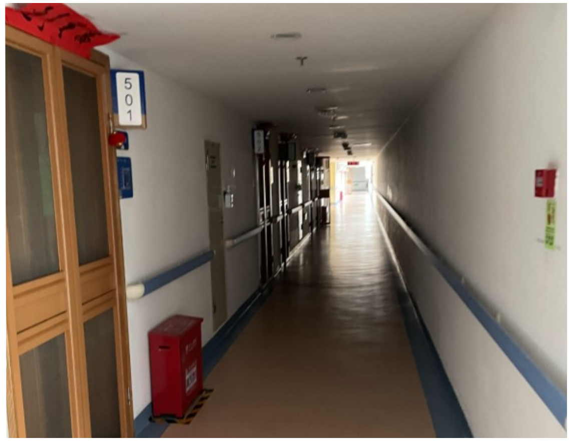

Corridor

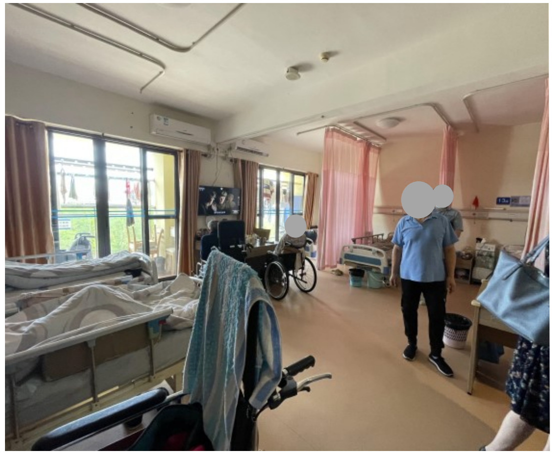

Resident Room

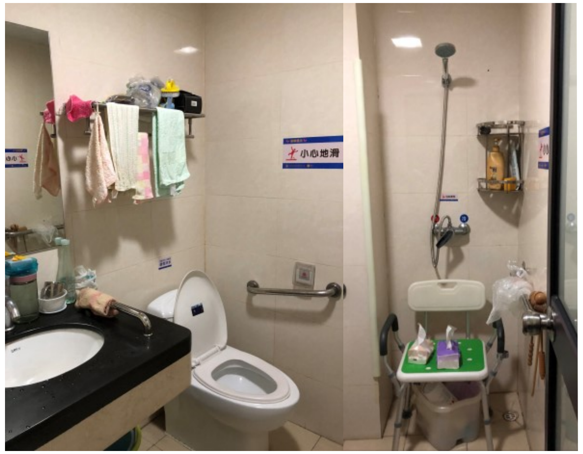

Toilet

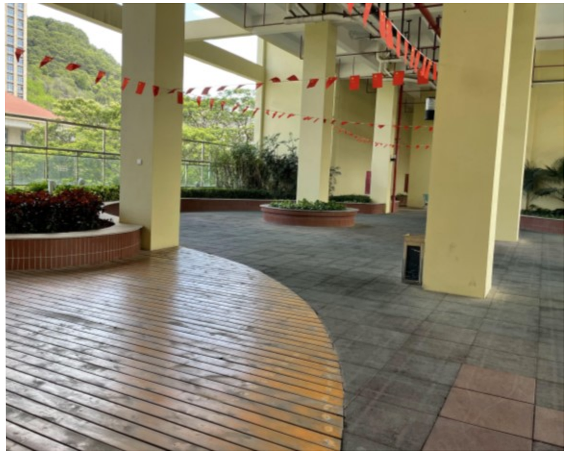

Rooftop Garden

Project 33

Location	Opening Year	Building Area	Number of Beds	Building Area per bed	Building Stories	Occupancy Rate	Types of Residents	Number of Staff	Construction Forms
Nanjing	2019	16316㎡	316	51.63 ㎡/beds	Ground 6 Underground 1	24.7%	Independent; Functional loss; Dementia	42	Newly Built

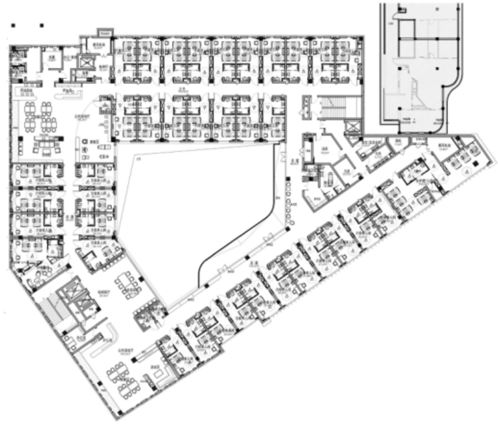
Standard Floor Plan

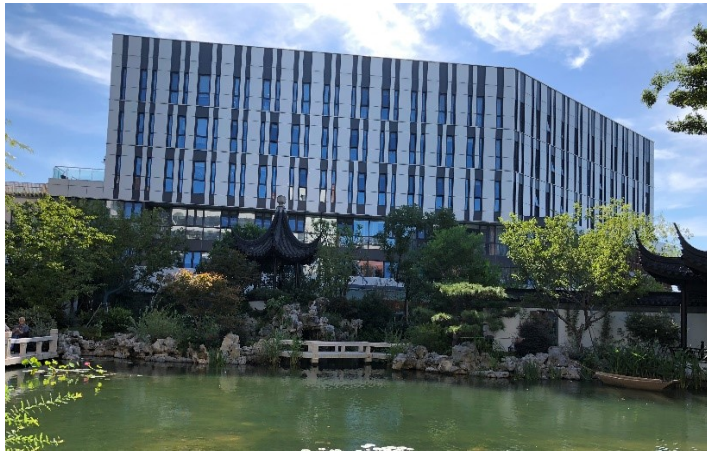
Building Exterior

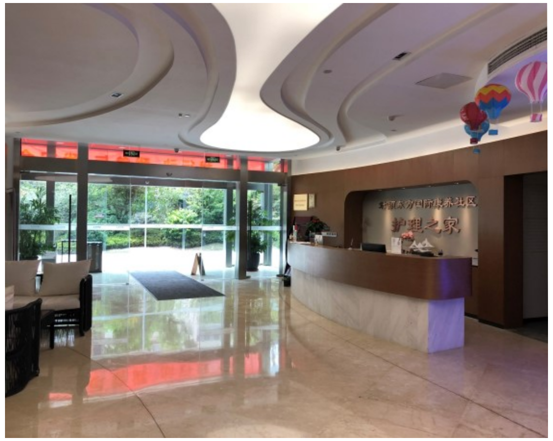
Entry Lobby

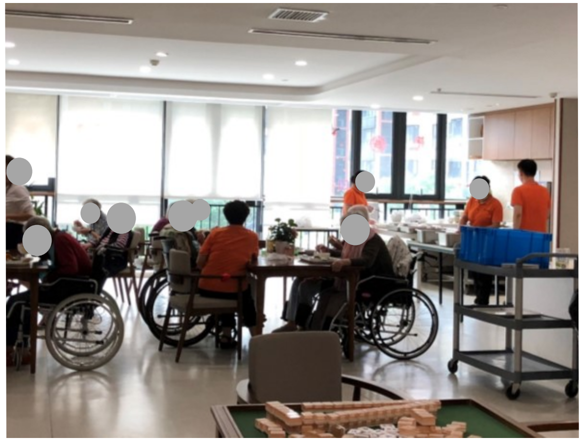
Living Room

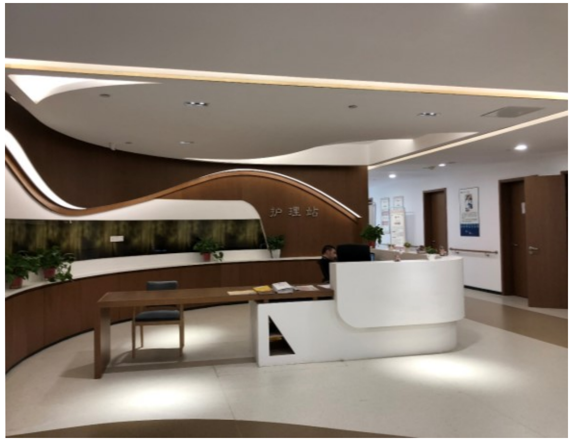
Nursing Station

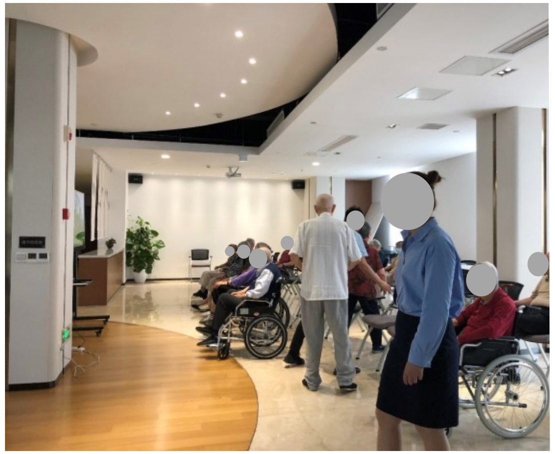
Activity Space

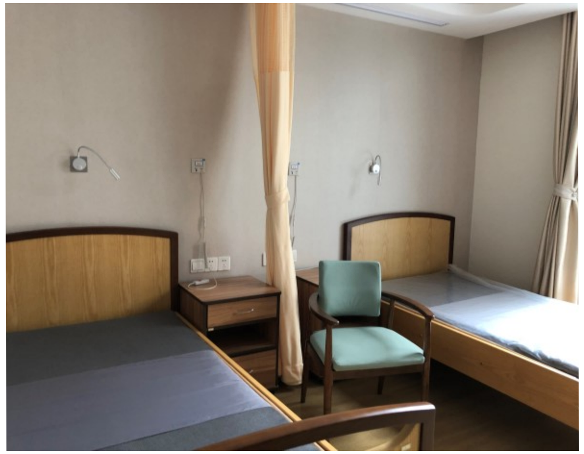
Resident Room

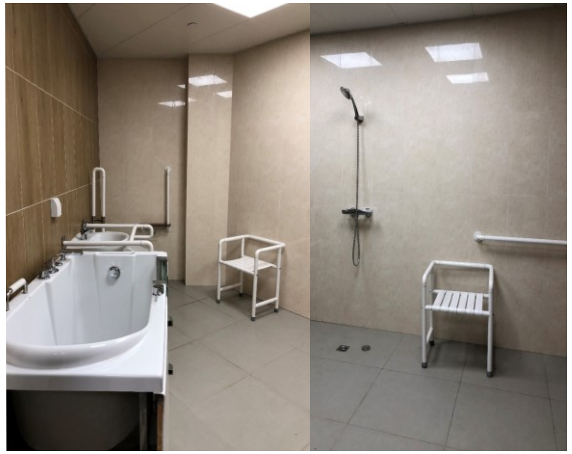
Public Bathroom

Project 34

Location	Opening Year	Building Area	Number of Beds	Building Area per bed	Building Stories	Occupancy Rate	Types of Residents	Number of Staff	Construction Forms
Nanjing	2019	14000㎡	406	34.48 ㎡/beds	Ground 7<br>Ground 5<br>Ground 3	72.7%	Independent;<br>Functional loss;<br>Dementia	200	Renovation

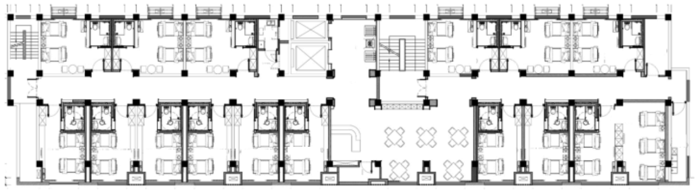

Standard Floor Plan

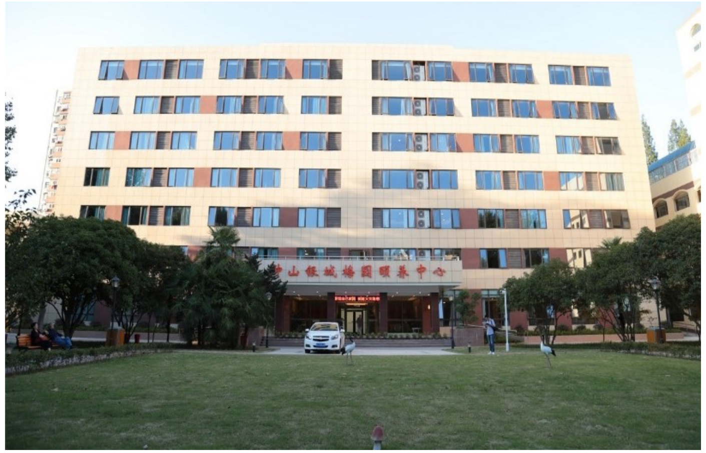

Building Exterior

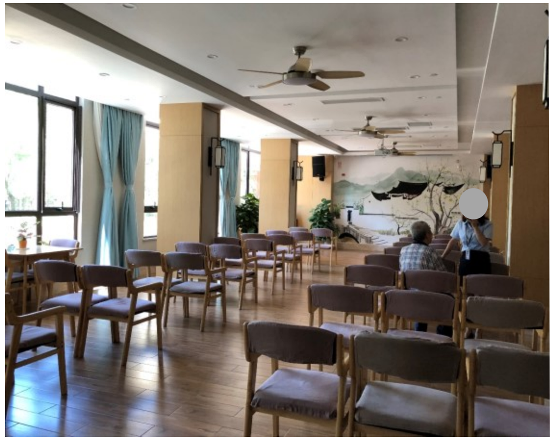

Multi-function Room

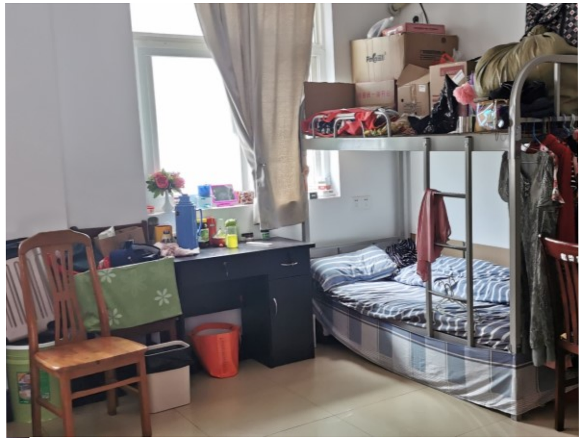

Staff Living Space

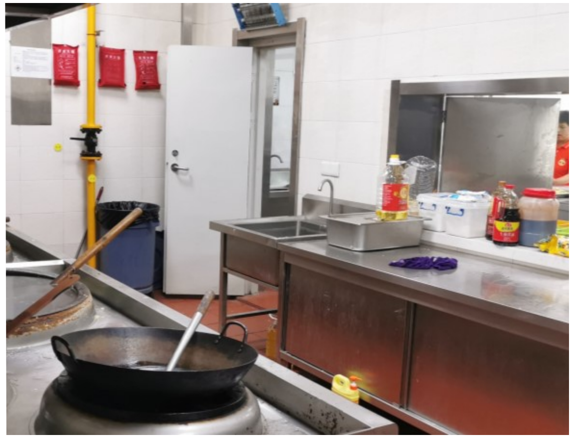

Central Kitchen

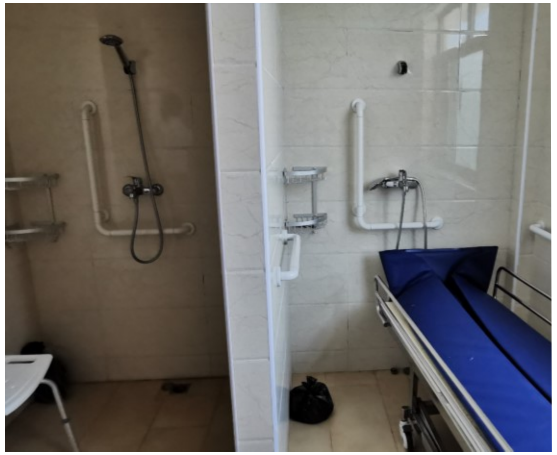

Public Bathroom

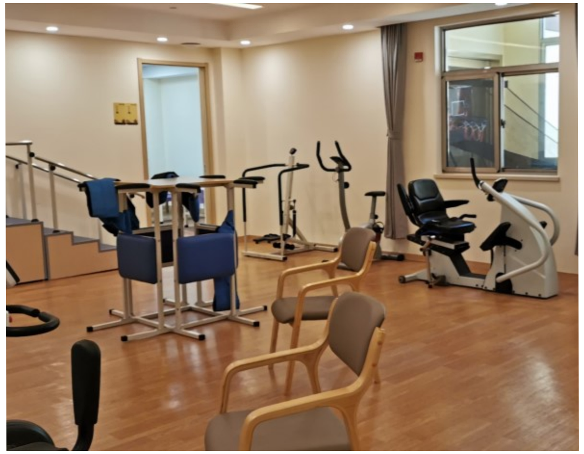

Rehabilitation Space

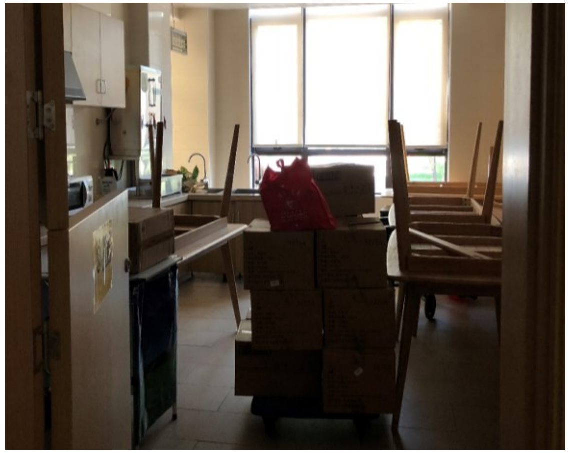

Public Storage Space

Project 35

Location	Opening Year	Building Area	Number of Beds	Building Area per bed	Building Stories	Occupancy Rate	Types of Residents	Number of Staff	Construction Forms
Hangzhou	2017	22000m²	363	60.61 m²/beds	Ground 17 Underground 1	73.0%	Independent; Functional loss; Dementia	190	Newly Built

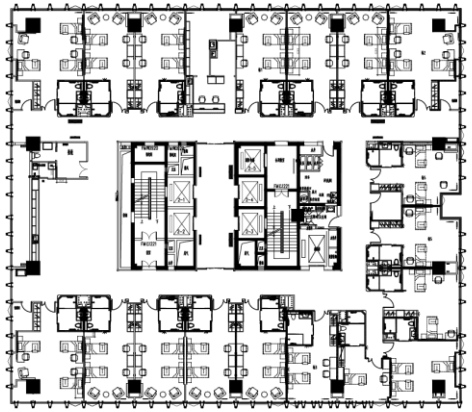

Standard Floor Plan

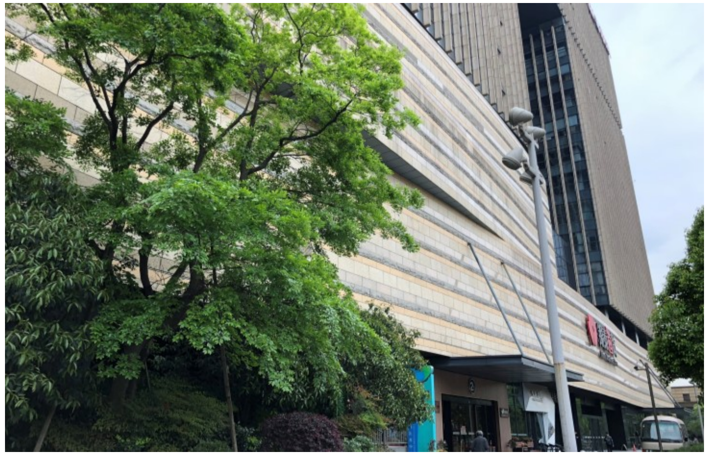

Building Exterior

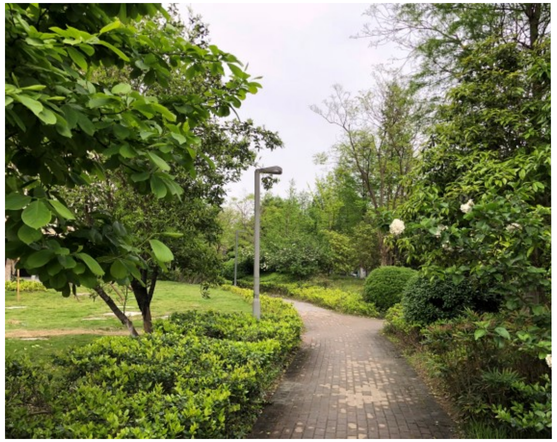

Outdoor Space

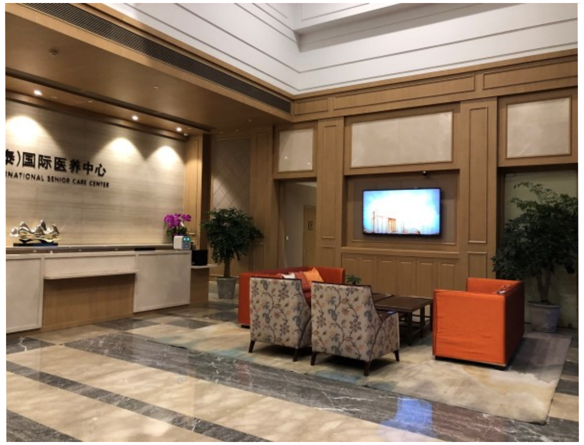

Entry Lobby

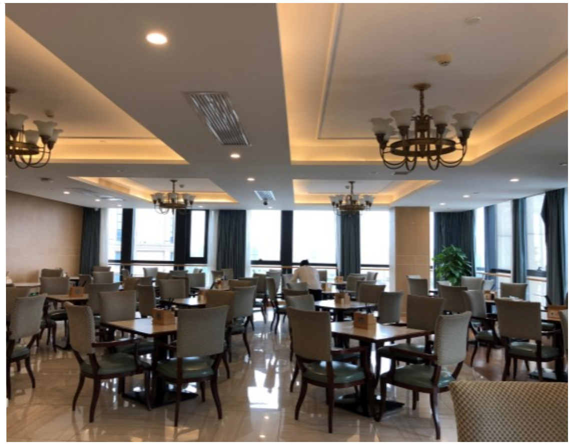

Dining Space

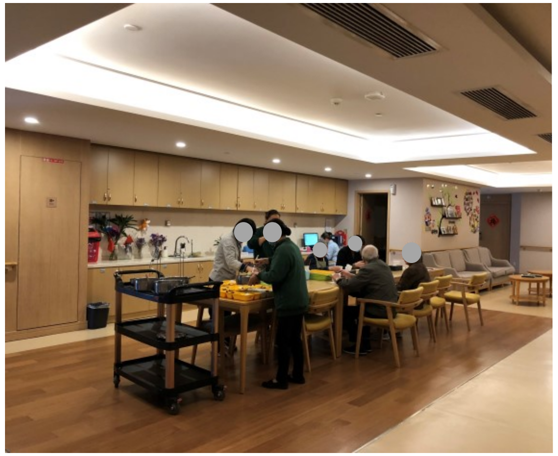

Living Room

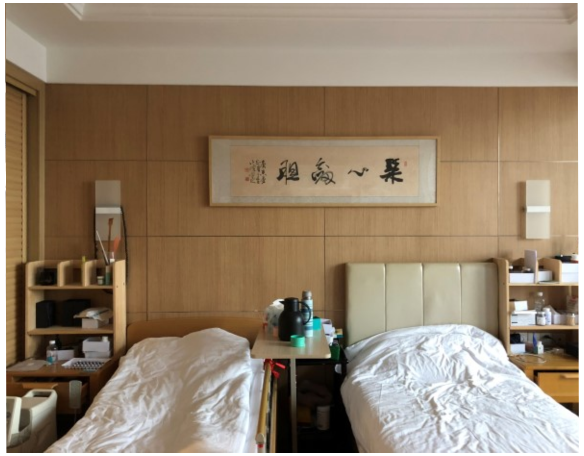

Resident Room

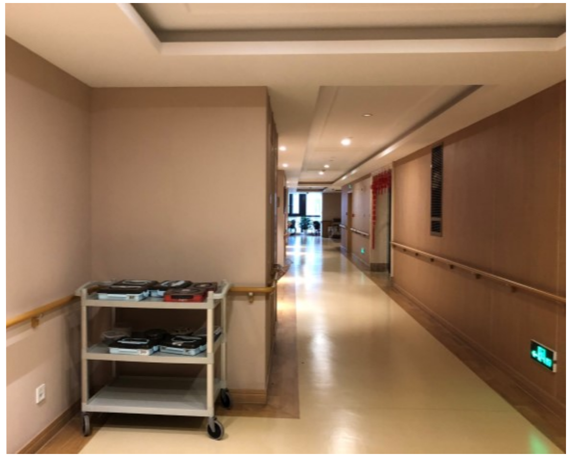

Corridor

Project 36	Location	Opening Year	Building Area	Number of Beds	Building Area per bed	Building Stories	Occupancy Rate	Types of Residents	Number of Staff	Construction Forms
	Hangzhou	2018	3209㎡	140	22.92 ㎡/beds	Ground 4	57.1%	Independent; Functional loss; Dementia	Unknown	Newly Built

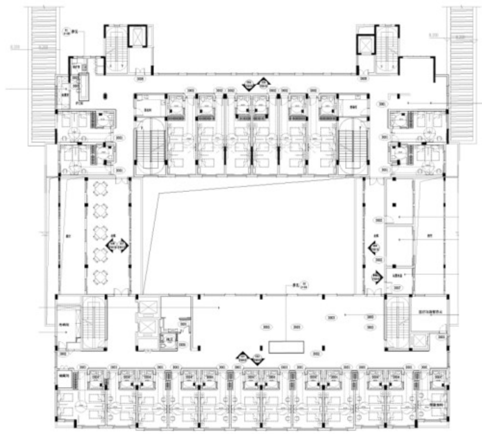

Standard Floor Plan

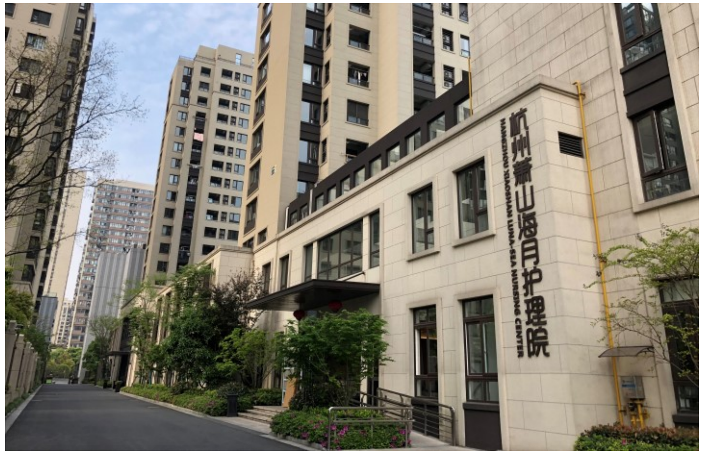

Building Exterior

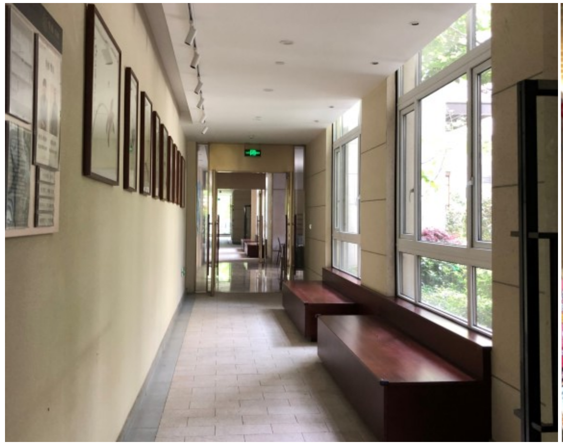

Corridor

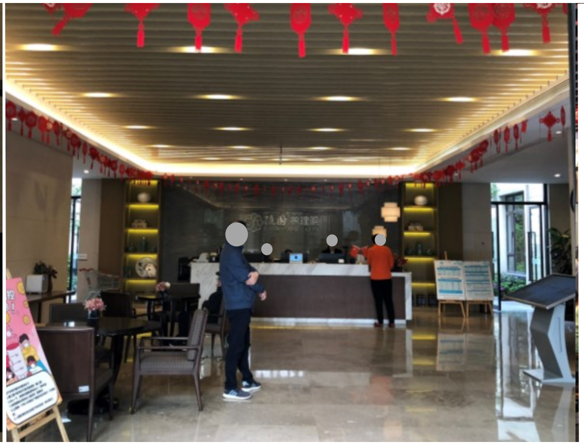

Entry Lobby

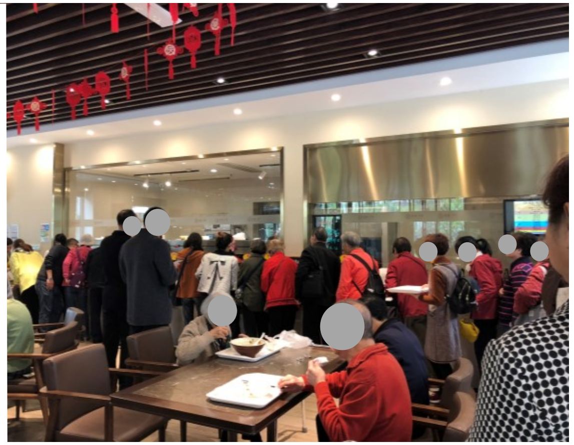

Dining Space

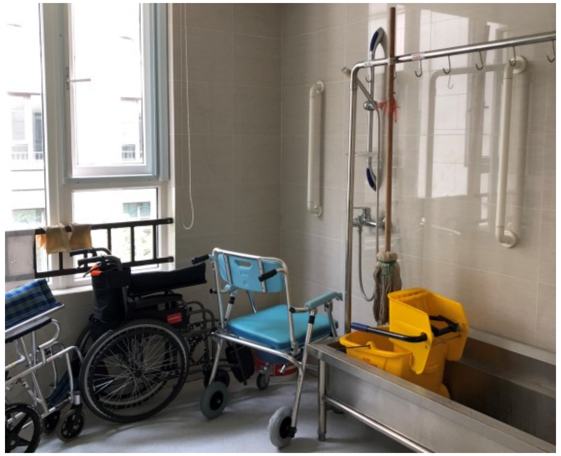

Public Bathroom

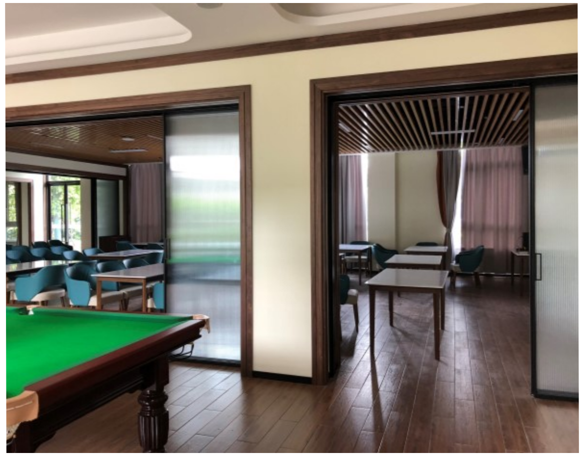

Activity Space

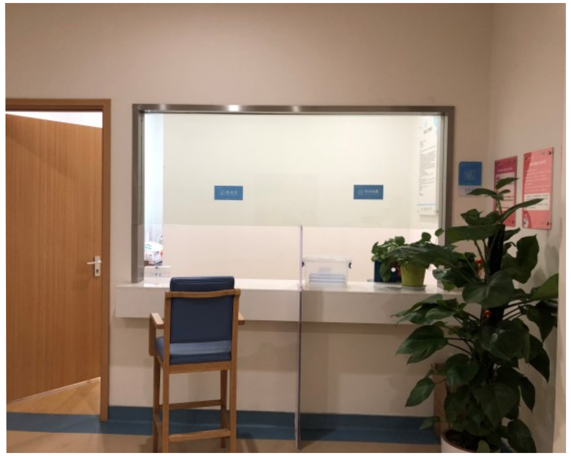

Medical Space

Project 37	Location	Opening Year	Building Area	Number of Beds	Building Area per bed	Building Stories	Occupancy Rate	Types of Residents	Number of Staff	Construction Forms
	Hangzhou	2015	4738㎡	122	38.84 ㎡/beds	Ground 5	99.2%	Independent; Functional loss	Unknown	Newly Built

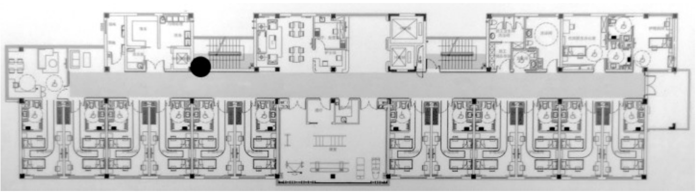

Standard Floor Plan

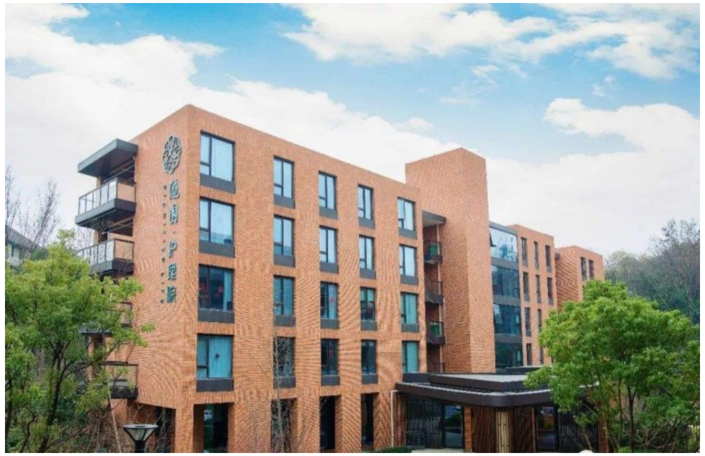

Building Exterior

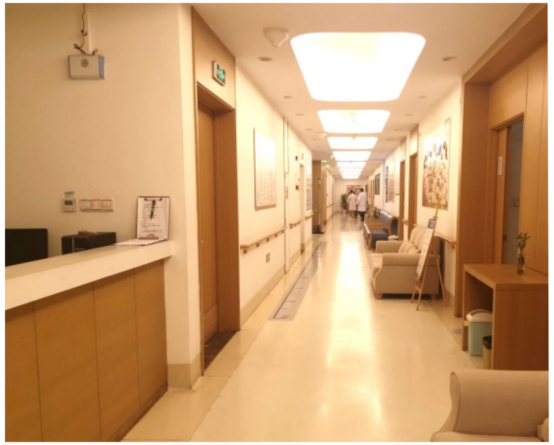

Outdoor Space

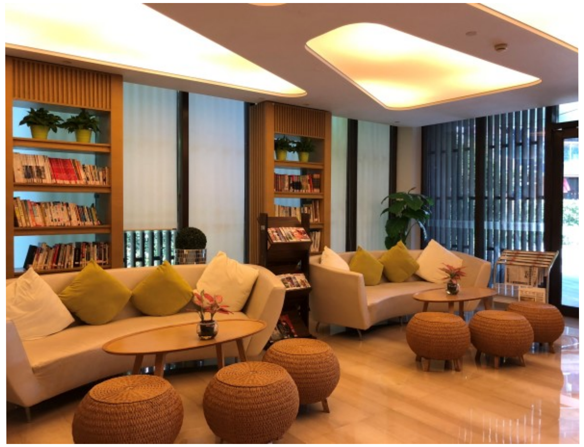

Entry Lobby

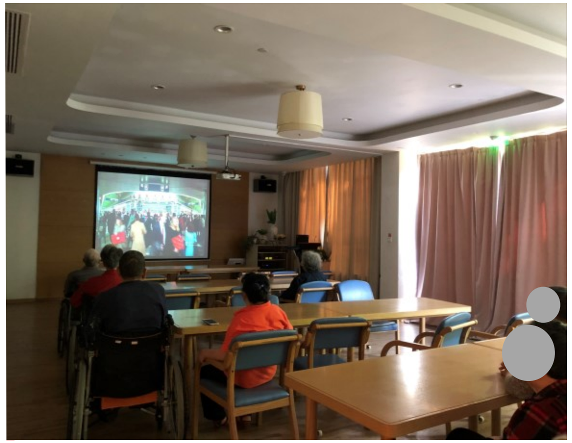

Activity Space

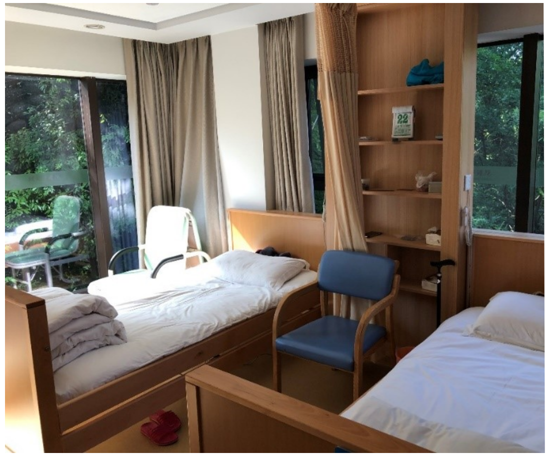

Resident Room

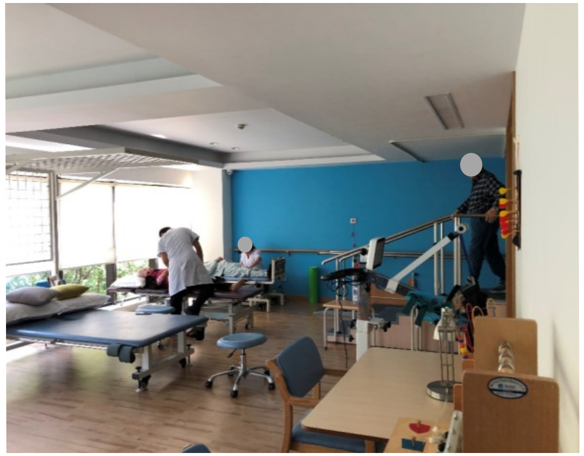

Rehabilitation Space

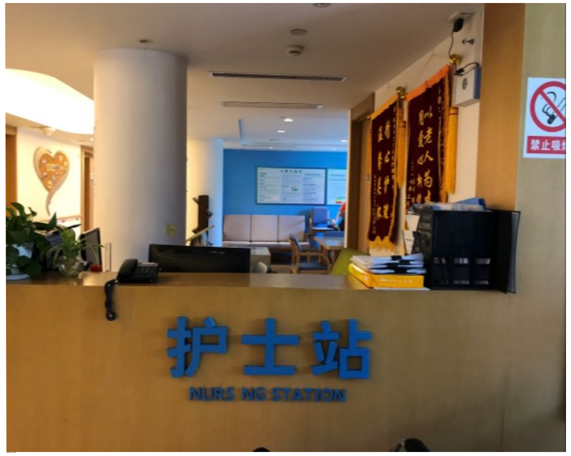

Nursing Station
