# Supplementary material for: Improving life quality for the aged: a comprehensive post-occupancy evaluation of long-term care facilities in China
Source: Front Public Health. 2024 Nov 28;12:1488653. doi: 10.3389/fpubh.2024.1488653 (PMC11636290; doi:10.3389/fpubh.2024.1488653)
Supplement: Supplementary file 2 [file Data_Sheet_2.pdf]

Supplementary Material B

B.1 Survey Questionnaire on Satisfaction with the Physical Environment of Long-term Care Facilities (Version for Residents)

Date: ___________________________ Facility: ____

Researchers: ____________________ Respondents (Room Number): ______________________

Dear respondents,

In order to gain insights into the spatial utilization of this long-term care facility, we are conducting this satisfaction survey. We would be grateful if you could provide us with your true opinion. Your suggestions will contribute to our research on the design of long-term care facilities. This survey will be conducted randomly and anonymously. All responses will be kept confidential. Thank you for your cooperation!

1. Gender

A. Male B. Female

2. Age

A. 60-69 years old B. 70-79 years old C. 80-89 years old D. 90 years old and above

3. Education level (you may also provide information about your previous occupation)

A. Junior high school or below B. High school C. College D. Postgraduate or above

4. Physical health status

A. Independent B. Mild functional loss C. Moderate functional loss

D. Severe functional loss E. Dementia

5. How long have you been residing in this long-term care facility?

A. Less than 6 months B. 6 months to 1 year C. 1 to 2 years

D. 2 to 3 years E. More than 3 years

6. Please rate your satisfaction with the following environmental design elements of this long-term care facility.

The score values represent the following:

1: Very unsatisfied; 2: Fairly unsatisfied; 3: Neutral; 4: Fairly satisfied; 5: Very satisfied;

NA: Not applicable (not aware of the situation)

**Environmental Design Elements**	**Score** 1~5; NA	**Reasons for Scoring**
**1.Traffic Condition Surrounded, TCS**<br>Is the public transportation (subway, bus, etc.) around this long-term care facility convenient? Is parking easily accessible for family visits? Are there any safety hazards within the facility's traffic organization?		
**2.Supporting Facilities Surrounded, SFS**<br>Are there hospitals, shopping malls, supermarkets, parks, libraries, and other facilities around this care facility? Is it convenient to go out from this care facility?		
**3. Outdoor Space, OS**<br>How would you rate the greenery and landscaping of the elderly care facility? Are the outdoor spaces safe, diverse, and accessible? What activities would you like to do outdoors? Do you need public restrooms outdoors? Do you think specialized walking paths are needed?		
**4. Building Scale, BS**<br>Is the overall number of beds in the facility appropriate? Do you feel crowded? Is the number of residents in each care unit suitable? Are residents familiar with each other?		
**5. Functional Organization, FO**<br>What do you think of the floor plan shapes (e.g., linear, L-shaped)? Do you think that care units should be independent of each other? Are you willing to live on the same floor with dementia patients? Do you have the need to communicate with the residents on other floors? Is your daily life disturbed by visitors? Do you think spaces such as cleaning, laundry, and similar service areas interfere with your life?		
**6. Resident Room, RR**<br>Do you like your room? If you could choose freely, which type of room would you prefer? Are you willing to live in a room facing north? Can your privacy be guaranteed in multi-bed rooms? Do you think the size of room and furniture equipment can meet your daily needs? Are there any furniture you would like to add (such as refrigerators, washing machines, induction cooker, storage cabinets, etc.)? Are you able to decorate the room according to your own wishes? Is the bathroom convenient to use? Would you mind sharing a bathroom or shower? Is there a doorplate on your room so you can easily find it?		
**7. Living Room, LR**<br>Does the location and layout of the living room in this long-term care facility meet your daily needs? Is it convenient to seek assistance from the nursing station?		
**8. Public Toilet, PT**<br>Are the location and number of public toilets adequate? Do you think the public toilets are safe and convenient to use? Could the toilets meet the requirements when assistance from caregivers is needed?		
**9. Public Bathroom, PB**<br>Do you need assistance from caregivers for bathing? Have you ever used the public bathroom? Where do you prefer to take a bath? Is the public bathroom safe and convenient to use? Is there a need for a specialized barber room?		
**10. Entry Lobby, EL**<br>Are there any reception services for your family when they come to visit? Do you enjoy engaging in activities in the entry lobby? Are there any deficiencies of the entry lobby?		

11. Dining Space, DS

Where do you usually have meals? Is the dining space too crowded? Are the dining tables, chairs, and other furniture comfortable? Which form of meal service do you prefer (buffet, set menu, or à la carte)? Where do your family members eat when they come to visit? Would you prefer a separate dining area? Do you think hand-washing sinks in the dining space are necessary?

12. Activity Space, AS

Are the location, size, and type of activity spaces appropriate? What activities do you most enjoy participating in? How often? Do you prefer individual or group activities? Is there any activity space that you would like to increase? Would you like to increase the spaces shared with neighboring residents? Is there a designated area for family visits? Is there a need for a multifunctional room?

13. Entrance, Corridor, Staircase & Elevator, ECSE

Do the entrances, corridors, staircases, and elevators of the building comply with barrier-free requirements? Are there any areas that are difficult to access while in a wheelchair or using a walking aid? Have there been any incidents of falling or tripping? Is there a need for seating near the elevators?

14. Medical & Rehabilitation Space, MRS

Does the location and size of the medical rehabilitation spaces meet the fundamental needs for care? Are there any additional spaces you would like to see added? Is there a need for a separate area for palliative care? Is there a need for a restroom nearby?

15. Signage System, SS

Are access guide signs and other signage helpful to you? Is the location of the signage reasonable? Is the font size, and format of the signage visible and easy to understand?

16. Lighting, Ventilation, Temperature & Sound, LVTS

Is the temperature and humidity suitable in your living spaces? How would you evaluate the natural lighting, artificial illumination, and ventilation conditions in each space? Is the living environment comfortable? Are there any noises or reverberations?

17. Home-Like Qualities, HLQ

Is the overall physical environment cozy, home-like, and conducive to a sense of happiness? Do you like the interior design style? Are all spaces clean and tidy?

7. Based on the evaluations of items mentioned above, provide an Overall Satisfaction rating (1~5): ________________

8. Which necessary spaces do you think are lacking in this long-term care facility? Are there any other deficiencies in the existing physical environment?

____

This concludes the questionnaire. Thank you for your participation!

Supplementary Records:

____

____

B.2 Survey Questionnaire on Satisfaction with the Physical Environment of Long-term Care Facilities (Version for Staff)

Date: ______________________________ Facility: ___________________________________

Researchers: ________________________Respondents (Occupation): __________________

Dear respondents,

In order to gain insights into the spatial utilization of this long-term care facility, we are conducting this satisfaction survey. We would be grateful if you could provide us with your true opinion. Your suggestions will contribute to our research on the design of long-term care facilities. This survey will be conducted randomly and anonymously. All responses will be kept confidential. Thank you for your cooperation!

1. Gender

A. Male B. Female

2. Age

A. 18-30 years old B. 31-40 years old C. 41-50 years old

D. 51-60 years old E. 60 years old and above

3. Education level

A. Junior high school or below B. High school C. College D. Postgraduate or above

4. How many years have you been engaged in elderly care services?

A. Within half a year B. Half a year to 1 year C. 1 to 3 years

D. 3 to 5 years E. More than 5 years

5. Please rate your satisfaction with the following environmental design elements of this long-term care facility.

The score values represent the following:

1: Very unsatisfied; 2: Fairly unsatisfied; 3: Neutral; 4: Fairly satisfied; 5: Very satisfied;

NA: Not applicable (not aware of the situation)

Environmental Design Elements	Score 1~5; NA	Reasons for Scoring
**1.Traffic Condition Surrounded, TCS**<br>Is the public transportation (subway, bus, etc.) around this long-term care facility convenient? How do you commute? Where are non-motorized vehicles parked?		
**2.Supporting Facilities Surrounded, SFS**<br>Are there hospitals, shopping malls, supermarkets, parks, libraries, and other facilities around this care facility? Is it convenient to go out from this care facility?		
**3. Outdoor Space, OS**<br>How would you rate the greenery and landscaping of the elderly care facility? Are the outdoor spaces safe, diverse, and accessible?		
**4. Building Scale, BS**<br>Is the overall number of beds in the facility appropriate? Do you feel crowded? How many beds are there in each care unit? How many caregivers are there? Is the number sufficient to adequately care for all the residents?		
**5. Functional Organization, FO**<br>Are the living spaces for the residents, medical spaces, and cleaning spaces independent of each other and free from interference? Is it possible to implement closed management during epidemic periods? Is it necessary to set up a specialized living area for the residents with dementia?		
**6. Resident Room, RR**<br>Is the area of the resident room sufficient? Is it convenient for nursing services?		
**7. Living Room, LR**<br>Are the locations of the living room and the nursing station reasonable? Are they convenient when caring for residents? Is the size of the living room and the furniture configuration convenient for organizing daily activities? What types of activities are typically conducted there? Is the living room also used as a dining area? How often? Is there a food preparation room or table? Is its positioning in relation to the nursing station appropriate? Is the size and configuration of the area convenient to use?		
**8. Public Toilet, PT**<br>Are the location and number of public toilets adequate? Do you think the public toilets are convenient to use? Are they convenient when caring for residents? Is there a public toilet for staff? Is it necessary?		
**9. Public Bathroom, PB**<br>Are the number and location of public bathrooms reasonable? Is the interior space and facility configuration convenient for bathing assistance? What is the frequency of use? What is the bathing assistance process?		
**10. Entry Lobby, EL**<br>Can the entry lobby fulfill functions such as reception, rest, and promotion?		
**11. Dining Space, DS**<br>Are there enough dining seats? Are the seats conveniently located for meal delivery? Is there a food preparation room or table? Is the dining space too crowded?		
**12. Activity Space, AS**<br>Are the location, size, and type of activity spaces appropriate? Are they convenient for activity organization and supervision?		
**13. Entrance, Corridor, Staircase & Elevator, ECSE**<br>Do the entrances, corridors, staircases, and elevators of the building comply with barrier-free requirements? Have there been any incidents of residents falling? If so, where do these incidents most commonly occur?		
**14. Medical & Rehabilitation Space, MRS**<br>Does the location and size of the medical rehabilitation spaces meet the fundamental needs for care? Does it provide services such as medication dispensing, assessment, medical records management, medical waste disposal, and palliative care? Is there a specialized rehabilitation space?		

15. Central Kitchen, CK
Is the central kitchen's location, area, and configuration convenient for use? What are the processes for food delivery and meal preparation?

16. Laundry Space, LS
What is the laundry mode? Is the location, size, and configuration of the laundry space convenient for washing, drying, and storing clothes?

17. Cleaning Space, CS
Is the location, size, and configuration of the cleaning space convenient for use? Are the cleaning and waste disposal rooms separate from each other?

18. Public Storage Space, PSS
Is the location, size, and number of the storage rooms convenient for use? Where is the waste temporarily stored, and is its location reasonable?

19. Staff Working Space, SWS
Is the size and type of the staff working space reasonable? Is there a need for independent offices for caregivers? Are there requirements for meeting and training rooms? Is a social worker's office required?

20. Staff Living Space, SLS
Are there appropriate spaces for caregivers to change clothes, dine, and rest?

21. Signage System, SS
Is the location of the signage reasonable? Is the font size, and format of the signage visible and easy to understand?

22. Fire Protection Facilities, FPF
Are the fire protection facilities in this long-term care facilities compliant with regulations? Do they interfere with daily activities?

23. Lighting, Ventilation, Temperature & Sound, LVTS
Is the temperature and humidity suitable in your working spaces? How would you evaluate the natural lighting, artificial illumination, and ventilation conditions in each space? Is the living environment comfortable? Are there any noises or reverberations?

24. Home-Like Qualities, HLQ
Is the overall physical environment cozy, home-like, and conducive to a sense of happiness? Do you like the interior design style? Are all spaces clean and tidy?

6. Based on the evaluations of items mentioned above, provide an Overall Satisfaction rating (1~5): _________________

7. Which necessary spaces do you think are lacking in this long-term care facility? Are there any other deficiencies in the existing physical environment?

____

This concludes the questionnaire. Thank you for your participation!

Supplementary Records:

____

____

B.3 Development methods for the environmental assessment tool in this study

The environmental assessment tool used in this study contains 160 indicators, which were derived from existing literature related to environment design of LTCFs. The primary references are divided into international and local literature, encompassing three categories: tools/standards, empirical/review articles, and expertise/guidelines as detailed in Table A1. And Figure A1 illustrates the protocol used for sourcing empirical/review articles.

Table A1. The categories of references.

International References		Local References	
**Tools/Standards (main references)**			
**Environment Assessment Tools/Scales (OT) (based on a non-rigorous umbrella review)**	15 tools referenced in this study: PAF, SCEAM, TESS-NH/RC, EQUAL, SCEAM, E-B Model, PEAP, TESS-NH, SCUEQS, PEAP (Japanese version), EAT, DDAT, EAT-HC, SAGE's tool, AIA's tool	**Design/ Assessment Standards (IS)**	*Guidelines of Classification and Accreditation for Senior Care Organization*<br>*Standard for Design of Care Facilities for the Older Adults*<br>*Standard for Urban Residential Area Planning and Design*<br>*Codes for Fire Protection Design of Buildings*<br>*Codes for Accessibility Design*
**Empirical/Review Articles (based on a systematic review)**			
**English Literatures (OL)**	Database: Web of Science, Medline, PsycINFO<br>Topic: Review of design for the physical environment of elder care facilities<br>Result: 14 review studies	**Chinese Literatures (IL)**	Database: CNKI<br>Topic: Physical environmental design of elderly care facilities<br>Result: 22 empirical/review studies
**Expertise/Guidelines**			
**International Books/ Guidelines (OE)**	*Design for Assisted Living;*<br>*Housing Design for an Increasingly Older Population;*<br>*Design Guide for Long Term Care Homes (2018 edition)*	**Chinese Books/ Guidelines (IE)**	*Design and Interpretation of Elderly Care Facility*<br>*Living Environment Design for the Older Adults*<br>*Care Facilities and Residential Architectures for the Older Adults*

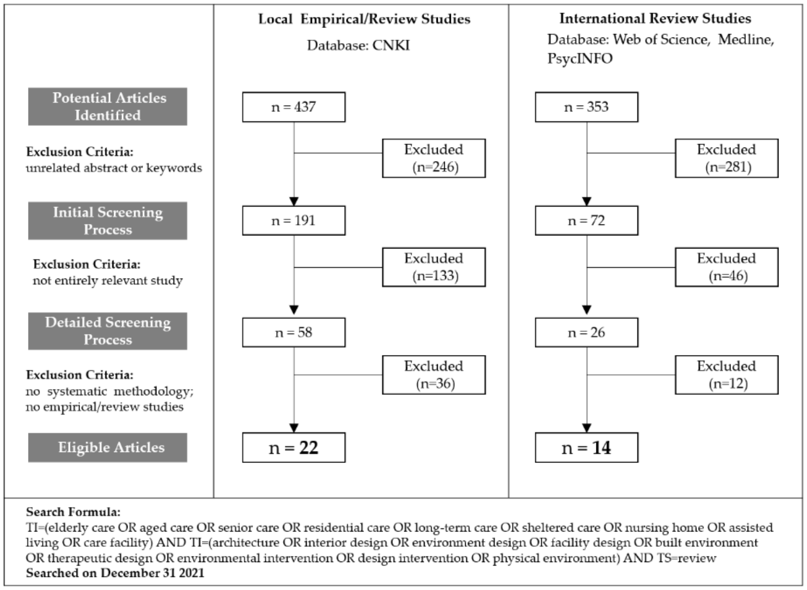

Figure A1. The protocol used for sourcing empirical/review articles.

Through the identification and organization of the key points of environmental design for user needs from the above references, the evaluation indicators were developed using the following three methods.

International References (M1): 22(13.8%) items were translated and adapted from existing environment assessment tools.

Local References (M2): 41(25.6%) items were derived from Chinese policy documents, design standards and codes, especially the Guidelines of Classification and Accreditation for Long-term Care Organization, which was co-authored by a researcher (Y.C.) involved in this study.

Integration and Composition (M3): 97(60.6%) items were derived through a comprehensive analysis of different categories of references. There were instances where certain content requiring assessment may have multiple sources that can be referenced or no direct reference items available. In either of the situations, an integration and composition method were utilized.

The content, references and formulating methods of 160 Assessment Indicators are shown in the Supplementary Material B.4.

frontiers

B.4 Information about the Environmental Assessment Tool for LTCFs

Environmental Design Elements (24)	No.	Assessment Indicators (160)	Environmental Design Principles (14)	References of Indicators	Methods of Formulating Indicators
Traffic Condition Surrounded, TCS (6)	1	At least one public transportation stop (such as bus stop, rail stop, etc.) located within 300 meters of the main entrance of the care facility.	Accessibility	IS; OT	M3
	2	The traffic organization within the care facility is safe and convenient, meeting the following conditions:<br>(1) The traffic flow is efficient and meets the demands for evacuation and transportation.<br>(2) Roads are segregated from pedestrians and vehicles.	Safety and Health, Accessibility	IS; IL; IE; OT	M3
	3	The passage through the vehicle traffic area and the outdoor activity space is barrier-free, allowing wheelchairs or walking aids to access and turn around.	Physical Frailty Support, Accessibility	IS; IE; OT; OL	M2
	4	The care facility has barrier-free motorized parking spaces, meeting the following conditions:<br>(1) The parking space is located near the main entrance of the building.<br>(2) Width of passageway along one side of the parking space ⩾ 1.2 m, providing direct access to the sidewalk and main entrance.<br>(3) The parking space is clearly signed with parking lines, wheelchair access lines, and barrier-free signs painted on the ground.	Physical Frailty Support, Functional Support	IS; IE; OL	M2
	5	The care facility is equipped with motorized parking space within or near the entrances, meeting the following conditions:<br>(1) The parking space is easily accessible to vehicles, especially ambulances that need to park directly at the main entrances.<br>(2) The passage from the parking space to the main entrance is barrier-free.<br>(3) The number or size of parking spots meets the daily parking needs.	Functional Support, Accessibility	IS; IE; OT; OE	M3
	6	The care facility offers shaded, non-motorized parking.	Staff Facilities, Accessibility	IS; IE	M3
Supporting Facilities Surrounded, SFS (4)	7	Medical care facilities, such as community health service centers, are located within 1 kilometer or a 15-minute walking distance from the care facility and offer daily healthcare services for common diseases.	Functional Support, Safety and Health	IS; OE	M2
	8	Medical care facilities and emergency care facilities for acute and critical illnesses are located within 5-kilometer or a 15-minute drive of the care facility.	Functional Support, Safety and Health	IS; IE	M2

	9	At least one commercial service facility, such as a shopping mall, food market, supermarket, convenience store, dining facility, bank outlet, or telecommunication outlet, is located within 1 kilometer or a 15-minute walking distance from the care facility.	Social-Recreational Support	IS; IL; OT; OL; OE	M2
	10	At least one public green space, park, or cultural activity facility, such as a senior activity center or university for the elderly, is located within 1kilometer or a 15-minute walking distance from the care facility. These facilities provide support for recreational and sports activities for residents.	Social-Recreational Support, Outdoor Freedom	IS; IL	M2
Outdoor Space, OS (10)	11	The outdoor planting of grass, shrubs, trees, and other plants is well-adapted to the local climate, resulting in optimal growth, lush vegetation. The greening covers a certain area.	Outdoor Freedom	IS; IL; IE; OL; OE	M3
	12	There are no plants that produce flotsam, have thorns or easily exposed roots in pedestrian areas, obstruct pedestrian traffic with sprawling branches, or contain poisonous elements.	Safety and Health	IS; IE; OL	M3
	13	The care facility is equipped with outdoor activity spaces that are easily visible and accessible from the living rooms and dining spaces, attracting residents to go out.	Outdoor Freedom, Accessibility	IS; IL; IE; OT; OL; OE	M1
	14	The outdoor activity space is safe for use and meets the following conditions:<br>(1) Not intersect with vehicular traffic area.<br>(2) The floor is even, non-slip, with no pooling water.<br>(3) The floor material is anti-glare.<br>(4) Lighting is provided along the primary walking paths, surrounding the main activity area, and at the stairs.	Safety and Health	IS; IE; OT; OL; OE	M3
	15	No thresholds in the floor of outdoor activity spaces, making it convenient for residents who use wheelchairs or walking aids.	Physical Frailty Support	IS; IL; IE; OT; OL; OE	M3
	16	The outdoor activity space for residents with dementia is carefully maintained and securely enclosed to prevent any wandering off.	Cognitive Support	IL; IE; OT; OL; OE	M1
	17	The care facility is equipped with at least three of the following outdoor spaces:<br>(1) A sunny and sheltered space with access to sunlight for residents to sunbathe.<br>(2) A shaded rest area with seating, such as a porch or gazebo.<br>(3) At least one quiet and private resting space for residents to be alone or have private conversations.<br>(4) A hard-paved area suitable for group exercise.	Outdoor Freedom, Social-Recreational Support	IS; IL; IE; OT; OL; OE	M3
	18	Outdoor walking paths meet at least two of the following conditions:<br>(1) Width of at least one walking path ⩾1.2m, allowing for wheelchair access and staggered passing for residents. Additionally, some sections of the walking path are widened to a width of ⩾1.8m to accommodate staggered wheelchair travel.<br>(2) Provide a selection of walking routes with varying lengths and different scenic views for residents.<br>(3) The walking path is equipped with seating areas, allowing residents to take rest.	Outdoor Freedom, Choice and Control	IS; IE; OT; OL; OE	M3
	19	The outdoor area is equipped with at least two of the following types of landscaping facilities:<br>(1) Landscape vignettes, such as flower beds and sculptures.	Outdoor Freedom,	IS; IE; OT; OL; OE	M3

		(2) Water features, including pools and fountains. When installing ornamental water feature pools, it is essential to incorporate safety tips and protection measures.<br>(3) Horticultural operation sites or planting flower box facilities, such as raised planting beds.<br>(4) Roof gardens or greenhouse gardens.<br>(5) Therapeutic landscapes designed for residents with dementia, providing elements that stimulate the senses and allow for group activities.	Choice and Control		
	20	The outdoor activity space is equipped with public toilet for residents nearby.	Physical Frailty Support	IS; IE; OT	M2
Building Scale, BS (4)	21	Number of beds in care facilities ≤ 500.	Functional Support, Home likeness	IL; IE; OL	M3
	22	Average floor area per bed ≥ 35 ㎡.	Functional Support	IS; IL; IE	M3
	23	Number of beds per care unit ≤ 60.	Home likeness	IS; IL; IE; OE; OL	M3
	24	Number of beds per dementia care unit ≤ 20.	Cognitive Support, Home likeness	IS; IL; IE; OT; OL; OE	M3
Functional Organization, FO (4)	25	The living spaces for residents and the working spaces for staff are separated, with each functional area having its own separate entrance.	Functional Support, Comfort	IS; IE	M3
	26	The living spaces for residents are organized into self-contained care units, equipped with the capability to isolate and prevent cross-infections.	Functional Support, Safety and Health	IS; IL; IE; OL	M3
	27	The care facility has special care units for residents with dementia.	Cognitive Support	IS; IE; OT; OL	M1
	28	The flow lines for residents living and staff working are independent of each other and do not intersect.	Safety and Health, Comfort	IS; IL; IE; OT	M3
Resident Room, RR (18)	29	The care facility has different types of resident room options for the elderly to choose from based on their needs and preferences, including single room, double room, suites, etc.	Choice and Control, Functional Support	IE; OT; OL	M1
	30	Single rooms account for over 50% of all rooms.	Privacy, Home likeness	IS; IL; OL	M3
	31	The rooms for residents with dementia are single rooms.	Cognitive Support	IS; IL; IE; OT; OL	M1
	32	Number of beds in the multiple rooms for residents with moderate impairment ≤ 4. Number of beds in the multiple rooms for residents with severe impairment ≤ 6.	Home likeness	IS; IE	M2

33	In double rooms and multiple rooms, each bed is equipped with curtains or is partitioned by furniture for privacy.	Privacy	IS; IL; IE; OT; OL	M2
34	80% or more of all rooms have the attached bathroom.	Privacy, Physical Frailty Support	IS; OT; OL	M3
35	The rooms are barrier-free, meeting the following conditions:<br>(1) The entrance has no threshold, or has a heigh difference ⩽15mm, with a sloping transition.<br>(2) Width of doorway ⩾0.8m. When nursing beds need to access, the width of doorway ⩾1.1m.<br>(3) The opening door does not obstruct the public corridor access.<br>(4) Width of the main corridor within the room ⩾1.05m, allowing wheelchairs or walking aids to access, turn around and parking.	Physical Frailty Support	IS; IL; IE; OT; OL; OE	M3
36	The rooms have good safety, meeting the following conditions:<br>(1) Equipped with the emergency call device or wearable emergency call device for residents.<br>(2) Windows or balconies are equipped with safety protection features.	Safety and Health	IS; OT	M3
37	Usable area of the single room⩾ 10.00 ㎡, of the double room ⩾ 16.00 ㎡ per bed.	Functional Support	IS; IE; OE	M3
38	The rooms are equipped with basic furniture and amenities to meet daily living needs, meeting the following conditions:<br>(1) Equipped with essential furniture such as the bed, bedside table/desk, chairs/stools.<br>(2) Equipped with storage spaces like wardrobes/storage cabinets.<br>(3) Equipped with the refrigerator, television, and have the necessary conditions for installing a washing machine.<br>(4) Equipped with electric sockets that are convenient for residents to use.	Functional Support, Physical Frailty Support	IS; IE; OT; OL; OE	M3
39	Furniture and equipment in double rooms and multiple rooms are equitably set, ensuring residents have no interference with each other while using.	Functional Support	IS; IE	M2
40	The bedside has enough space for staff to provide care and services for residents conveniently.	Service Support	IS; IE	M2
41	The room entrance is featured with room numbers or other signs, or has facilities and spaces with personalized decorations, helping residents finding their way home.	Accessibility, Home likeness	IS; IE; OT; OL; OE	M1
42	The rooms have spaces and facilities for personal photographs, books, and furniture, offering residents the flexibility to arrange their rooms as they wish.	Choice and Control, Home likeness	IL; IE; OT; OL; OE	M1
43	The bathroom within the room is barrier-free, meeting the following conditions:<br>(1) The entrance has no threshold or has a height difference ⩽15mm, with a sloping transition.<br>(2) Width of doorway ⩾0.8m.<br>(3) The door can be opened outwards or is designed as a sliding door.<br>(4) The internal space allows wheelchairs or walking aids to access and turn around.	Physical Frailty Support	IS; IL; IE; OT; OL; OE	M3
44	The bathroom within the room is safe for washing, toileting and having a bath, meeting the following conditions:	Safety and Health,	IS; IL; IE; OT; OL; OE	M3

		(1) The floor is even, slip-resistant, and well-drained, with no pooling water.<br>(2) There are at least 2 lighting facilities providing sufficient and even illumination, without obvious shadow areas.<br>(3) Equipped with toilet facilities suitable for residents.<br>(4) Equipped with accessible washbasin, that are convenient for wheelchairs or walking aids.<br>(5) Equipped with shower facilities suitable for residents and has space for shower chairs.<br>(6) Necessary handrails are installed in the toilet and shower areas to assist residents.<br>(7) Equipped with emergency call devices that are easy for residents to use.<br>(8) The path from room to bathroom is equipped with nightlights or smart sensor lights.	Physical Frailty Support		
	45	In the bathroom within the room, there is sufficient supporting space near the washbasin, toilet, and the shower, allowing staff to help residents wash, toilet and have a bath.	Service Support	IS; IE; OE	M2
	46	There are doors, curtains, or other view barriers at the entrance of the bathroom within the room.	Privacy	IS; IE; OT; OL	M3
Living Room, LR (6)	47	The care unit has a living room, which is centrally located and easily accessible for residents in all rooms.	Functional Support, Accessibility	IE; OT; OL	M1
	48	The living room is equipped with various types of tables, chairs, the television, and other furniture and equipment required for daily activities, meeting the needs of residents for communication, exercise, handicrafts, card games, watching television, and other activities.	Functional Support, Social-Recreational Support	IS; IL; IE; OT; OL; OE	M3
	49	The care unit has a dining room near or combined with the living room, whose location is convenient for meal service.	Functional Support, Accessibility	IS; IE; OT; OL; OE	M1
	50	The dining room is equipped with a meal preparation space and necessary facilities (including sinks, countertops, electric sockets, etc.) for staff to prepare meals. The location is reasonable, allowing staff to supervise residents during meal preparation.	Service Support, Work Support	IS; IL; IE; OL	M3
	51	The care unit has a nursing station, which is centrally located, easily identified, and near the living room, making it convenient for staff to monitor activities in living room and corridors.	Work Support, Accessibility	IS; IL; IE; OT; OL	M3
	52	The nursing station is in a semi-open shape and is equipped with an operation desk, essential appliances and facilities to meet basic operational needs.	Work Support	IL; IE	M3
Public Toilet, PT (5)	53	The care facility is equipped with public toilets, meeting the following conditions:<br>(1) There are public toilets for family members and staff near the entry lobby.<br>(2) There are public toilets for residents near the living rooms and dining spaces.<br>(3) The care unit has a public toilet for residents.	Functional Support, Accessibility	IS; IL; IE; OT; OL; OE	M3
	54	The public toilet is barrier-free, meeting the following conditions:<br>(1) The entrance has no threshold or has a height difference ⩽15mm, with a sloping transition.<br>(2) Width of doorway ⩾0.8m.<br>(3) The internal space allows wheelchairs or walking aids to access and turn around.	Physical Frailty Support	IS; IL; IE; OT; OL; OE	M3

	55	The public toilet is safe for toileting, meeting the following conditions:<br>(1) The floor is even, slip-resistant, easy to clean, and well-drained, with no pooling water.<br>(2) Equipped with toilet facilities suitable for residents.<br>(3) Equipped with accessible washbasin, that are convenient for wheelchairs or walking aids.<br>(4) Necessary handrails are installed in the toilet area to assist residents.<br>(5) There is an emergency call device that is easy for residents to use.	Safety and Health, Physical Frailty Support	IS; IL; IE; OT; OL; OE	M3
	56	There is sufficient supporting space near the washbasin and toilet, allowing staff to assist residents.	Service Support	IS; IE; OT; OE	M3
	57	The public toilet ensures the users' privacy, meeting the following conditions:<br>(1) Separate toilets for men and women (this condition is automatically met when there is only one toilet with a single toilet seat).<br>(2) There are walls, doors, curtains, or other view barriers at the entrance.<br>(3) Different toilet seats are separated by curtains or partitions (when there is only one toilet seat in the toilet, this condition is automatically met).	Privacy	IS; IE; OT; OE	M2
Public Bathroom, PB (8)	58	The care facility is equipped with public bathrooms, meeting the following conditions:<br>(1) Equipped with at least one public bathroom.<br>(2) Each high-care unit is equipped with a public bathroom.	Functional Support, Accessibility	IS; IL; IE; OE	M3
	59	The public bathroom is barrier-free, meeting the following conditions:<br>(1) The entrance has no threshold or has a height difference ≤15mm, with a sloping transition.<br>(2) Width of doorway ≥0.8m, allowing the mechanical bath bed to access.	Physical Frailty Support	IS; IL; IE; OT; OL; OE	M3
	60	The public bathroom is safe for having a bath, meeting the following conditions:<br>(1) The floor is even, slip-resistant, easy to clean, and well-drained, with no pooling water.<br>(2) Equipped with shower facilities suitable for residents, and are clearly signed with hot and cold-water marks.<br>(3) There are bath chairs suitable for residents, allowing the elderly to sit during having a bath.<br>(4) Necessary handrails are installed in the shower area and entrance to assist residents.	Safety and Health, Physical Frailty Support	IS; IL; IE; OT; OL; OE	M3
	61	There is a restroom or a toilet inside or nearby the public bathroom, allowing resident to go to the toilet during having a bath.	Functional Support	IS; IL; IE; OE	M2
	62	The public bathroom is equipped with the wardrobe, benches, electric sockets, the sink, and other facilities to meet the resident's needs for changing clothes, drying hair, and personal hygiene.	Functional Support	IS; IL; IE; OE	M2
	63	The public bathroom allows the staff to assist residents with bathing, meeting the following conditions:<br>(1) There is sufficient supporting space near the bath area, allowing staff to assist residents.<br>(2) There is sufficient space for bathing aids like mechanical bath bed to access and use, providing chances for residents to bathe in a lying position.	Service Support, Functional Support	IS; IL; IE; OT; OL; OE	M3
	64	The public bathroom ensures the resident's privacy during having a bath, meeting the following conditions:<br>(1) There are walls, doors, curtains, or other view barriers at the entrance.	Privacy	IS; IE; OT; OL	M2

		(2) Different shower units are separated by curtains or partitions (when there is only one shower unit in the bathroom, this condition is automatically met).			
	65	The care facility is equipped with a hairdressing and barbering room or space to meet the needs of residents.	Functional Support	IS; IE; OL; OE	M1
Entry Lobby, EL (4)	66	The entry lobby is equipped with a consultation space, which includes a small meeting room, to meet the needs of residents, their families, and staff for check-in and private conversations.	Work Support	IS; IE; OE	M3
	67	The entry lobby has a reception space that is clearly visible from the main entrance.	Work Support, Accessibility	IS; IE; OE	M3
	68	The entry lobby is equipped with chairs or sofas to meet the needs of residents and visitors for waiting, resting and communicating.	Social-Recreational Support, Functional Support	IS; IL; IE; OT	M2
	69	The entry lobby has space for announcements, publicity, and promotions, such as bulletin boards.	Functional Support, Home likeness	IS; IE	M3
Dining Space, DS (10)	70	The dining space is easily accessible for residents and near the kitchen or meal elevator, ensuring convenient meal delivery that does not pass through outdoor areas.	Accessibility	IS; IL; IE; OL; OE	M3
	71	The dining space is equipped with adequate seats.	Functional Support	IS; IE; OT; OE	M3
	72	There is a handwashing sink located near the dining space, making it convenient for residents to wash their hands and rinse their mouths before and after meals.	Functional Support	IS; IE	M2
	73	There is storage space for wheelchairs or walking aids near the entrance of dining space.	Physical Frailty Support	IE; OT	M1
	74	The dining room is equipped with a meal preparation space for staff to prepare meals. The location is reasonable, allowing staff to supervise residents during meal preparation.	Service Support, Work Support	IS; IE; OL; OE	M3
	75	The arrangement of the dining tables and chairs is spacious, allowing diners, wheelchairs and walking aids to access through the corridor without feeling crowded.	Work Support, Physical Frailty Support	IS; IE; OT; OL; OE	M1
	76	The dining tables and chairs meet the following conditions:<br>(1) Sturdy and stable, without sharp edges or corners.<br>(2) Equipped with armrests and backrests.<br>(3) Easy to move and clean.<br>(4) Some dining tables are accessible for wheelchairs or walking aids.	Safety and Health, Physical Frailty Support	IS; IL; IE; OT; OL	M2
	77	The dining space offers different types of tables, such as two-person and four-person tables, catering to individual or group dining preferences for the residents.	Choice and Control	IL; IE; OT; OL; OE	M1
	78	The dining space can meet the different dining needs of residents, meeting at least one of the following conditions:	Choice and	IE; OT; OL; OE	M3

		(1) Equipped with private rooms or other separate dining areas for family gatherings.<br>(2) Equipped with various types of dining spaces such as cafeterias and food bars.<br>(3) Meets the needs of different dining preferences, including self-service, meal delivery assistance, and ordering options.	Control, Home likeness		
	79	The dining space is regular in shape, and the furniture is easy to move, making the space can be used for public activity.	Social-Recreational Support, Functional Support	IL; IE; OL; OE	M3
Activity Space, AS (10)	80	The activity space is easily accessible for residents, including those in cold regions who can reach without going outdoors, and those in rainy regions who can reach through sheltered corridors.	Accessibility	IS; OE; OL	M3
	81	The activity space has a favorable atmosphere to attract residents to join in activities, meeting the following conditions:<br>(1) The activity space has the open interface and layout, making it easy for residents to see and join in activities from the corridor.<br>(2) Most of the activity spaces are close to each other, either on the same floor in adjacent areas or easily accessible by elevator.	Accessibility, Social-Recreational Support	IS; IL; IE; OL; OE	M3
	82	The care facility has public activity spaces at different levels, including open, semi-open, and semi-enclosed spaces in various sizes, ranging from large to small.	Social-Recreational Support, Choice and Control	IL; IE; OT; OL; OE	M1
	83	The care facility has at least four types of public activity spaces as follows:<br>(1) Reading space: equipped with books, latest magazines, and current newspapers for residents.<br>(2) Chess space: provided with chess, mahjong, and other board games commonly enjoyed by residents.<br>(3) Fitness space: equipped with fitness equipment or amenities for residents, such as table tennis, billiards, shuffleboard.<br>(4) Painting space: equipped with painting and calligraphy amenities for residents.<br>(5) Music and dance space: equipped with multimedia equipment.<br>(6) Electronic reading space: equipped with networked computers.<br>(7) Classrooms: equipped with projection facilities or black/white boards.	Social-Recreational Support, Choice and Control	IS; IL; IE; OT; OL	M2
	84	The care facility is equipped with at least one of the following types of public activity spaces:<br>(1) An audio-visual room or screening room.<br>(2) An intergenerational interaction area or children's activity room.<br>(3) A cooking classroom or family kitchen.<br>(4) Other spaces for residents to engage in activities of interest (e.g. handicraft room, tea ceremony room, gardening room, etc.).	Social-Recreational Support, Choice and Control	IS; IL; IE; OT; OL; OE	M3

		(5) Space for religious worship.<br>(6) A swimming pool.			
	85	The care facility is equipped with spaces for visitors to rest and gather with residents.	Home likeness, Privacy	IL; IE; OT; OL; OE	M1
	86	The care facility is equipped with activity rooms (like multi-function halls) for holding large group activities (like carnivals) for residents.	Social-Recreational Support	IS; IE; OT; OL; OE	M3
	87	The activity room (like multi-function hall) meets at least three of the following conditions:<br>(1) Equipped with public toilets for residents nearby.<br>(2) Equipped with storage space, pantry and backstage space.<br>(3) Equipped with television or projection facilities.<br>(4) Equipped with stage with lighting and sound equipment.	Functional Support, Work Support	IS; IE; OT; OL; OE	M2
	88	The activity space is regular in shape, and the furniture is easy to move, making the space can be used for multiple activities at different time.	Social-Recreational Support, Choice and Control	IL; IE; OT; OL	M1
	89	The care facility has the activity space for local community residents to use.	Social-Recreational Support	IL; OT; OE	M1
Entrance, Corridor, Staircase & Elevator, ECSE (20)	90	The care facility has independent external entrance that do not directly open onto high-traffic roads, ensuring the safety of residents.	Accessibility, Safety and Health	IS; IE	M3
	91	The entrance area is paved with non-slip materials to prevent water pooling, and anti-icing measures are implemented in cold areas.	Safety and Health	IS; OT; OL	M3
	92	The main entrances are either level-slope entrances or entrances with steps and wheelchair ramps (or elevated platforms) equipped with handrails on both sides.	Physical Frailty Support	IS; IL; OL	M2
	93	The main entrance door meets the following conditions:<br>(1) Width of doorway ⩾ 1.1m. If two or more doorways are included, at least one width of doorway ⩾ 0.8m to allow wheelchair access.<br>(2) The entrance can be opened as a swing door or a motorized sliding door, but not as a revolving door.<br>(3) The door is easily opened and does not shut quickly, ensuring the safety of residents.<br>(4) The entrance has no threshold or has a height difference ⩽15mm, with a sloping transition.	Physical Frailty Support, Safety and Health	IS; IL; IE; OL	M2
	94	The entrances in cold regions are equipped with door buckets or side entrances to block cold winds.	Comfort	IL; IE	M3
	95	The main entrance depth ⩾ 1.5m, making it convenient for waiting and wheelchair turning.	Physical Frailty Support	IS; IE	M2
	96	Equipped with storage space for wheelchairs and walking aids near the entrance.	Physical Frailty Support	IS; IE	M3

97	The main entrances have canopies to cover waiting areas.	Functional Support	IS; IE; OT; OE	M2
98	Floors of public corridors used by residents have no thresholds or height differences, or have height differences ⩽15mm, with sloping transitions.	Physical Frailty Support, Safety and Health	IS; IL; OT; OL; OE	M2
99	Width of public corridors used by residents ⩾ 1.8m. Corridors between 1.4 and 1.8 meters wide have designated wheelchair passing spaces that are at least 1.8 meters wide.	Physical Frailty Support	IS; IL; IE; OT; OL	M3
100	Public corridors used by residents are equipped with handrails at a height of 0.8-0.9m from the ground, securely installed, slip-resistant, warm to touch, and easy to grip.	Physical Frailty Support	IS; IL; IE; OT; OL; OE	M3
101	Public corridors used by residents offer clear directional guidance and wayfinding cues, providing residents navigations to different areas. The ends of public corridors are featured with cues or decorations to prevent a "dead-end" feel.	Accessibility, Home likeness	IL; OT; OL; OE	M1
102	Public corridors have partial widened areas, equipped with seats for residents to rest.	Social-Recreational Support	IL; IE; OT; OL; OE	M1
103	Residents on the basement, second floor, or above have easy access to elevators.	Physical Frailty Support	IS; IL; OL	M3
104	Elevators used by residents are barrier-free elevators with a cage depth of ⩾1.4m, width of ⩾1.1m, a cage door opening width of ⩾0.8m, and an elevator door width of ⩾0.9m.	Physical Frailty Support	IS; IL; IE	M3
105	The care facility has at least one stretcher-accessible elevator.	Physical Frailty Support, Safety and Health	IS; IE; OL	M3
106	At least one of the elevator cages is barrier-free (if any), meeting three or more of the following conditions:<br>(1) Equipped with operating displays and arrival prompts.<br>(2) The height and design of floor selection buttons are easy for residents to recognize and operate.<br>(3) Handrails are installed on cage's sidewalls, and have no collisions with users.<br>(4) Elevators are well-lit to help residents watch the floor and operating buttons while going in and out.	Physical Frailty Support, Safety and Health	IS; IE; OL	M3
107	The elevator hall is equipped with seats for residents to rest.	Social-Recreational Support	IL; IE; OT	M3
108	The care facility equipped with stairs on the second and higher floors, basements and semi-basements, meeting the following conditions:<br>(1) Not curved or spiral stairs.<br>(2) Width of stairs ⩾ 1.2 m.<br>(3) Equipped with handrails at a height of 0.8-0.9m from the ground, securely installed, slip-resistant, warm to touch, and easy to grip.<br>(4) The first step is distinctly marked or have warning signs.	Physical Frailty Support, Safety and Health	IS; IL; IE; OL	M3

	109	The stair steps are age-friendly (if any), meeting the following conditions:<br>(1) Steps are regular and uniform (no fan-shaped steps), with no steps on the stair platforms.<br>(2) The height and width of steps within the same stair are consistent.<br>(3) Each step has a riser (kickplate).<br>(4) The front edge of the step protruding ⩽10mm, equipped with a slip-resistant strip protruding ⩽ 3mm, not hindering resident's stepping.	Safety and Health	IS; IE; OT	M2
Medical & Rehabilitation Space, MRS (13)	110	The medical space has an emergency corridor for transfer of residents to ambulances or emergency entrances in urgent situations.	Safety and Health, Accessibility	IS; IE	M2
	111	The care facility equipped with separated pathway for emergency medical evacuation and removal of mortal remains that do not go through resident areas.	Comfort, Privacy	IS; IE; OL	M3
	112	The medical space is easily accessible for residents, the internal space allows wheelchairs or walking aids to access, turn around and parking.	Physical Frailty Support, Accessibility	IS; IL; IE; OT; OL	M3
	113	The medical space meets the following conditions:<br>(1) The floor area of infirmary ⩾ 40 ㎡, including at least a consultation room, a treatment room, and a disposal room. The usable area of treatment room and disposal room ⩾ 10 ㎡.<br>(2) The floor area of nursing station ⩾ 30 ㎡, including at least a treatment room and a disposal room.	Functional Support, Work Support	IS; IL; IE; OL	M2
	114	The medical space has a dispensing room for storing common and personal medicines for residents. This room is equipped with medicine cabinet and table for dispensing medicines, which can be locked.	Work Support	IS; IL; IE	M2
	115	The care facility is equipped with a separate area for hospice services (e.g., hospice room, hospice area, etc.) near the pathway for human remains. The hospice area is strategically designed to avoid interference with surrounding environment.	Work Support, Comfort	IS; IE	M3
	116	The care facility is equipped with an independent or a combined assessment room with tables, chairs, and equipment like assessment steps, for evaluating the capabilities of residents.	Functional Support, Work Support	IS; IE	M3
	117	The care facility is equipped with storage space for health and hospitalization records of residents, meeting the staff's needs for access and recording of information. If electronic records are used, there should be room for computers.	Work Support	IS; IE	M3
	118	The care facility has a space for temporary storage of medical waste and is equipped for collecting, washing, and sterilizing the waste. There is also a separate transportation route for medical waste.	Work Support, Safety and Health	IS; IE	M3
	119	Equipped with a public toilet for residents near the medical space.	Functional Support	IS; IE	M2
	120	The care facility is equipped with a rehabilitation space for residents, meeting the following conditions:<br>(1) Equipped with rehabilitation equipment suitable for residents with different functional limitations.<br>(2) Equipped with a handwashing sink.<br>(3) The arrangement of rehabilitation equipment is reasonable, without safety hazards.<br>(4) The layout is flexible for different rehabilitation equipment.	Functional Support, Work Support	IS; IL; IE; OL	M3

		(5) The rehabilitation space is barrier-free, the equipment is accessible for wheelchairs or walking aids.<br>(6) Equipped with storage space for wheelchairs and walking aids.			
	121	The floor of rehabilitation space is even, slip-resistant and protective.	Safety and Health	IS; IE; OT; OL	M2
	122	The care facility is equipped with a specialty rehabilitation space, meeting at least one of the following conditions:<br>(1) A cognitive rehabilitation space for group activities, music therapy, nostalgia therapy, and sensory stimulation to facilitate cognitive rehabilitation.<br>(2) A cultural and sports rehabilitation space for targeted sports and recreational activities.<br>(3) A therapeutic rehabilitation landscape that that stimulates the senses and allows for activities like horticultural therapy.<br>(4) Other specialized rehabilitation spaces such as Chinese medicine physical therapy rooms.	Home Likeness, Choice and Control	IS; IE	M2
Central Kitchen, CK (4)	123	The care facility is equipped with kitchens or offers external dining services, thereby meeting the residents' dining needs.	Work Support	IS; IE; OE	M3
	124	The central kitchen is equipped with a separate entrance for delivery of goods, disposal of food waste, and staff access.	Work Support, Accessibility	IS; IL; IE; OE	M3
	125	The central kitchen is equipped with reasonable size for storage, processing, and handling of food ingredients with essential areas such as incoming, warehouse, processing, meal preparation, and recycling.		IL; IE; OL	M3
	126	The central kitchen is sectioned into clean and dirty zones to ensure the functional organization is reasonable.	Work Support	IL; IE	M3
Laundry Space, LS (2)	127	The care facility meets the laundry needs of residents, meeting at least one of the following conditions:<br>(1) Equipped with a central laundry room.<br>(2) Equipped with public laundry rooms in the care units.<br>(3) Equipped with washers in resident rooms.<br>(4) Outsourcing laundry services.	Work Support	IS; IE; OE	M3
	128	The public laundry space caters to the temporary storage, washing and drying (tumble drying) of clothes, meeting the following conditions:<br>(1) Equipped with storage or countertop spaces, sectioned into clean and dirty zones.<br>(2) Equipped with washers, sinks, disinfecting gear to meet the laundry needs.<br>(3) Equipped with drying spaces or available drying equipment.	Work Support, Functional Support	IS; IE; OE	M3
Cleaning Space, CS (2)	129	The care facility has a particular cleaning space for soiled items near the filth-transporting corridor or elevator for efficient waste removal.	Work Support	IS; IE; OE	M3
	130	The cleaning space has at least three of the following functions:<br>(1) Cleaning and disinfecting soiled items.<br>(2) Storing soiled items (garbage) temporarily.<br>(3) Storing detergents.<br>(4) Storing cleaning tools like rags, mops, and cleaning carts.	Work Support	IS; IE; OE	M2

		(5) Drying cleaning tools like rags and mops.			
Public Storage Space, PSS (3)	131	The care facility is equipped with centralized public storage space or room.	Work Support	IS; IE; OT; OE	M2
	132	The care facility is equipped with centralized garbage storage space near staff entrances to minimize the impact of smells and transportation on residents.	Work Support	IS; IL; IE; OE	M2
	133	The public storage space is ample in quantity (or size), equipped with shelves and cabinets, and capable of storing various categories of items. There are no stacked things within the care unit, making the space clean and safe for evacuations.	Work Support	IS; IE; OT	M3
Staff Working Space, SWS (5)	134	The care facility is equipped with social worker studios or work areas that are combined with other spaces.	Work Support	IS; IE	M2
	135	The care facility is equipped with space for doctors' and rehabilitators' offices, as well as areas for changing, resting, and storage of rehabilitation therapy items.	Work Support, Staff Facilities	IS; IE; OE	M3
	136	The care facility is equipped with staff offices or office areas, including duty rooms, administrative offices, finance rooms, and dean's offices. These spaces are well-equipped to meet the work requirements of staff.	Work Support, Staff Facilities	IS; IE; OT; OE	M2
	137	The care facility is equipped with space for staff meeting and training.	Work Support	IE; OT; OE	M3
	138	The staff working space is located close to the activity space or living rooms for easy access by residents.	Work Support, Accessibility	OT; OL; OE	M1
Staff Living Space, SLS (1)	139	The care facility is equipped with designated areas for staff dining, rest, and personal hygiene, thereby catering to the essential daily needs of the staff.	Staff Facilities	IS; IE; OT; OL; OE	M3
Signage System, SS (4)	140	Emergency guidance signs are strategically located to provide clear and consistent guidance for residents in a uniform style. Safety warning signs or design elements are placed to catch the attention of residents for potential dangers.	Safety and Health, Accessibility	IS; OT	M3
	141	Access guide signs and service-oriented signs are strategically located to provide clear and consistent guidance for residents in a uniform style.	Accessibility	IS; OT; OL; OE	M3
	142	All types of signs meet the following conditions:<br>(1) Be securely installed, have no defect that might pose a safety risk on residents.<br>(2) Be accurately positioned, have high visibility for residents, avoiding being sheltered from lights, cameras and plants, and have no influence on the use of other facilities.<br>(3) Be designed in view of resident's visual characteristics, using larger texts and high-contrast colors for easy recognition.	Accessibility, Physical Frailty Support	IS; OT; OL; OE	M2
	143	Signage system is designed to match the care facility's style in form and material, maintaining consistency with the environmental decor while balancing functionality with aesthetics.	Home Likeness	IS; OL; OE	M1
Fire Protection Facilities, FPF (2)	144	The care facility's outdoor area is equipped with fire breaks, lanes, and designated sites for fire truck boarding and operating. The care facility is equipped with fire extinguishers, automatic fire alarms, sprinkler systems, and emergency lightings as required by fire protection regulations.	Safety and Health	IS; IE	M2
	145	Fire protection facilities and equipment are strategically placed to avoid interference to daily operations in care facilities.	Safety and Health	IS; IE	M3

Lighting, Ventilation, Temperature & Sound, LVTS (9)	146	The care facility is equipped with temperature and humidity control equipment to maintain a comfortable environment.	Comfort	IS; IL; IE; OT; OL; OE	M3
	147	Spaces primarily used by residents, such as resident rooms, corridors, and living rooms, have operable windows and conditions for natural daylight.	Comfort	IS; IL; IE; OT; OE	M3
	148	Resident rooms, living rooms, and other spaces frequently used by resident can benefit from natural light.	Comfort	IS; IE; OT; OE	M3
	149	The artificial illumination is sufficient, even, adjustable, and the lighting has no glare and easy to maintain.	Comfort	IS; IL; IE; OT; OL; OE	M3
	150	The lighting in spaces used by residents is designed to create a home-like atmosphere.	Comfort, Home Likeness	IS; IL; IE; OT; OL; OE	M3
	151	Resident rooms, corridors, and living rooms are equipped with operable windows that provide effective natural ventilation to keep the environment fresh and free of odors.	Comfort	IS; IL; IE; OT; OL; OE	M3
	152	Public bathrooms, laundry rooms, and other support areas are equipped with operable windows or mechanical ventilation systems with backflow prevention to keep the environment fresh and free of odors.	Comfort	IS; IE	M3
	153	Each functional area has a good acoustic environment, and larger spaces like the living room are free from significant noise reverberation.	Comfort	IS; OT; OL; OE	M3
	154	Resident rooms have a good acoustic environment, meeting the following conditions:<br>(1) Not located next to elevators, noisy equipment rooms, or other similar areas.<br>(2) Have effective sound insulation, ensuring that residents are not disturbed by external noises when resting in their rooms.	Comfort	IS; OL; OE	M3
Home-Like Qualities, HLQ (6)	155	Resident rooms, living rooms, and corridors are enriched with elements such as cozy furniture, decorations, and indoor plants, creating a home-like atmosphere.	Home Likeness	IS; IE; OT; OL; OE	M3
	156	The care unit has a home-like scale and the warm interior design.	Home Likeness	IS; IL; IE; OT; OL; OE	M1
	157	The entry lobby has a warm and bright interior design, making people feel welcome.	Home Likeness	IS; IL; IE; OT; OL	M3
	158	The dining space is furnished with cozy decorations to stimulate the appetites of residents.	Home Likeness	IS; IL; IE; OT; OL	M3
	159	The medical and rehabilitation space has a comfortable and calming atmosphere.	Home Likeness	IS; IE; OE	M3
	160	All functional spaces are well-maintained, clean, and tidy.	Home Likeness, Comfort	IS; IE; OT	M3
