# Supplementary material for: Improving life quality for the aged: a comprehensive post-occupancy evaluation of long-term care facilities in China
Source: Front Public Health. 2024 Nov 28;12:1488653. doi: 10.3389/fpubh.2024.1488653 (PMC11636290; doi:10.3389/fpubh.2024.1488653)
Supplement: Supplementary file 3 [file Data_Sheet_3.pdf]

Supplementary Material C

Physical Environmental Problem List of LTCF in China.

Environmental Design Elements	No.	Description of Typical Problems
Traffic Condition Surrounded, TCS	01	The current parking capacity for both cars and bicycles is inadequate, resulting in inconveniences associated with parking.
	02	The limited availability of public transportation in the vicinity poses challenges for commuting and outing.
	03	The intersection of the vehicle traffic area and the outdoor activity spaces poses a potential safety hazard.
Supporting Facilities Surrounded, SFS	04	The surrounding area lacks essential supporting facilities such as hospitals, parks, supermarkets, etc.
Outdoor Space, OS	05	The outdoor space lacks landscape design.
	06	The area of the outdoor activity space is inadequate to accommodate the residents' preferred outdoor activities.
	07	The outdoor space's location is inefficient, or has height differences in entrances, causing inconvenience for residents with wheelchairs or walking aids.
	08	The outdoor space has insufficient supporting facilities, such as sunshades, seating, walking paths, and exercise equipment for residents.
Building Scale, BS	09	Average floor area per bed is substandard.
	10	The capacity of the care facility is inappropriate, either too large (making it difficult to manage and provide services) or too small (failing to meet the daily needs of residents and staff).
	11	The capacity of the care unit is inappropriate, either too large (resulting in a lack of a home-like atmosphere) or too small (failing to meet the daily needs of residents and staff).
Functional Organization, FO	12	The living spaces for residents and the working spaces for staff are not separated, resulting in mutual interference between two groups of users in daily life.
	13	The living spaces for residents are not organized into self-contained care units, which precludes the ability to isolate and prevent cross-infections.
	14	The living spaces for residents are not segregated into specific care units based on individual physical conditions, leading to interpersonal conflicts and challenges in daily life.
	15	The layout exhibits suboptimal design, resulting in inadequate ventilation, subpar lighting conditions, and deficient wayfinding.
Resident Room, RR	16	The lack of single rooms limits residents' choices and privacy.
	17	The multiple rooms lack privacy design.
	18	The area of resident rooms is inadequate, with insufficient room width to meet the daily needs of residents.
	19	The personalized space in resident rooms is insufficient, lacking a home-like atmosphere.
	20	The supporting facilities in resident rooms are insufficient, having no adequate space like a small kitchen, balcony (for drying clothes), attached restroom, and storage space.
	21	The furniture and equipment in the resident rooms are incomplete and inconvenient, such as poorly designed closets and shoe cabinets, the lack of refrigerators, washing machines, water dispensers, tables and chairs, etc.
	22	Furniture and equipment in multiple rooms are not equitably set, without considering fairness.
	23	General details in resident rooms are inconvenient, such as the lack of night lights, poorly positioned switches and electric sockets.
	24	Resident rooms fail to meet barrier-free requirements, such as insufficient width of doorway, and height differences between the resident room and the balcony or the bathroom.
	25	Restrooms within the resident rooms have many problems causing hidden dangers, including the lack of handrails, the lack of separation between wet and dry areas, slippery flooring, the lack of properly equipped emergency call devices, etc.
Living Room, LR	26	The scale of the living room is inappropriate, either too large (e.g. resulting in wasted space) or too small (e.g. failing to provide adequate space for residents’ daily activities).
	27	The supporting facilities in living rooms are insufficient, such as inadequate furniture, the lack of meal preparation spaces, the lack of sinks, etc.

	28	The nursing station is poorly located, with inadequate connection and poor visibility to the living room.
	29	The area of the nursing station is insufficient, making it difficult for the staff to efficiently carry out their services.
	30	The supporting facilities and storage spaces in the nursing station are insufficient.
Public Toilet, PT	31	The facility has an inadequate number and size of public toilets, resulting in a shortage or inconvenience in daily use.
	32	Public toilets have many problems causing hidden dangers, including slippery flooring, the lack of handrails, and unfriendly forms of the hand-washing sinks for residents with wheelchairs and walking aids.
	33	The design details in public toilets are inadequate, such as the unreasonable form of door opening.
	34	The public toilets lack privacy design.
Public Bathroom, PB	35	The location of the public bathroom is not convenient for daily use, far away from the living spaces of residents.
	36	There is no public bathroom or the number of public bathrooms is inadequate, failing to help residents bathe in a lying position.
	37	Public bathrooms fail to meet bathing service modes, leaving underutilized.
	38	Public bathrooms have no proper spaces for drying hair and changing clothes, lacking mechanical bath beds and staff changing rooms nearby.
	39	Public bathrooms present inherent hazards due to the absence of handrails, inadequate drainage systems, and raised thresholds at entrances.
	40	The design details in public bathrooms are inadequate, such as inconveniences in shower's use.
Entry Lobby, EL	41	The scale of the entry lobby is inappropriate, either too large (resulting in wasted space low occupancy, and less homelike atmosphere) or too small.
	42	The supporting facilities in the entry lobby are insufficient, lacking private meeting spaces, seating areas for rest, information display screens, double-layered doors, etc.
Dining Space, DS	43	The dining space's location is inconvenient, leading to longer meal delivery and return pathways.
	44	The dining space is excessively compact and densely furnished, making it difficult for all residents within the care unit to have meals here.
	45	The dining space's supporting facilities are insufficient, lacking meal preparation space, private dining rooms, and handwashing sinks.
	46	The dining tables and chairs are not accessible for wheelchairs or walking aids.
Activity Space, AS	47	The distance between activity spaces and residents' living areas poses challenges for residents in terms of accessibility.
	48	Activity spaces are designed as enclosed rooms, hindering flexible and multifunctional use.
	49	The types of activity spaces are insufficient, such as the lack of a library, a chess room, or a multifunction room.
	50	General details in activity spaces are inconvenient, such as the lack of washbasins in the painting room.
Entrance, Corridor, Staircase & Elevator, ECSE	51	The number of entrances and exits is insufficient, failing to maintain cleanliness and prevent cross-contamination.
	52	Entrances and exits fail to comply with barrier-free requirements, exhibiting excessive variations in height, steep inclines, and absence of guardrails.
	53	The width of public corridors is narrow, making it difficult for residents with wheelchairs or walking aids to pass through side by side.
	54	The public corridors exhibit suboptimal wayfinding cues and an excessive number of intersections, posing challenges for residents in orienting themselves.
	55	Elevators are unreasonable categorized, such as the lack of medical elevators, the lack of stretcher-accessible elevators, and a mix of residents and service elevators.
	56	Elevators fail to meet barrier-free requirements, exhibiting inconveniences in using floor selecting bottoms and handrails.
	57	The stair steps are not distinctly marked, creating a safety risk.
Medical & Rehabilitation Space, MRS	58	The medical space remains underutilized due to a mismatch between the space pattern and the caregiving mode.
	59	The medical space is far away from the residents' living spaces, rendering its location inconvenient for residents.
	60	The scale and equipment of medical space are inadequate to meet the medical needs of residents and the working needs of staff.
	61	The supporting facilities in the medical space are insufficient, such as the lack of treatment rooms, pharmacies, assessment rooms, etc.

	62	The scale and equipment of the rehabilitation space are inadequate to meet the rehabilitation needs of residents and the working needs of staff.
	63	The supporting facilities in the rehabilitation space are insufficient, such as the lack of traditional Chinese medicine treatment areas, unsuitable rehabilitation devices for residents, etc.
	64	The rehabilitation space is poorly designed, such as the slippery flooring, the lack of washbasin, etc.
	65	The medical and rehabilitation space lacks privacy design.
Central Kitchen, CK	66	The location of the central kitchen is unreasonable, such as being too close to the living spaces of residents, or being underground spaces with poor hygiene and ventilation conditions.
	67	The scale of the central kitchen is inappropriate, resulting in inconvenient workflow and an inability to ensure the quality of the meals.
	68	The supporting facilities in the central kitchen are insufficient, such as the lack of food storage rooms, liquid food production rooms, etc.
Laundry Space, LS	69	The location of the laundry space is unreasonable, resulting in longer travel distances for laundry routes or being situated in poorly ventilated underground spaces.
	70	The scale of the laundry space is inappropriate, failing to maintain cleanliness and prevent cross-contamination.
	71	The supporting facilities in laundry space are insufficient, such as the lack of drying and folding spaces.
Cleaning Space, CS	72	The location of the cleaning space is unreasonable, resulting in longer workflow paths for cleaning.
	73	The cleaning space is too small in size, interacting with other areas, failing to maintain cleanliness and prevent cross-contamination, etc.
	74	The cleaning space lacks adequate supporting facilities, failing to meet the requirements for disinfecting soiled items and storing cleaning tools.
Public Storage Space, PSS	75	The location of the public storage space is unreasonable, such as being underground spaces with humid environment.
	76	The public storage space is small in size and limited in type, with a lack of floor-specific storage rooms and waste disposal areas.
Staff Working Space, SWS	77	The location of the staff working space is unreasonable, such as being too dispersed resulting in low work efficiency, being far away from the residents' living spaces, being underground spaces with poor lighting conditions and humid environment, etc.
	78	The size and type of staff working spaces are insufficient, such as the lack of centralized office areas, medical and nursing offices, staff break rooms, training and meeting spaces, etc.
Staff Living Space, SLS	79	The staff living spaces is poor designed, lacking privacy design.
	80	The number and scale of staff living spaces is inadequate, causing detrimental impact on the well-being of staff and, consequently, on the quality of care provided.
	81	The supporting facilities in staff living spaces are insufficient, such as the lack of staff dining spaces, changing and shower spaces, recreational and exercise spaces.
Signage System, SS	82	Signage systems exhibit inconsistency due to an excessive variety of forms.
	83	Signage is deficient or inadequately conspicuous.
Fire Protection Facilities, FPF	84	The design of the refuge areas is incompatible with the regulation, for example, refuge areas are misused as resident rooms.
	85	Fire protection facilities pose inconveniences for residents and staff, such as the presence of fire extinguishers in corridors impeding access.
Lighting, Ventilation, Temperature & Sound, LVTS	86	Functional spaces have uncomfortable temperatures, such as temperatures being low in north-facing rooms in winter, cold air entering the entry lobby from the outside and causing low temperatures in winter, etc.
	87	The artificial illumination is not adjustable.
	88	The natural lighting conditions in functional spaces are poor, such as insufficient sunlight in north-facing rooms, excessive sun exposure in west-facing ones, and poor lighting conditions in corridors and living rooms.
	89	The ventilation conditions in functional spaces, such as resident rooms, living rooms, dining spaces, and activity spaces, are suboptimal. Similarly, the ventilation conditions in toilets within resident rooms, public toilets, public bathrooms, and laundry spaces are also inadequate.
	90	Functional spaces exhibit suboptimal acoustic conditions, characterized by the presence of noisy resident rooms in close proximity to the road and a lack of adequate acoustic separation in activity areas.
Home-Like Qualities, HLQ	91	The physical environment lacks home-like qualities and warm interior design. The decor exhibits more resemblance to that of a hotel or dormitory rather than a domestic space.
	92	The hygiene conditions in public spaces are poor, such as public toilets, corridors, and cleaning spaces, which are not clean and tidy.
